# Two-photon excited circularly polarized luminescence of hetero[9]helicene with multiresonant thermally activated delayed fluorescence

**DOI:** 10.1038/s41467-026-77554-5

**Published:** 2026-09-15

**Authors:** Sen Wu, Yan Xu, Dominic J. Black, Aidan P. McKay, David B. Cordes, Robert Pal, Eli Zysman-Colman

**Affiliations:** 1 Organic Semiconductor Centre, EaStCHEM School of Chemistry, University of St Andrews, St Andrews, KY16 9ST UK; 2 College of Physics and Optoelectronics, Taiyuan University of Technology, Taiyuan, 030024 P. R. China; 3 Key Laboratory of Luminescent Materials and Devices, School of Materials Science and Engineering, South China University of Technology, Guangzhou, 510640 P. R. China; 4 Department of Chemistry, Durham University, South Road, Durham, DH1 3LE UK

**Keywords:** Materials for optics, Circular dichroism, Optical materials

## Abstract

Circularly polarized luminescence (CPL) imaging of biomolecules using designed chiral probes holds significant promise for biochemical and analytical applications. In this work, we report two chiral hetero[9]helicene multi-resonant thermally activated delayed fluorescent (MR-TADF) organic molecules **DDCzBPh** and **DDCzBPy**, consisting of two annulated MR-TADF units to a central naphthyl or quinoxanyl group, respectively, which exhibit simultaneously strong CPL, two-photon absorption, high photoluminescence quantum yields, and long-lived delayed fluorescence. We demonstrate two-photon-excited CPL of TADF emitters, with their circularly polarized brightness of 0.2 GM, demonstrating their potential for CPL biological imaging.

## Abstract (web summary)

The design of chiral probes for circularly polarized luminescence imaging of biomolecules is relevant for different applications. Here, the authors report chiral hetero[9]helicene multi-resonant thermally activated delayed fluorescent organic molecules showing strong two-photon circularly polarized luminescence with potential application in biological imagining.

## Introduction

Chiral materials that emit circularly polarized luminescence (CPL) have attracted significant attention due to their applications in sensing^1^, security^2^, and optical communications^3^. Recent advance in CPL spectroscopy and microscopy have expanded their utility in biological imaging, encoding vital chiral molecular fingerprints within photoluminescence (PL) spectra^4–6^. Traditionally, emitters are excited into a low-energy absorption band, relying on single-photon excitation. Most emitters have absorption onsets that require high-energy photons for excitation, which limits their biological applications, as they often cause cellular damage and suffer from limited penetration depth according to the modified Beer-Lambert law^7^. This limitation can be overcome by employing two-photon excitation (2PE), where molecules are excited by two sequential photons of half the energy of the absorption band. In addition, long-lived emission is strongly desirable in bioimaging, as this allows for time-gated detection methods to be used that increase the signal-to-noise and permit the exclusion of the short-lived autofluorescence background (with lifetimes on the nanosecond scale)^8^. Consequently, developing imaging agents that are chiral, have long-lived PL, and that can be excited by 2PE are desirable. To date, reports on 2PE-CPL emitters remain scarce. Recently, several Eu(III) complexes exhibiting 2PE-CPL properties have been reported, demonstrating exceptional chiroptical properties, narrow emission bands, and millisecond-long PL lifetimes^4–6^. However, the development of organic compounds possessing both 2PE-CPL properties and comparable photophysical characteristics remains largely unexplored.

Multi-resonant thermally activated delayed fluorescence (MR-TADF) compounds are one class of emitters that show high photoluminescence quantum yield (*Φ*_*PL*_) and long PL lifetimes, providing an excellent scaffold for developing 2PE-CPL materials^9^. Compared to conventional donor-acceptor TADF materials, MR-TADF compounds exhibit narrowband emission and longer delayed luminescence lifetimes, primarily due to their rigid polycyclic aromatic hydrocarbon (PAH) frameworks and moderate singlet and triplet energy gap (Δ*E*_*ST*_)^10,11^. To date, dozens of circularly polarized MR-TADF (CP-MR-TADF) emitters have been reported. However, like most small molecules, these compounds have relatively low PL dissymmetry factors (*g*_*PL*_), typically smaller than 2 × 10^–3^ (Supplementary Fig. 1). The g_PL_ factor describes the chiroptical strength of the CPL and is expressed as: *g*_*PL*_ = 2(*I*_*L*_ – *I*_*R*_)/(*I*_*L*_ + *I*_*R*_), where *I*_*L*_ and *I*_*R*_ are the PL intensities of left- and right-CP emission, respectively. Thus, g_PL_ values range from −2 to +2 for perfectly right- or left-CP emission, respectively, and 0 for the unpolarized or linearly polarized light. Recently, a more comprehensive metric, circularly polarized brightness (CPB), has been introduced to quantify the total number of circularly polarized photons emitted. CPB integrates three key parameters: the molar absorption coefficient (*ε*_*abs*_), *Φ*_*PL*_, and *g*_*PL*_ values, and is formulated by the equation: CPB = *ε*_*abs*_⋅*Φ*_*PL*_⋅(*g*_*PL*_/2)^12^. Among CP-MR-TADF emitters, those with a helical skeleton generally show higher *g*_*PL*_ values (>10^−3^) compared to other types of CP-MR-TADF emitters (Supplementary Fig. 1). Amongst these, **BN[9]H** (denoted as **DDCzBPh** in the current study) has one of the strongest CPL signals in solution^13,14^, exhibiting |*g*_*PL*_| up to 5.8×10^−3^ and CPB of 220.75 M^−1^ cm^−1^ in toluene, and showing a delayed fluorescence lifetime, *τ*_*d*_, of 51.5 μs in 2 wt% doped films in DMIC-TRZ. Its superior chiroptical properties were attributed by the authors to an intrinsic extended helical skeleton. It is worth noting that increasing the helicity of hetero-helicenes has become a go-to strategy to improve *g*_*PL*_ values^15^; however, this strategy poses significant synthetic challenges and risks compromising the Φ_PL_. Thus, careful molecular design is required to balance both.

For a 2PE process, the two-photon absorption (2PA) cross-section (*σ*^*2*^, with the unit of GM, 1 GM = 10^−50^ cm^4^ s photon^−1^), quantifying the probability of the 2PA process, is the key parameter to evaluate the performance of emitters exhibiting 2PA characteristics. The 2PA cross-section is typically several orders of magnitude smaller than the one-photon absorption cross-section^16^. A parameter that can be used to quantify the two photon excited CPL is the two photon excited-CPB (2PE-CPB), which can be defined as: 2PE-CPB = *σ*^*2*^⋅*Φ*_*PL*_⋅(*g*_*PL*_/2)^4^. In principle, an extended π-conjugation framework can lead to compounds having large transition dipole moments, and therefore high Φ_PL_ and large 2PE-CPB^17^. To date, most reported 2PA-TADF emitters are donor-acceptor (D-A) TADF compounds (Supplementary Fig. 2), which typically require a highly twisted conformations to achieve the small Δ*E*_ST_ and efficient reverse intersystem crossing (RISC) required for TADF. However, the twisted conformation leads to modest transition dipole moments and generally lower *Φ*_*PL*_, and consequently low 2PA *σ*^*2*^. MR-TADF emitters, on the other hand, typically have very high *Φ*_*PL*_ as they are based on rigid planar structure and have emissive excited states of short range charge transfer (SRCT) character^18^. This combination of features leads to MR-TADF compounds having moderately small Δ*E*_*ST*_ and attractive, potentially enhanced 2PA characteristics. However, to date the 2PA properties of only three MR-TADF molecules, **DABNA-2,** **CzBN**, and **CzBSe**, have been reported, showing small 2PA *σ*^*2*^ of 63, 35, and 53 GM at 740 nm^19^.

**Fig. 1:**
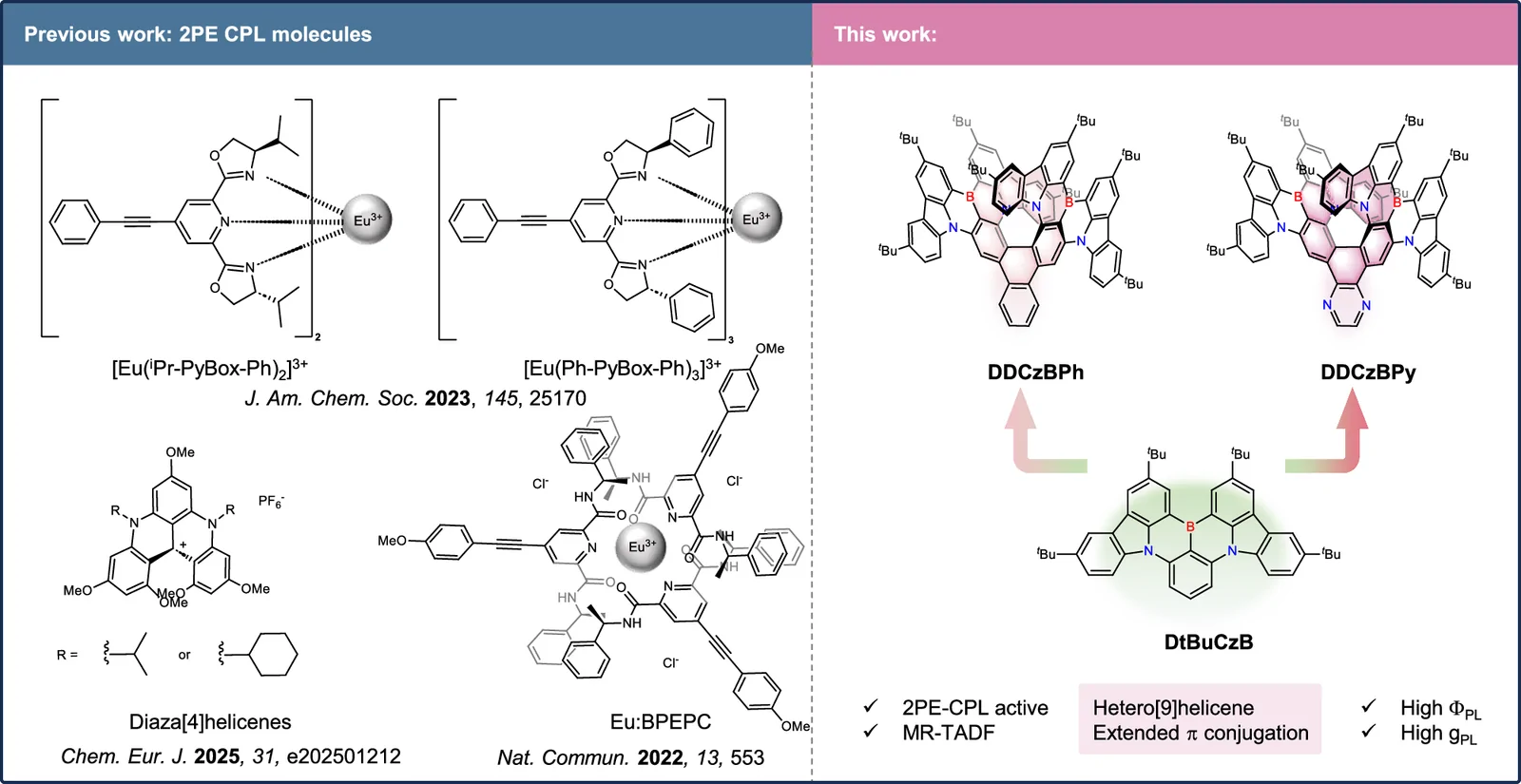
Molecular structures. Previously reported 2PE CPL active molecules and hetero[9]helicenes **DDCzBPh** and **DDCzBPy** in this work.

Reports of organic molecules showing 2PA and CPL are likewise scarce, likely due to the requirement for bespoke instrumentation^20–22^. Since the breakthrough in 2PE CPL spectroscopy and CPL Laser Scanning Confocal Microscope achieved by Pal and co-workers^4^, one recent report has focused on organic 2PE CPL probes^23^. There, a series of functionalized cationic chiral diaza[4]helicenes having high *Φ*_*PL*_ of around 70% in acetonitrile showed one photon exciton (1PE) *g*_*PL*_ values of +5.8/–6.7 × 10^–4^ and 1PE-CPB of 3.4 M^−1^cm^−1^. 2PA properties were confirmed by the intensity-excitation power dependence, and 2PA *σ*^*2*^, when excited at 810 nm (*σ*_*810*_^*2*^), reached 137 GM. The corresponding 2PE *g*_*PL*_ values are +9.4/–9.2 × 10^−4^, which were comparable to those 1PE *g*_*PL*_ values, and the 2PE-CPB values are small at 0.05 GM. These fluorescent probes exhibit lifetimes of approximately 17 ns, which is relatively short compared to long-lifetime lanthanoid probes, such as a recent chiral Eu complex (*Φ*_*PL*_ of 1.6 ms, 2PE-CPB values of 0.16/0.17 GM and high *g*_*PL*_ values of 10^−2^)^6^. Such long lifetimes (μs-ms scale) are advantageous for time-gated luminescence (TGL) by enabling clear temporal separation from short-lived biological background signals like autofluorescence (~ns), significantly improving contrast and sensitivity in bioimaging applications^24^. Here, we report two chiral 2PE-CP-MR-TADF emitters **DDCzBPh** and **DDCzBPy** that have extended hetero-helical skeletons and contain two MR-TADF moieties based on the structure of **DtBuCzB** that are annulated through a naphthyl or a quinoxanyl group (Fig. 1). The extended helicity and π-conjugated framework contribute to their relatively large |*g*_*PL*_| and *σ*^*2*^ values. Furthermore, their moderate Δ*EST* and rigid structure are responsible for microsecond-long delayed PL lifetime and high *Φ*_*PL*_ in toluene. As a result, these two emitters have high *Φ*_*PL*_ of 65 and 80%, high 1PE *g*_*PL*_ values of −5.2 × 10^−3^/ + 5.2 × 10^−3^ for ***P-/M*-DDCzBPh** and −2.9/ + 2.7 × 10^−3^ for ***P-/M*-DDCzBPy**, long delayed lifetimes of 65 and 109.5 μs, and large 2PE *σ*^*2*^ of 118 and 142 GM, respectively. Given their attractive 2PA and CPL properties, 2PE CPL measurements were conducted, and comparable 2PE *g*_*PL*_ values were obtained as −4.6 × 10^−3^/ + 3.3 × 10^−3^ for ***P-/M*-DDCzBPh** and −2.3 × 10^−3^/ + 2.0 × 10^−3^ for ***P-/M*-DDCzBPy**, leading to 2PE-CPB values of 0.20/0.20 GM for ***P-/M*-DDCzBPh** and 0.16/0.15 GM for ***P-/M*-DDCzBPy**. This is the first study of chiral organic MR-TADF compounds showing 2PE CPL.

## Results

### Molecular design and synthesis

The molecular design strategy and synthetic routes are illustrated in Fig. 1 and Supplementary Fig. 3, respectively. Both **DDCzBPh** and **DDCzBPy** incorporate a [9]helicene fragment, constructed from two MR-TADF **DtBuCzB** fragments bridged by either a naphthalene or quinoxaline unit, respectively. The **DtBuCzB** motif was selected due to its near-unity photoluminescence quantum yield and long-lived photoluminescence lifetime of 68.8 μs in 1 wt% doped films in mCBP^25^. Moreover, the parent core **CzBN** that does not contain peripheral *tert*-butyl groups has previously been shown to have 2PA character with a relatively modest *σ*^*2*^ of 35 GM at 740 nm^19^. The extended π-conjugation of **DDCzBPh** and **DDCzBPy** is expected to produce a larger 2PA cross section. The naphthalene or quinoxaline moieties were chosen as the bridging unit to modulated the strength of the intramolecular charge transfer between the bridge and the MR-TADF moiety, thereby modulating both the 2PA cross-section and *g*_*PL*_ factor.

Both **DDCzBPh** and **DDCzBPy** were synthesized following a common procedure to that previously reported for the former^13^, starting from dibromobenzene or dibromopyrazine, respectively. The final step relies on an oxidative Scholl reaction using DDQ^26^, which gave **DDCzBPh** and **DDCzBPy** as orange and red solids in 80 and 74% yield, respectively. The structures of these compounds were verified by ^1^H and ^13^C nuclear magnetic resonance (NMR) spectroscopy, single-crystal X-ray diffraction and high-resolution mass spectrometry (HRMS), and their purity was assessed by melting point determination, elemental analysis (EA), and high-performance liquid chromatography (HPLC) (Supplementary Figs. 4–18). The enantiomers of both compounds were isolated by chiral HPLC with ees of 100% (Supplementary Figs. 10 and 17). The absolute configurations were inferred by comparing the experimental circular dichroism (CD) spectra to the predicted spectra calculated at the TD-DFT-B3LYP/def2-SVP level, including an implicit toluene solvent model (Supplementary Fig. 28). The predicted CD spectrum for ***P*-DDCzBPh** is also in line with that reported in the literature^13^.

**Fig. 2:**
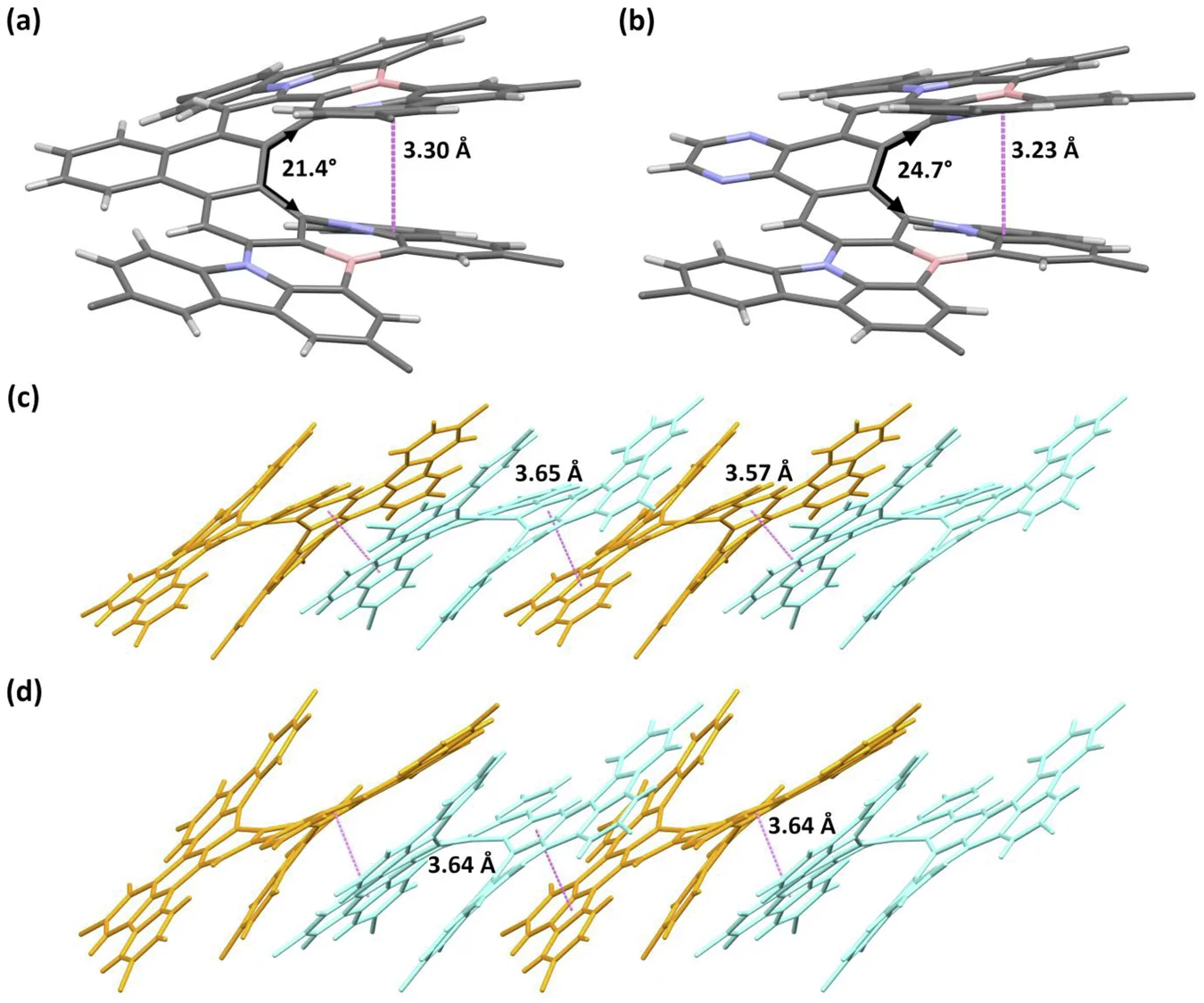
Single-crystal structures. **a** **DDCzBPh** and **b** **DDCzBPy**, each with a single independent molecule shown, and minor components of disorder, *tert*-butyl groups and solvents omitted for clarity in both structures. One representative π···π stacking interaction is indicated. Views of the packing of alternating helicity (orange and light blue) of **c** **DDCzBPh** and **d** **DDCzBPy**, minor components of disorder, *tert*-butyl groups, and solvents omitted for clarity.

Single crystals suitable for X-ray diffraction crystallographic analysis were grown by slow diffusion of ethanol into a toluene solution of racemic **DDCzBPh**, and methanol into a dichloroethane solution of racemic **DDCzBPy**. **DDCzBPh** and **DDCzBPy** crystalize as toluene and dichloroethane solvates, respectively, with both enantiomers present in the asymmetric unit in both structures. The structure of **DDCzBPh** is a toluene solvate of the known structure of the compound **BN[9]H**^13^, and has a very similar molecular geometry. The fused rings bridging the **DtBuCzB** groups show significant distortion (mean deviation from plane 0.073 − 0.087 Å and 0.064 − 0.082 Å, for **DDCzBPh** and **DDCzBPy**, respectively) and with **DDCzBPy** adopting a slightly tighter helical twist (C51-C53-C72-C70 21.4(5)° and C155-C157-C176-C174 22.3(5)°) than that in **DDCzBPh** (C51-C53-C72-C70 24.7(5)° and C155-C157-C176-C174 24.0(4)°) (Fig. 2a and b, Supplementary Table 1). The observed helical structures are reinforced by strong intramolecular π···π stacking (centroid···centroid 3.269(19) − 3.4283(17) Å and 3.2155(19) − 3.3947(18) Å, for **DDCzBPh** and **DDCzBPy**, respectively) between aromatic rings in the two **DtBuCzB** units (Supplementary Fig. 19) and supported by weaker C-H···π interactions (H···centroid 2.6207(14) − 3.316(2) Å and 2.9904(14) − 3.5666(15) Å, for **DDCzBPh** and **DDCzBPy**, respectively) from the *tert*-butyl groups. Their helical geometry makes these compounds promising candidates for chiroptical applications^15^. In both structures, additional weaker intermolecular π···π interactions (centroid···centroid 3.5648(17) − 3.7832(18) Å and 3.6372(18) − 3.8037(18) Å, for **DDCzBPh** and **DDCzBPy**, respectively) between adjacent molecules of alternating helicity (Fig. 2c and d) form chains along [010] in **DDCzBPh** and [100] in **DDCzBPy**, in a similar manner to that seen in the known structure of **DDCzBPh**^13^.

### Theoretical calculations

Theoretical calculations were performed to predict the optoelectronic, chiroptical, and 2PA properties for both compounds. For both compounds, the HOMO/LUMO distribution is reminiscent of the pattern of the MR-TADF emitter **DtBuCzB**^25^, showing LUMOs that are localized on the boron atoms and the carbon atoms *ortho*/*para* to them, whereas the HOMOs are distributed on the nitrogen atoms and the carbon atoms *ortho*/*para* to them (Supplementary Fig. 20). The LUMOs of both compounds extend onto the phenyl or pyrazine ring, leading to a narrower HOMO-LUMO energy gap (2.82 eV for **DDCzBPh** and 2.79 eV for **DDCzBPy**) than the parent **DtBuCzB** (3.37 eV). For **DDCzBPy**, the HOMO/LUMO energy levels are −5.03/−2.24 eV, both of which are slightly more stabilized than those of **DDCzBPh** (−4.98/−2.15 eV), attributed to the presence of the π-accepting pyrazine motif.

The excited-state energies and Δ*E*_ST_ were calculated at the SCS-ADC2/cc-pVDZ level, as we have previously demonstrated that this level of theory is sufficient to accurately predict Δ*E*_*ST*_^27,28^. The difference density plots for the S_1_ and T_1_ excited states are shown in Supplementary Fig. 21. The alternating pattern of increasing and decreasing electron density of the **DtBuCzB** units in the excited S_1_ and T_1_ states for both compounds reveals that these excited states possess SRCT character. The electron density on the naphthalene or quinoxaline fragments is increased upon the transition between ground state to excited S_1_ and T_1_ states, indicating that these excited states in both emitters also possess a certain degree of long-range charge transfer (LRCT) character. Both **DDCzBPh** and **DDCzBPy** exhibit identical S_1_ energies (2.22 eV) and similar T_1_ levels (2.13 and 2.11 eV), resulting in moderate Δ*E*_*ST*_ values of 0.09 and 0.11 eV, respectively. In contrast, **DtBuCzB** has much higher S_1_ and T_1_ levels at 2.87 and 2.75 eV, respectively, while maintaining a similar magnitude Δ*E*_*ST*_ of 0.13 eV^29^. The comparable Δ*E*_*ST*_ values and conserved SRCT character in both the S_1_ and T_1_ states indicate that the designed molecules retain the desired MR-TADF characteristics of the parent **DtBuCzB**. The spin-orbit coupling matrix elements (SOCME) between low-lying excited states were calculated based on the optimized T_1_ geometry at the PBE0/6-31 G(d,p) level (Supplementary Fig. 23). The S_1_ and T_1_ of both compounds have similar orbital character, resulting in small SOCME values ${H}_{{SOC}}^{S1T1}$, of 0.04 cm^−1^ for **DDCzBPh** and 0.10 cm^−1^ for **DDCzBPy**. The closely low-lying T_2_ state to S_1_ has distinct orbital character to S_1_, leading to larger ${H}_{{SOC}}^{S1T2}$ of 0.67 and 0.60 cm^−1^ for **DCzBPh** and **DCzBPy**, respectively. Thus, it is likely that the RISC process involves participation from this state in both compounds.

The *g*_*PL*_ value were calculated following the simplified formula *g* = 4cos*θ*×|${{\bf{m}}}$ | /|${{\boldsymbol{\mu }}}$ | , where ${{\bf{m}}}$ and ${{\boldsymbol{\mu }}}$ are the magnetic and electronic transition dipole moments, and *θ* is the angle between these two, obtained from the time-dependent DFT (TD-DFT) calculations based on the optimized S_1_ geometry at the B3LYP/def2-SVP level with an implicit toluene solvent field (IEFPCM). The calculated |*g*_*PL*_| values are −7.2 × 10^−3^ and −7.1 × 10^−3^ for **DDCzBPh** and **DDCzBPy**, respectively.

### Optoelectronic properties

**Fig. 3:**
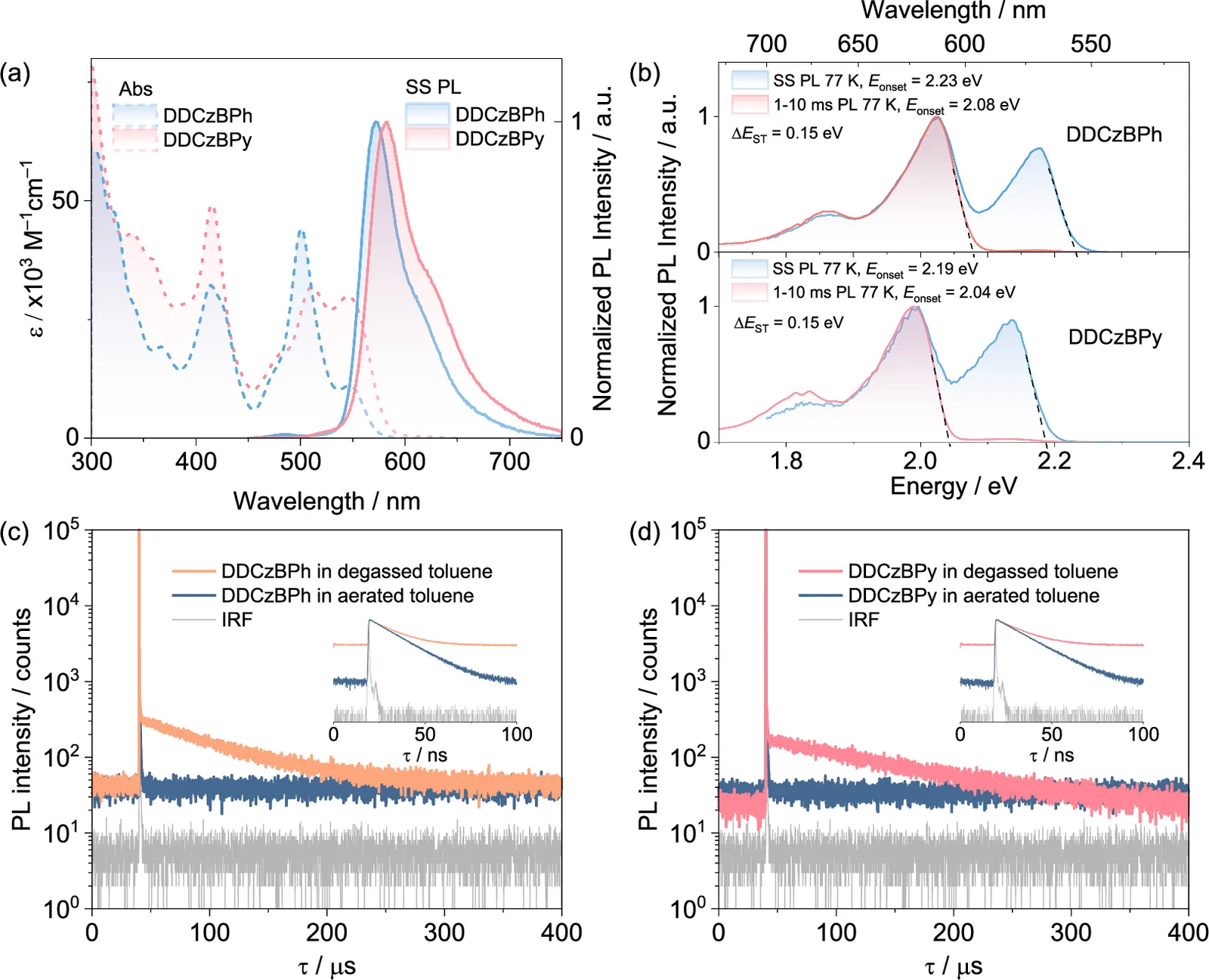
Photophysical properties of DDCzBPh and DDCzBPy. **a** UV-vis absorption and steady-state (SS) PL spectra recorded in aerated toluene at 298 K (*λ*_*exc*_ = 340 nm). **b** SS PL spectra at 77 K (*λ*_*exc*_ = 340 nm), and phosphorescence spectra (1–10 ms, *λ*_*exc*_ = 340 nm) of **DDCzBPh** and **DDCzBPy** in methylcyclohexane at 77 K. Time-resolved PL decays (*λ*_*exc*_ = 375 nm) of **c** **DDCzBPh** and **d** **DDCzBPy** in toluene in air and under vacuum (inset figures are the time-resolved PL decays of the prompt component, *λ*_*exc*_ = 375 nm, time range is 0–100 ns) at room temperature. Concentration of 1 × 10^-5 ^M.

We began our photophysical study by measuring the absorption and photoluminescence (PL) spectra of the racemates in toluene. The UV-vis absorption spectrum of **DDCzBPh** (Fig. 3a) shows an intense absorption band peaking at *λ*_*abs*_ of 501 nm (molar absorptivity coefficient, *ε*, of 44.09 × 10^3 ^M^-1^ cm^-1^) and a lower intensity shoulder band at *λ*_*abs*_ of 546 nm (*ε* of 10.99 × 10^3 ^M^-1^ cm^-1^). The absorption spectrum of **DDCzBPy** is slightly bathochromically shifted, with corresponding bands peaking at *λ*_*abs*_ of 511 nm (*ε* of 31.99 × 10^3 ^M^-1^ cm^-1^) and *λ*_*abs*_ of 546 nm (*ε* of 29.92 × 10^3 ^M^-1^ cm^-1^). The lowest-energy absorption bands of both emitters are ascribed to SRCT transitions to their S_1_ states, whereas the higher-energy bands are attributed to the transitions to the S_2_ states with SRCT and slightly LRCT between **DtBuCzB** fragments and naphthalene or quinoxaline for **DDCzBPh** and **DDCzBPy**, respectively. The simulated absorption spectra match well with the experimental ones (Supplementary Fig. 22). In contrast, the absorption spectrum of **DtBuCzB** shows only one sharp absorption band at around 470 nm (*ε* of around 30 × 10^3 ^M^-1^ cm^-1^), which corresponds to the SRCT transition. The HOMO/LUMO energy levels are estimated as −5.04/−2.69 eV and −5.11/-2.83 eV for **DDCzBPh** and **DDCzBPy**, respectively, calculated from their reversible oxidation and reduction potentials by cyclic voltammetry (CV) and differential pulse voltammetry (DPV) in deaerated DCM (Supplementary Fig. 24, Supplementary Table 3).

The unstructured PL spectra in toluene for both compounds are almost identical. The PL spectrum of **DDCzBPh** peaks at *λ*_*PL*_ at 572 nm (Table 1) and is moderately broad, with a FWHM of 0.17 eV (47 nm), while the PL spectrum of **DDCzBPy** is slightly bathochromically shifted and broadened at *λ*_*PL*_ at 583 nm (FWHM of 0.22 eV, 62 nm, Table 1). Solvatochromic PL studies for **DDCzBPh** reveal a small degree of positive solvatochromism that is consistent with its excited state being of SRCT character (Supplementary Fig. 25). There is relatively stronger positive solvatochromic behavior for **DDCzBPy**, which can be attributed to the relatively greater LRCT character of the emissive excited state promoted by the presence of the more strongly electron-withdrawing quinoxaline bridge. The S_1_ and T_1_ energies were determined from the onsets of the steady-state PL and phosphorescence spectra, respectively, in methylcyclohexane glass at 77 K (Fig. 3b). **DDCzBPy** possesses more stabilized S_1_/T_1_ states (2.19/2.04 eV) than those of **DDCzBPh** (2.23/2.08 eV); however, this nonetheless translates into both compounds having the same Δ*E*_*ST*_ of 0.15 eV. The trend in excited-state energies aligns well with the SCS-ADC2 calculations. This measured Δ*E*_*ST*_ is the same value as that previously reported for **DDCzBPh**^13^. The moderate Δ*E*_*ST*_ is sufficiently small that these two compounds should emit TADF.

**Table 1:** Photophysical properties of DDCzBPh and DDCzBPy

Compound	*λ*_*abs*_^a^ / nm	*λ*_*PL*_^a^ / nm	*FWHM*^a^ / nm	*Φ*_*PL*_^a^ / %	*τ*_*p*_^a,b^ / ns	*τ*_*d*_^a,b^ /μs	S_1_/T_1_^a, b^ / eV	*ΔE*_*ST*_^c, d^ / eV
DDCzBPh	414, 501, 546	572	47	65 (33)	11.2	65.0	2.23/2.08	0.15
DDCzBPy	415, 511, 546	583	62	80 (40)	11.8	109.5	2.19/2.04	0.15

^a^In toluene at 298 K, values in parentheses are *Φ*_*PL*_ measured in aerated toluene.

^b^Prompt and delayed emission were measured by TCSPC (*λ*_*exc*_ = 375 nm) and MCS (*λ*_*exc*_ = 375 nm), respectively.

^c^Measured in glassy methylcyclohexane at 77 K.

^d^Determined from the difference in the onsets of the steady-state PL and phosphorescence spectra at 77 K.

The *Φ*_*PL*_ of **DDCzBPh** and **DDCzBPy** measured in degassed toluene solutions are 65 and 80% (Table 1), respectively. The PL is sensitive to O_2_ and the *Φ*_*PL*_ values decrease dramatically to 33 and 40% in air. The time-resolved PL behavior of both molecules in degassed toluene show biexponential decay kinetics, with prompt and delayed PL lifetimes, *τ*_*p*_ and *τ*_*d*_, of 11.2 ns and 65.0 μs for **DDCzBPh**, 11.8 ns and 109.5 μs for **DDCzBPy** (Fig. 3c, d, Table 1). The longer delayed lifetime for **DDCzBPy** is correlated with the slightly smaller ${H}_{{SOC}}^{S1T2}$ of 0.60 cm^-1^ than that calculated for **DDCzBPh** (0.67 cm^-1^) (Supplementary Fig. 23). The values for **DDCzBPh** are nearly identical to those previously reported (*τ*_*p*_ of 8.4 ns and *τ*_*d*_ of 63 μs)^13^, while for **DtBuCzB**, only prompt emission was detected in degassed toluene, having a *τ*_*p*_ of 5.0 ns^13,29^. Based on the *τ*_*d*_, the estimated *k*_*RISC*_ in toluene is 2.51×10^4 ^s^-1^ for **DDCzBPh** and 1.45×10^4 ^s^-1^ for **DDCzBPy** (see Supplementary Table 4 for full kinetics parameters for both compounds in toluene).

### Chiroptical properties

**Fig. 4:**
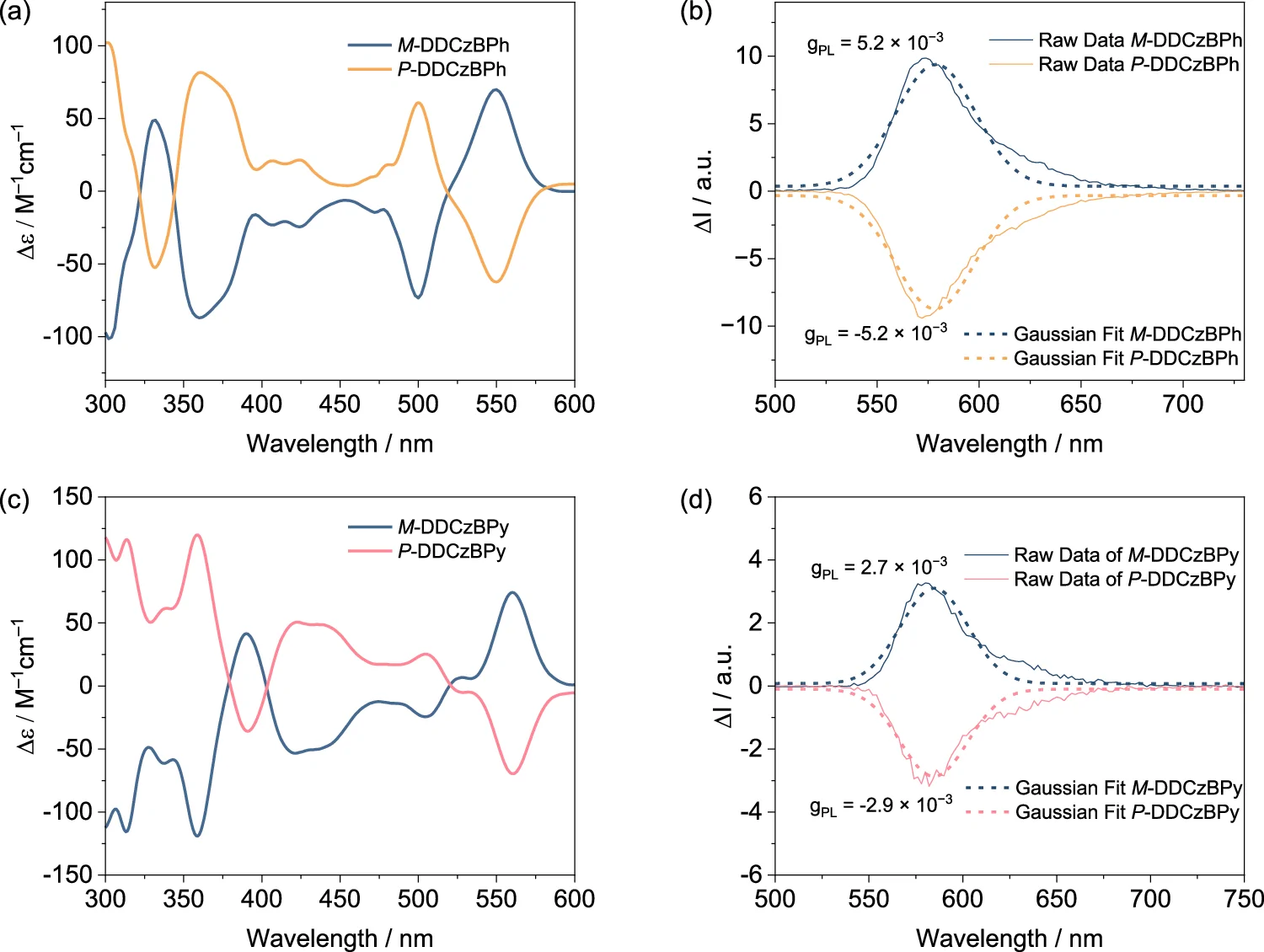
Experimental CD and 1PE-CPL spectra. CD spectra of (**a**) **DDCzBPh** and (**c**) **DDCzBPy** in toluene. 1PE-CPL spectra of (**b**) **DDCzBPh** and (**d**) **DDCzBPy** in toluene at 298 K (*λ*_*exc*_ = 416 nm, dashed line is the Gaussian fit).

**Fig. 5:**
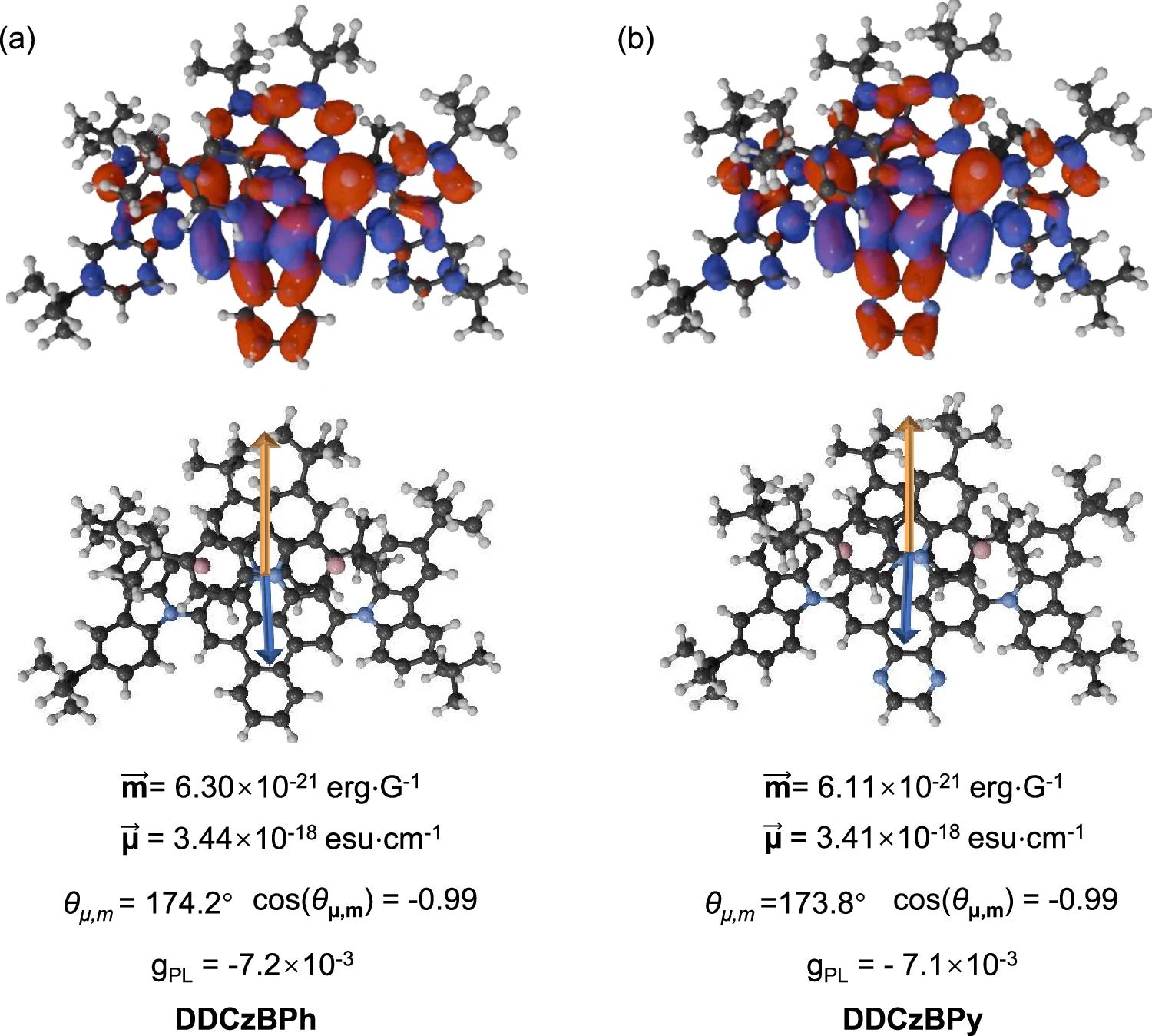
Theoretical calculated chiroptical properties. NTOs (particle and hole are represented by red and blue colors, respectively) and theoretical predicted electronic (yellow arrow), magnetic (blue arrow) transition dipole moment and their angles during the S_1_ to S_0_ transition processes, and predicted *g*_*PL*_ values based on the optimized S_1_ geometries at the B3LYP/def2-SVP level in the toluene for **a** **DDCzBPh** and **b** **DDCzBPy**.

The CD spectra of both enantiomers of **DDCzBPh** and **DDCzBPy** recorded in toluene are shown in Fig. 4a and c, respectively. The spectra of the enantiomers are near mirror images. The signal between 520 to 600 nm is associated mainly with the SRCT S_0_-S_1_ transition. The maximum absorption dissymmetry factors *g*_*abs,max*_ values were observed at around 558 nm and calculated to be −6.14 × 10^-3^ and +6.91 × 10^-3^ for ***P*-DDCzBPh** and ***M*-DDCzBPh**, slightly higher than the previously reported *g*_*abs*_ values of −5.6 × 10^3^ and +5.5 × 10^3^^13^. The *g*_*abs,max*_ values are −6.32 × 10^-3^ and +6.25 × 10^-3^ for ***P*-DDCzBPy** and ***M*-DDCzBPy** at *λ*_*abs*_ of 573 nm. Upon excitation at 416 nm, the enantiomers of both molecules emit mirror-like one-photon excitation (1PE) CPL (ΔI = I_L_ - I_R_, Fig. 4b, d). The *g*_*PL*_ values are −5.2 × 10^-3^ and +5.2 × 10^-3^ for ***P*-DDCzBPh** and ***M*-DDCzBPh** (Supplementary Fig. 26), which are similar to those previously reported of −5.5 × 10^3^ and +5.8 × 10^3^, respectively^13^. The g_PL_ values for ***P*-DDCzBPy** and ***M*-DDCzBPy** are again lower at −2.9 × 10^-3^ and +2.7 × 10^-3^. These g_PL_ values are not dissimilar in magnitude to those of other CP-MR-TADF emitters^9,18^, and are of the same magnitude as the DFT calculated results (calculated |*g*_*PL*_| values of −7.2 × 10^-3^ and −7.1 × 10^-3^ for **DDCzBPh** and **DDCzBPy**, respectively). The NTO analysis indicates that the S_1_ states of both emitters are predominantly SRCT in nature, with only limited intramolecular long-range charge-transfer (LRCT) character between the naphthyl/quinoxanyl unit and the **DtBuCzB** backbone. Furthermore, both emitters exhibit similar, approximately C_2_-symmetric transition patterns rather than pronounced symmetry breaking (Fig. 5). For **DDCzBPy**, although the S_1_ state is still predominantly SRCT in nature, its larger charge transfer distance (*D*_*CT*_ = 0.679 Å for **DDCzBPy** and *D*_*CT*_ = 0.270 Å for **DDCzBPh**) may account for the noticeably lower experimental *g*_*PL*_ than the calculated value^30^. The 1PE CPL brightness values are calculated to be 74.51 and 74.51 M^-1^ cm^-1^ for ***P/M*-DDCzBPh** and 34.71 and 32.31 M^-1^ cm^-1^ for ***P/M*-DDCzBPy**, respectively. The measured 1PE CPB for ***P/M*-DDCzBPh** are smaller than the reported values of 209.7/220.75 M^-1^ cm^-1^^13^. The detailed calculated parameters are summarized in Supplementary Table 2 and key chiroptical properties are listed in Supplementary Table 5.

**Fig. 6:**
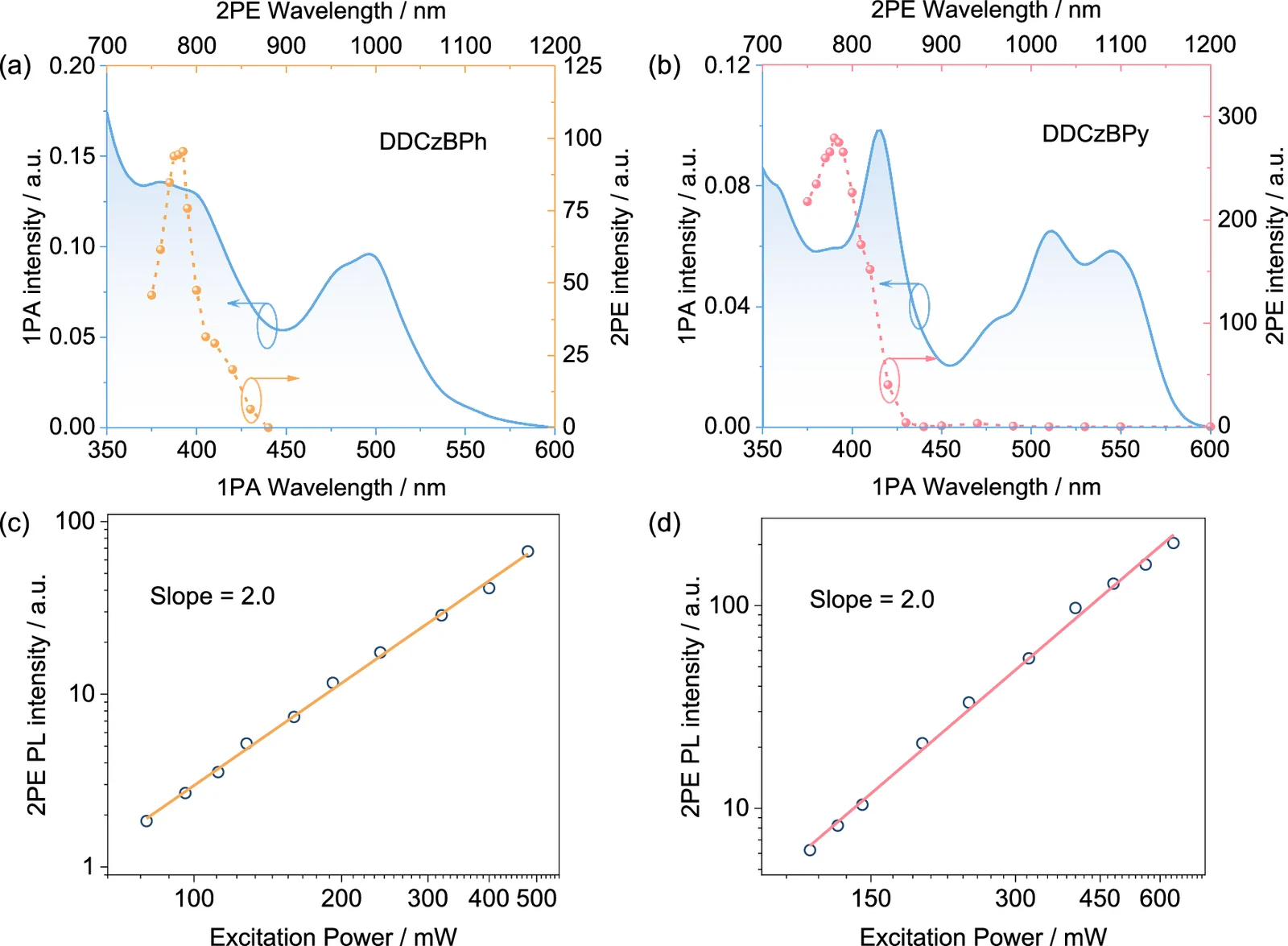
One-photon absorption and two-photon excitation properties. One-photon absorption (solid line) and two-photon excitation (dotted line) spectra of **a** **DDCzBPh** (*λ*_*em*_ = 550-650 nm) and **b** **DDCzBPy** (*λ*_*em*_ = 550–650 nm). Log-log plot of the excitation-power dependence (blue dots) of the 2PE-induced photoluminescence intensity for **c** **DDCzBPh**, slope is 2.0 and **d** **DDCzBPy**, slope is 2.0.

Both emitters show strong potential for two-photon absorption due to their extended π-conjugation, which is known to enhance the 2PA cross-section^17^. These two designed emitters are expected to show larger 2PA *σ*^*2*^ than the weak reported 2PA activity of their parent MR-TADF compound, **CzBN**^19^. To validate these predictions, the 2PA properties of **DDCzBPh** and **DDCzBPy** were investigated (Fig. 6a, b). The maximum PL intensity was obtained at a 2PE of 785 nm (1.58 eV) for **DDCzBPh** and 780 nm (1.59 eV) for **DDCzBPy**, which is lower than the double of the one-photon absorption maximum wavelength (501 nm, 2.48 eV for **DDCzBPh** and 500 nm, 2.43 eV for **DDCzBPy**). This is due to the nonlinear effect of non-degenerate two-photon absorption^6^. Two-photon excitation processes were confirmed from the logarithmic dependence of the 2PE intensity with laser excitation power (Fig. 6c, d). The 2PA cross-sections are 118 GM at 780 nm and 142 GM at 780 nm for **DDCzBPh** and **DDCzBPy**, respectively. These values are slightly higher than those reported for the small number of other 2PA-TADF emitters (Supplementary Fig. 2).

**Fig. 7:**
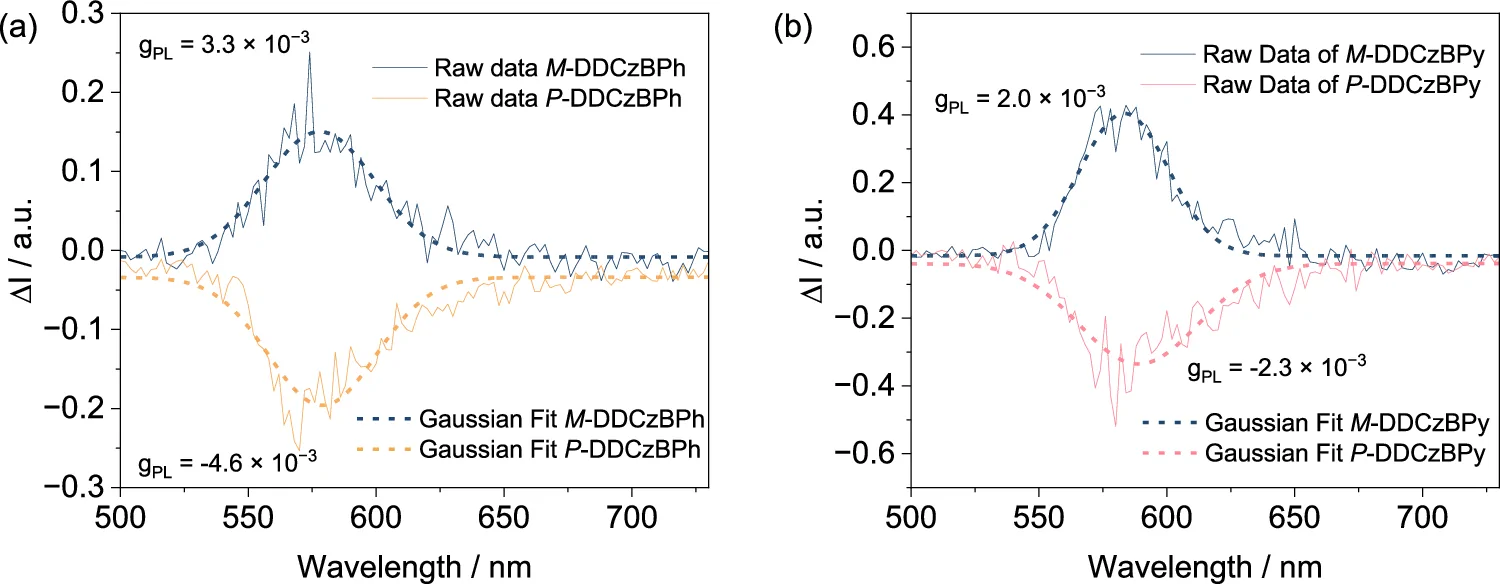
2PE-CPL spectra. **a** **DDCzBPh** (*λ*_*exc*_ = 780 nm) and **b** **DDCzBPy** (*λ*_*exc*_ = 780 nm) measured in toluene at room temperature.

With both compounds showing promising chiroptical and 2PA properties, we next explored their 2PE-CPL properties to assess their potential as 2PE-CPL probes in bioimaging applications. Upon excitation of each emitter by a pulsed laser at 780 nm, 2PE-CPL spectra were recorded using the same CPL spectrometer as the 1PE-CPL spectra (ΔI = I_L_ - I_R_, Fig. 7). The CPL spectra of the enantiomers of each of the emitters show similar mirror-like behavior as their 1PE-CPL spectra (Fig. 4b, d). The *g*_*PL*_ values of -4.6×10^-3^ and +3.3×10^-3^ for ***P*-**/***M*-DDCzBPh**, and −2.3 × 10^-3^ and +2.0 × 10^-3^ for ***P*-**/***M*-DDCzBPy** were measured by two-photon excitation, and were of similar magnitude to their respective values measured by one-photon excitation (−5.2 × 10^-3^ and +5.2 × 10^-3^ for ***P*-**/***M*-DDCzBPh**, −2.9 × 10^-3^ and +2.7 × 10^-3^ for ***P*-**/***M*-DDCzBPy**) (Supplementary Fig. 27). The calculated 2PE-CPB values are 0.20 and 0.20 GM for ***P-/M-*DDCzBPh** and 0.16 and 0.15 GM for ***P-/M*-DDCzBPy** from their most intense CPL transitions at around 578 nm and 587 nm, respectively (Supplementary Table 6). These 2PE-CPB values are larger than the known example of diaza[4]helicenes (2PE-CPB value of 0.05 GM)^23^, and comparable to recently reported chiral Eu complexes (0.16/0.17 GM)^6^. Notably, the extended π-conjugative framework within the helicene boosts 2PA independently of chiroptical effects, raising *σ*^*2*^ from 35 GM at 740 nm in the parent compound **CzBN** to more than 100 GM at 780 nm in the current emitters. Furthermore, the presence of the embedded nitrogen atoms increases the π-accepting character of the quinoxanyl group, strengthening the intramolecular charge transfer (ICT) character of the emissive excited state of **DDCzBPy**, which was confirmed by a combination of a more pronounced positive PL solvatochromism and larger charge transfer distance (Supplementary Figs. 20 and 25). The stronger ICT character contributes to increasing *σ*^*2*^ to 142 GM for **DDCzBPy**, compared with 118 GM for **DDCzBPh**. However, by introducing ICT character and decreasing the symmetry of the molecule, the parallel alignment between the electric and magnetic transition dipoles is disrupted, yielding smaller *g*_*PL*_ values, consistent with both experimental measurements and theoretical calculations. Although these *g*_*PL*_ values are smaller than chiral Eu(III) complexes (|*g*_*PL*_ | > 0.01)^6^, the present emitters remain promising for CPL bioimaging. This is supported by a prior report in which **(*S*)-BOD1**, with a small |*g*_*PL*_| of 6 × 10^-4^ and CPB of 12.3 M^–1^ cm^–1^, was used as a CPL probe in living cells using CPL-laser scanning confocal microscopy (CPL-LSCM)^31^.

## Discussion

Our study reports chiral MR-TADF emitters that generate substantial 2PE-CPL. The materials design relies on bridging two MR-TADF **DtBuCzB** motifs via either a naphthalene or quinoxaline unit to form a helical structure. The CPL is relatively strong, with 1PE *g*_*PL*_ values of -5.2/ + 5.2 × 10^-3^ for ***P*-/*M*-DDCzBPh** and −2.9/ + 2.7 × 10^-3^ for ***P*-/*M*-DDCzBPy**, high *Φ*_*PL*_ of 65 and 80%, long delayed lifetimes of 65 and 109.5 μs, and large 2PA *σ*^*2*^ of 118 and 142 GM for **DDCzBPh** and **DDCzBPy**, respectively. The 2PE-CPL spectra show comparable *g*_*PL*_ values of −4.6/ + 3.3 × 10^−3^ for ***P-/M*-DDCzBPh** and −2.3/ + 2.0 × 10^−3^ for ***P-/M*-DDCzBPy**, associated with 2PE-CPB values of 0.20/0.20 GM and 0.16/0.15 GM, respectively. The outstanding chiroptical and photophysical properties originate from the extended helicity and rigid π-conjugation of these structures, as well as the short-range charge transfer excited states of these MR-TADF emitters. This work marks a significant advancement toward chiral systems with enhanced 2PA properties and delayed luminescence.

## Methods

### General synthetic procedures

**DtBuCzB-Bpin**^32^ was synthesized according to a literature procedure (Supplementary Fig. 3). All other reagents and solvents were obtained from commercial sources and used as received. Air-sensitive reactions were performed under a nitrogen atmosphere using Schlenk techniques. Dry solvents used in the reactions were obtained from an MBRAUN SPS5 solvent purification system. Flash column chromatography was carried out using silica gel (Silia-P from Silicycle, 60 Å, 40-63 µm). Analytical thin-layer chromatography (TLC) was performed with silica plates with aluminum backings (250 µm with F-254 indicator). TLC visualization was accomplished by a 254/365 nm UV lamp. Analysis and separation of enantiomers were conducted on a Shimadzu LC-40 HPLC and FRC system. HPLC were performed using a Daicel 86335 CHIRALPAK IF (250 × 10 mm, 5 µm particle size) column with HPLC-grade dichloromethane/hexane as the mobile phase. GCMS analysis was conducted using a Shimadzu QP2010SE GC-MS equipped with a Shimadzu SH-Rtx-1 column (30 m × 0.25 mm). ^1^H and ^13^C NMR spectra were recorded on a Bruker Advance spectrometer (500 MHz for ^1^H, 126 MHz for ^13^C) in CDCl_3_ or CD_2_Cl_2_. The following abbreviations have been used for multiplicity assignments: “s” for singlet, “d” for doublet, “dd” for doublet of doublets, “m” for multiplet. ^1^H and ^13^C NMR spectra were referenced to residual solvent peaks. Melting points were measured using open-ended capillaries on an Electrothermal 1101D Mel-Temp apparatus and are uncorrected. High-resolution mass spectrometry (HRMS) was performed at the University of Edinburgh Mass Spectrometry Facility on a micrOTOF, and elemental analyses (EA) was performed at the London Metropolitan University by O. McCullough.

### Electrochemistry measurements

Cyclic Voltammetry (CV) analyses were performed on an Electrochemical Analyzer potentiostat model 620E from CH Instruments at a sweep rate of 100 mV/s. Differential Pulse Voltammetry (DPV) analyses were carried out with an increment potential of 0.004 V and a pulse amplitude, width, and period of 50 mV, 0.06 s, and 0.5 s, respectively. Samples were prepared in DCM and degassed by sparging with DCM-saturated nitrogen gas for 5 minutes before measurements. All measurements were performed in 0.1 M DCM solution of tetra-*n*-butylammonium hexafluorophosphate, [^*n*^Bu_4_N]PF_6_, which was used as the supporting electrolyte. An Ag/Ag^+^ electrode was used as the reference electrode, while a carbon electrode and a platinum wire were used as the working electrode and counter electrode, respectively. The redox potentials are reported relative to a saturated calomel electrode (SCE) with a ferrocene/ferrocenium (Fc/Fc^+^) redox couple as the internal standard (0.46 V vs SCE for DCM)^33^. The HOMO and LUMO energies were determined using the relation E_HOMO/LUMO_ = −(E_ox_ / E_red_ + 4.8) eV, where E_ox_ and E_red_ are the onset of anodic and cathodic peak potentials, respectively calculated from DPV relative to Fc/Fc^+^^34^.

### X-ray crystallography

X-ray diffraction data for compounds **DDCzBPh** and **DDCzBPy** were collected using a Rigaku MM-007HF High Brilliance RA generator/confocal optics with either XtaLAB P100 or P200 diffractometers [Cu Kα radiation (*λ* = 1.54187 Å)]. Data for both compounds were collected (using a calculated strategy) and processed (including correction for Lorentz, polarization, and absorption) using CrysAlisPro^35^. Structures were solved by dual-space methods (SHELXT^36^) and refined by full-matrix least-squares against F^2^ (SHELXL-2019/3^37^). Non-hydrogen atoms were refined anisotropically, and hydrogen atoms were refined using a riding model. Multiple *tert*-butyl groups in both structures showed rotational disorder (with one showing additional rocking out of the plane of the adjacent ring structure) and were each modelled in two parts with appropriate thermal and geometric restraints. Some disordered *tert*-butyl groups were not split due to the low occupancy of the minor part and instead were treated with thermal restraints. In the structure of **DDCzBPh**, two of the toluene solvates showed positional disorder and were each modelled in two parts with the minor part placed using FragmentDB^38,39^ and refined with appropriate thermal and geometric restraints. The structure of **DDCzBPy** showed high proportions of void space (1268 Å^3^, 14%) and the SQUEEZE^40^ routine implemented in PLATON^41^ was used to remove the contribution to the diffraction pattern of the unordered electron density in the void spaces. All calculations except SQUEEZE were performed using the Olex2^42^ interface. Selected crystallographic data are presented in Supplementary Table 1. CCDC 2474402 and 2474403 contain the supplementary crystallographic data for this paper. These data can be obtained free of charge from The Cambridge Crystallographic Data Centre via www.ccdc.cam.ac.uk/structures.

### Fitting of the time-resolved luminescence measurements

Time-resolved PL measurements were fitted to a sum of exponential decay model, with chi-squared (*χ*^*2*^) values between 1 and 1.5, using the Fluoracle software (Edinburgh Instruments, UK). Each component of the decay is assigned a weight, (*w*_*i*_), which is the contribution of the emission from each component to the total emission. The average lifetime was then calculated using the following:

For two exponential decay model:

1 $${\tau }_{{AVG}}={\tau }_{1}{w}_{1}+{\tau }_{2}{w}_{2}$$

with weights defined as ${w}_{1}$=$\frac{{A}_{1}{\tau }_{1}}{{A}_{1}{\tau }_{1}+{A}_{2}{\tau }_{2}}$ and ${w}_{2}$=$\frac{{A}_{2}{\tau }_{2}}{{A}_{1}{\tau }_{1}+{A}_{2}{\tau }_{2}}$, where *A*_*1*_ and *A*_*2*_ are the preexponential-factors of each component.

For three exponential decay model:

2 $${\tau }_{{AVG}}={\tau }_{1}{w}_{1}+{\tau }_{2}{w}_{2}+{\tau }_{3}\,{w}_{3}$$

with weights defined as ${w}_{1}$=$\frac{{A}_{1}{\tau }_{1}}{{A}_{1}{\tau }_{1}+{A}_{2}{\tau }_{2}+{A}_{3}{\tau }_{3}}$, ${w}_{2}$=$\frac{{A}_{2}{\tau }_{2}}{{A}_{1}{\tau }_{1}+{A}_{2}{\tau }_{2}+{A}_{3}{\tau }_{3}}$ and ${w}_{3}$=$\frac{{A}_{3}{\tau }_{3}}{{A}_{1}{\tau }_{1}+{A}_{2}{\tau }_{2}+{A}_{3}{\tau }_{3}}$, where *A*_*1*_, *A*_*2*_ and *A*_*3*_ are the preexponential-factors of each component.

### Theoretical calculations

Density functional theory (DFT) and TD-DFT calculations were performed using the Gaussian 16 revision C.01 package^43^. The ground-state optimizations and vibrational frequency calculations were carried out using DFT using the CAM-B3LYP^44^ functional and the 6-31 G(2 d,p) basis set^45^ in toluene with the PCM model, starting from a crystal structure. The vertical excited-state calculations were performed using time-dependent DFT (TD-DFT)^46^ using the B3LYP functional and the def2-SVP basis set in toluene. Spin-orbit coupling matrix elements (SOCME) were calculated based on the optimized triplet excited-state geometry at the PBE0/6-31 G(d,p) level in the gas phase using PySOC^47^. The molecular orbital distributions were visualized with Gaussview 6.0^48^. Wavefunction calculations were performed using the Turbomole 7.5 package^49,50^ using the resolution of the identity approximation^51^. The spin-component scaling (SCS) modification was applied to all calculations^52^. Vertical excited state calculations using SCS-ADC(2)^53,54^ with the cc-pVDZ basis set^55^ were performed on the DFT optimized ground state geometry, with the first singlet state^56^ and first triplet state^57,58^ calculated. Calculations were submitted and processed using the Digichem software package (version 6)^59,60^, which incorporates a number of publicly available software libraries, including: cclib^61^ for parsing of result files, VMD^62^/Tachyon^63^ for 3D rendering, Matplotlib^64^ for drawing of graphs, Open Babel^65^/Pybe^66^ file interconversion.

### Photophysical measurements

Optically dilute solutions of concentrations on the order of 10^−5^ or 10^−6^ M were prepared in spectroscopic or HPLC-grade solvents for absorption and emission analysis. Absorption spectra were recorded at room temperature on a Shimadzu UV-2600 double-beam spectrophotometer with a 1 cm quartz cuvette. Molar absorptivity determination was verified by a linear regression analysis of values obtained from five independent solutions at varying concentrations with absorbance ranging from 0.237 to 0.842 for **DDCzBPh** and from 0.279 to 0.768 for **DDCzBPy**.

For emission studies, aerated solutions were sparged with compressed air for 5 min, degassed solutions were prepared via three freeze-pump-thaw cycles, and spectra were measured using a homemade Schlenk quartz cuvette. Steady-state emission, excitation spectra, and time-resolved emission spectra were recorded at 298 K using an Edinburgh Instruments FS5 fluorimeter. Samples were excited at 340 nm for steady-state PL measurements in toluene and the solvatochromism study using a 150 W xenon lamp. Phosphorescence spectra were collected using a 5 W pulsed xenon microsecond flash lamp by multi-channel scaling (MCS) mode, with a gate time of 1-10 ms. Time-resolved PL decays were carried out using time-correlated single-photon counting (TCSPC) mode for short-time range (1 ns-100 ns) and multi-channel scaling (MCS) mode for longer time ranges (1ns-400 μs, 1 ns-800 μs). The samples were excited at 375 nm by a 5 mW EPL-375 picosecond pulsed diode laser.

Photoluminescence quantum yields for solutions were determined using the optically dilute method^67^ in which four sample solutions with absorbances of ca. 0.090, 0.070, 0.045, 0.024 at 350 nm were used for **DDCzBPh** and 0.092, 0.057, 0.031, 0.018 at 350 nm were used for **DDCzBPy**. The Beer-Lambert law was found to be linear over the solution concentrations. For each sample, linearity between absorption and emission intensity was verified through linear regression analysis and additional measurements were acquired until the Pearson regression factor (R^2^) for the linear fit of the data set surpassed 0.9. Individual relative quantum yield values were calculated for each solution, and the values reported represent the gradient value. The equation *Φ*_*s*_ = *Φ*_*r*_(*A*_*r*_*/A*_*s*_)(*I*_*s*_*/I*_*r*_)(*n*_*s*_*/n*_*r*_)^2^ was used to calculate the relative quantum yield of each of the sample, where Φ_r_ is the absolute quantum yield of the reference, *n* is the refractive index of the solvent, *A* is the absorbance at the excitation wavelength, and *I* is the integrated area under the corrected emission curve. The subscripts s and r refer to the sample and reference, respectively. A solution of quinine sulfate (*Φ*_*r*_ = 54.6% in 1 *N* H_2_SO_4_) was used as the external reference^68^.

The singlet-triplet energy gaps (Δ*E*_*ST*_) in methylcyclohexane and films were estimated from the onsets of the steady-state PL and phosphorescence spectra at 77 K. Phosphorescence spectra were measured using the time-gated method with a gate time of 1-10 ms, where the samples were excited at 340 nm by a 5 W microsecond flash lamp.

### Electronic circular dichromism and one-photon excited circularly polarized luminescence

Electronic circular dichromism (ECD) spectra were measured in toluene at 10^-5 ^M using a MOS-500 circular dichroism spectrometer.

One photon excited circularly polarized luminescence (CPL) was measured in toluene at 10^-5 ^M with a home-built (modular) spectrometer^69^. The excitation source was a EQ 99 broad band (200 - 1000 nm) laser-driven light source (Elliot Scientific). The excitation wavelength was selected by feeding the broadband light into an Acton SP-2155 monochromator (Princeton Instruments); the collimated light was focused into the sample cell (1 cm quartz cuvette). Sample PL emission was collected perpendicular to the excitation direction with a lens (*f* = 150 mm). The emission was fed through a photoelastic modulator (PEM) (Hinds Series II/FS42AA) and through a linear sheet polariser (Comar). The light was then focused into a second scanning monochromator (Acton SP2155) and subsequently on to a photomultiplier tube (PMT) (Hamamatsu H10723 series). The detection of the CPL signal was achieved using the field modulation lock-in technique. The electronic signal from the PMT was fed into a lock-in amplifier (Hinds Instruments Signaloc Model 2100). The reference signal for the lock-in detection was provided by the PEM control unit. The monochromators, PEM control unit and lock-in amplifier were interfaced to a desktop PC and controlled by a custom-written LabVIEW graphic user interface. The lock-in amplifier provided two signals, an AC signal corresponding to (I_L_- I_R_) and a DC signal corresponding to (I_L_ + I_R_) after background subtraction. The emission dissymmetry factor was therefore readily obtained from the experimental data, as 2 AC/DC. Spectral calibration of the scanning monochromator was performed using a Hg-Ar calibration lamp (Ocean Optics, HG-2). A correction factor for the wavelength dependence of the detection system was constructed using a calibrated lamp (Ocean Optics, HL-2000). The measured raw data was subsequently corrected using this correction factor. The validation of the CPL detection systems was achieved using light emitting diodes (LEDs) at various emission wavelengths. The LED was mounted in the sample holder and the light from the LED was fed through a broad band polarising filter and π/4 plate (ThorLabs, LPVISE100-A and AQWP05M-600) to generate circularly polarised light. Prior to all measurements, the π /4 plate and a LED were used to set the phase of the lock-in amplifier correctly. The emission spectra were recorded with 2 nm step size and the slits of the detection monochromator were set to a slit width corresponding to a spectral resolution of 2 nm. CPL spectra (as well as total emission spectra) were obtained through an averaging procedure of 10 scans.

### Two-photon spectroscopy and two-photon excited circularly polarized luminescence^4^

Two-photon excitation CPL spectroscopy has been achieved by coupling (beam routing using mirrors, Thor Labs BB1-E03) a tunable femtosecond pulsed laser (680–1300nm, Coherent Discovery TPC, 100fs, 80MHz) to a pre-existing CPL spectrometer, detailed above. Initial proof of concept two-photon spectroscopy has been achieved by perpendicularly mounting an Ocean Optics HR2000Pro (2048-pixel linear CCD Sony ILX5 chip, H3 grating, 350 - 850nm spectral region, coupled via a 74-DA collimating lens) spectrometer as a ‘third arm’ to the Discovery TPC laser. The laser beam was focused onto the centre of the 1 cm path sample holder (Thor labs CVH100) by a dedicated ultrafast laser lens (Edmund Optics 11711, 50 mm focal length). The spectrometer has also been equipped with a perpendicularly mounted 365nm LED (Nichia, 1W) and has been operated using a modified version of the above-mentioned custom time-resolved detection and accumulation algorithm written in LabVIEW 2013 software. To eliminate unwanted artefacts associated with stray light from multiphoton (MP) excitation, each spectrometer has been equipped with a rotating filter wheel (Thor Labs, CFW6) housing an LP420 (Comar Optics, for 365 nm UVLED excitation) and SP650 - SP800, 25 nm spectral intervals (Edmund Optics, for MP excitation) filters. MP(2PE)-PL spectra were obtained using the following parameters: integration time - 10 ms, resolution - 0.25 nm/pixel, accumulation - 3000.

MP-CPL spectra were obtained using the following parameters: slits - 9 nm max, integration time - 1 ms, resolution step - 2 nm, accumulations - 20.

### Cross-section determination

The cross-sections (σ^2^) of the compounds discussed herein are calculated according to established procedures^70^:

3 $${\sigma}_{s}^{2}={\sigma}_{r}^{2}\cdot {\varPhi }_{r}\cdot \frac{{C}_{r}{n}_{s}{F}_{s}(\lambda )}{{\varPhi }_{s}{C}_{s}{n}_{r}{F}_{r}(\lambda )}$$

Where s is sample, *r* is reference, ϕ is the total emission quantum yield of the compound, *C* is the concentration, *n* the refractive index and ${F}_{s}(\lambda )$ and ${F}_{r}(\lambda )$ are the integrated PL spectrum for the sample and reference, respectively. Additionally, we have demonstrated that the excitation process is definitely a two-photon event by recording an excitation power dependence; the resulting line has a slope of two on a logarithmic scale. The cross sections were calculated with reference to rhodamine B in methanol. The MP spectra were recorded in 1 cm path length quartz cuvettes, in air-equilibrated analytical grade acetonitrile at concentrations *ca*. 10^- 5^ M.

In the reaction conditions, RT indicates an ambient temperature of 20–30 °C.

## Supplementary information

Supplementary Information

Transparent Peer Review file

## Source data

Source Data

## Data Availability

The research data generated in this study have been deposited in the University of St Andrews datasets under accession code 10.17630/3b69fc62-063d-4411-8995-43d9cecd75e1 https://doi.org/10.17630/3b69fc62-063d-4411-8995-43d9cecd75e1. The X-ray structural data can be obtained from The Cambridge Crystallographic Data Centre as deposition numbers 2474402 https://doi.org/10.5517/ccdc.csd.cc2p1tf0 and 2474403 https://doi.org/10.5517/ccdc.csd.cc2p1tg1. Source Data are provided with this manuscript. Source data are provided with this paper.
