# Supplementary material for: Two-photon excited circularly polarized luminescence of hetero[9]helicene with multiresonant thermally activated delayed fluorescence
Source: Nat Commun. 2026 Sep 15;17:10814. doi: 10.1038/s41467-026-77554-5 (PMC13669335; doi:10.1038/s41467-026-77554-5)
Supplement: Supplementary file 1 [file 41467_2026_77554_MOESM1_ESM.pdf]

Supplementary Information

Two-Photon Excited Circularly Polarized Luminescence of Hetero[9]helicene with Multiresonant Thermally Activated Delayed Fluorescence

Sen Wu,[1,2,#] Yan Xu,[1,3,#] Dominic J. Black,[4] Aidan P. McKay,[1] David B. Cordes,[1] Robert Pal,[4,*] and Eli Zysman-Colman[1,*]

[1] Organic Semiconductor Centre, EaStCHEM School of Chemistry, University of St Andrews, St Andrews, KY16 9ST, UK,

[2] College of Physics and Optoelectronics, Taiyuan University of Technology, Taiyuan, 030024, P. R. China.

[3] Key Laboratory of Luminescent Materials and Devices, School of Materials Science and Engineering, South China University of Technology, Guangzhou, 510640, P. R. China.

[4] Department of Chemistry, Durham University, South Road, Durham, DH1 3LE, UK.

[#] These authors contributed equally

* Corresponding authors: eli.zysman-colman@st-andrews.ac.uk (E.Z.-C.), robert.pal@durham.ac.uk (R.P.)

Table of Contents

1. Supplementary crystallographic data S3
2. Literature study S4
3. Experimental section S8
4. Theoretical and optoelectronic data S24
5. Kinetics parameters S29
References S32

1. Supplementary crystallographic data

Supplementary Table **1**. Selected crystallographic data.

	**DDCzBPh**	**DDCzBPy**
formula	$C_{112}H_{114}B_2N_4$	$C_{97}H_{98}B_2ClN_6$
formula weight	1537.69	1404.88
temperature [K]	173	100
crystal description	Red needle	Red needle
crystal size [mm^3]	0.21 × 0.02 × 0.01	0.32 × 0.02 × 0.007
space group	$P\bar{1}$	$P\bar{1}$
a [Å]	17.7933(6)	16.6815(3)
b [Å]	17.9911(6)	19.9104(3)
c [Å]	30.1851(9)	27.8500(4)
α [°]	106.927(3)	109.4202(15)
β [°]	98.491(3)	91.4958(14)
γ [°]	99.395(3)	100.6396(15)
volume [Å]^3	8923.2(6)	8535.4(3)
Z	4	4
ρ (calc) [g/cm^3]	1.145	1.093
μ [mm^{-1}]	0.489	0.757
F(000)	3296	2996
reflections collected	157492	164423
independent reflections (R_{int})	31219 (0.1153)	34357 (0.1145)
parameters, restraints	2440, 735	2081, 286
GoF on F^2	1.008	1.056
R_1 [$I > 2\sigma(I)$]s	0.0660	0.0786
wR_2 (all data)	0.1869	0.2410
largest diff. peak/hole [e/Å^3]	0.48/-0.29	0.50/-0.51

2. Literature study

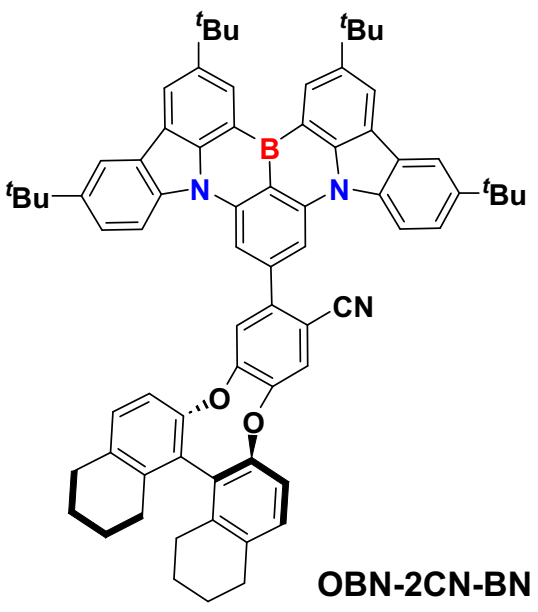

OBN-2CN-BN

$|g_{PL}|$= 9.1 × 10^{-4}

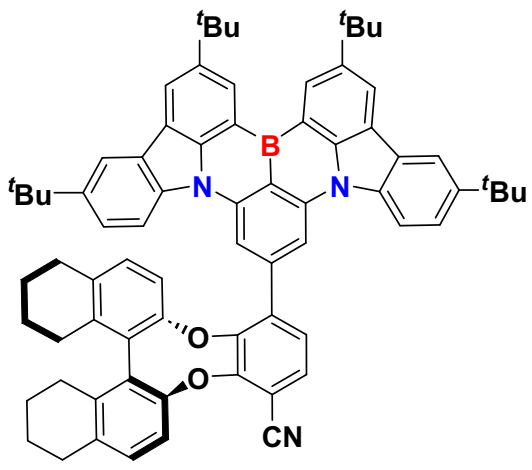

OBN-4CN-BN

$|g_{PL}|$= 10.4 × 10^{-4}

Both in 3 wt% doped films in PhCzBCz

Adv. Mater. **2021**, *33*, 2100652

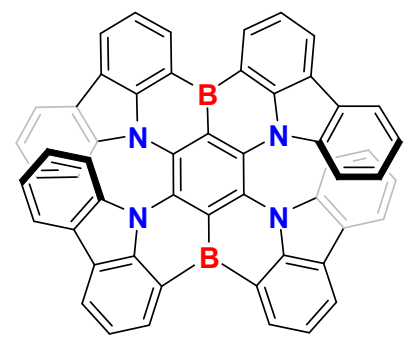

R-BN

$|g_{PL}|$= 2.0 × 10^{-3} in DCM

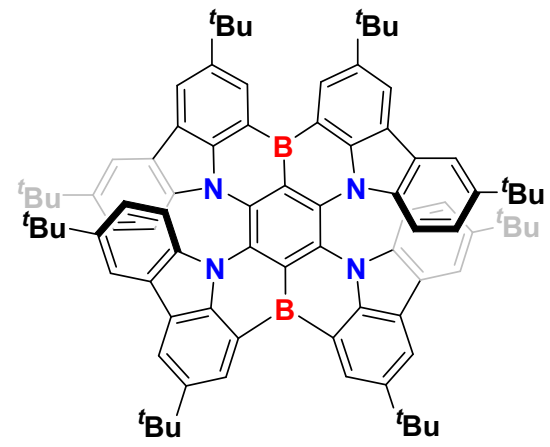

R-TBN

$|g_{PL}|$= 2.0 × 10^{-3} in DCM

Angew. Chem. Int. Ed. **2021**, *60*, 20498

J. Am. Chem. Soc. **2021**, *143*, 17958

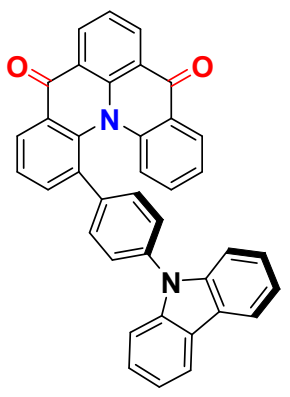

QAO-PhCz

$|g_{PL}|$= 1.1 × 10^{-3} in toluene

Chem. Commun. **2021**, *57*, 11041

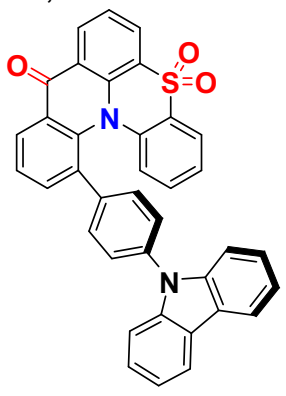

QPO-PhCz

$|g_{PL}|$= 1.6 × 10^{-3} in DCM

J. Mater. Chem. C **2022**, *10*, 4393

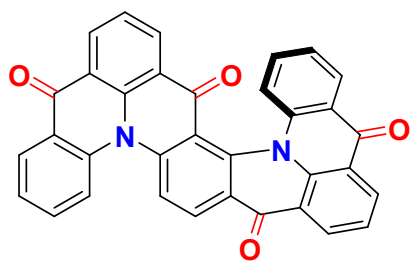

Hel-DiDiKTa

$|g_{PL}|$= 4.0 × 10^{-4} in toluene

J. Mater. Chem. C **2022**, *10*, 4861

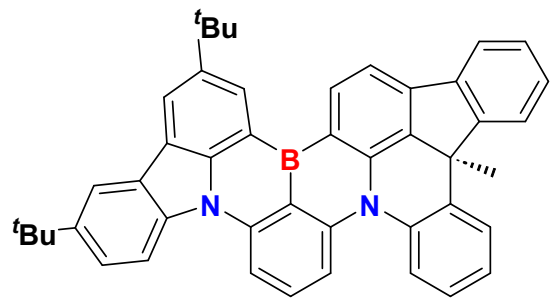

BN-MeIAc

$|g_{PL}|$= 3.2 × 10^{-4}

in 1 wt% doped films in DMIC-TRZ

Angew. Chem. Int. Ed. **2022**, *61*, e202202227

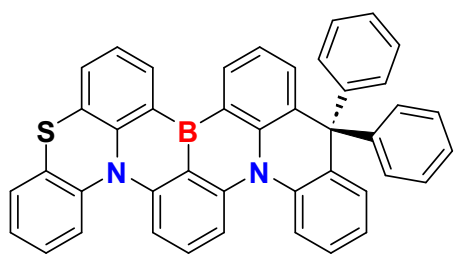

BN_4

$|g_{PL}|$= 1.1 × 10^{-3}

in 3 wt% doped films in mCPCN

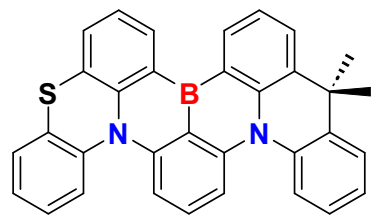

BN_5

$|g_{PL}|$= 1.3 × 10^{-3}

in 1 wt% doped films in mCPCN

Adv. Mater. **2022**, *34*, 2105080

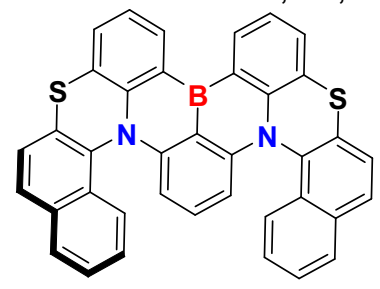

Helicene-BN

$|g_{PL}|$= 2.1 × 10^{-3} in toluene

CCS Chem. **2022**, *4*, 3463

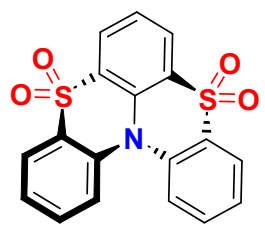

BTPT

$|g_{PL}|$= 2.8 × 10^{-3} in crystal

Sci. China Chem. **2022**, *65*, 1715

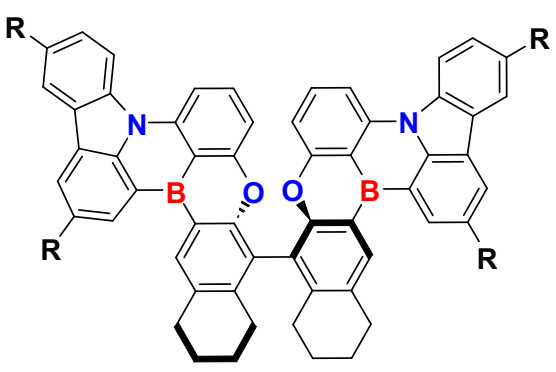

R=H **DOBN** $|g_{PL}|$= 1.0 × 10^{-3}

R=^{t}Bu **DOBNT** $|g_{PL}|$= 0.9 × 10^{-3}

Both in 5 wt% doped films in 2,6-DCzPPy

Adv. Mater. **2022**, *34*, 2204253

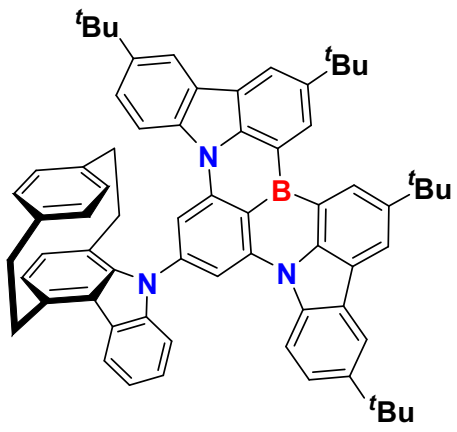

Czp-tBuCzB

$|g_{PL}|$= 1.6 × 10^{-3}

in 5 wt% doped films in 2,6-DCzPPy

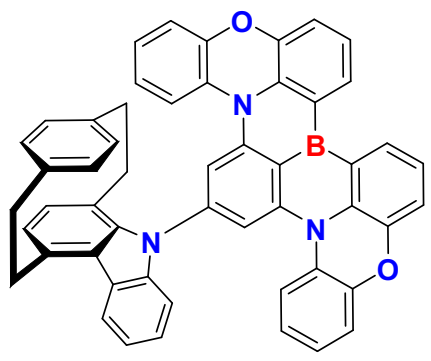

Czp-POAB

$|g_{PL}|$= 1.4 × 10^{-3}

in 8 wt% doped films in 2,6-DCzPPy

Angew. Chem. Int. Ed. **2023**, *62*, e202217045

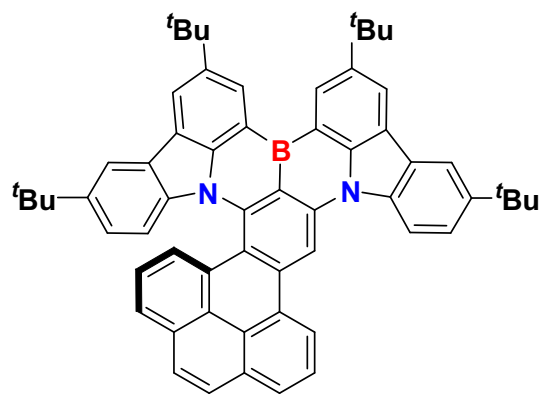

BN-Py

$|g_{PL}|$= 5.2 × 10^{-4} in toluene

Adv. Mater. **2023**, *35*, 2305125

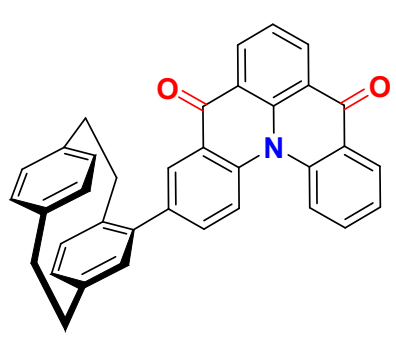

PCP-DiKTa

$|g_{PL}|$ not provided

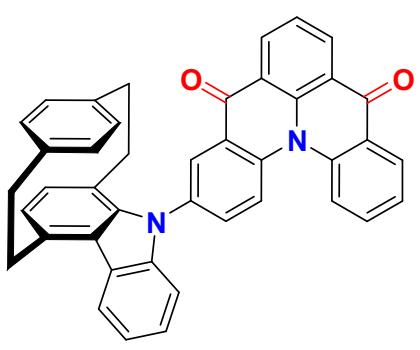

Czp-DiKTa

$|g_{PL}|$= 4 × 10^{-4} in toluene

Adv. Funct. Mater. **2024**, 2402036

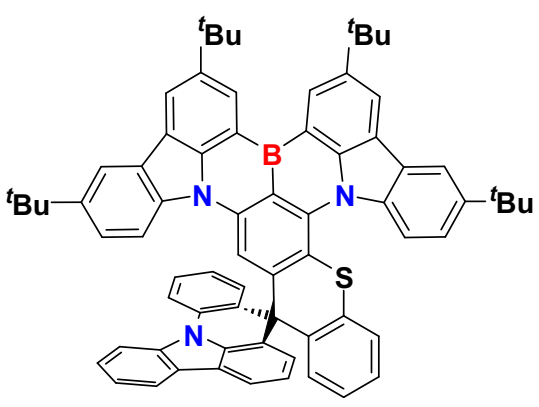

Spiro-BNCz

$|g_{PL}|$= 1.3 × 10^{-3}

in 5 wt% doped films in 2,6-DCzPPy

Aggregate **2024**, *5*, e445

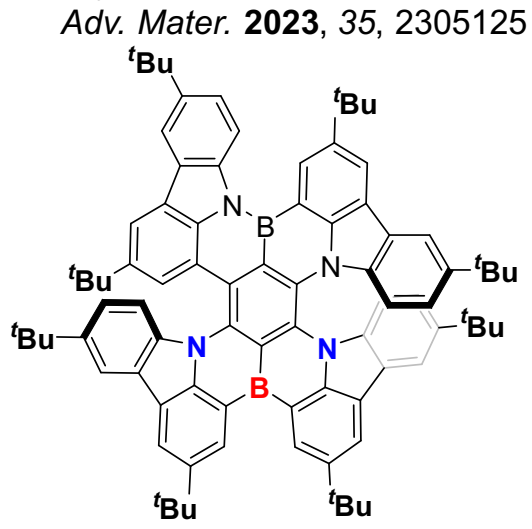

RBNN

$|g_{PL}|$= 1.41 × 10^{-3} in toluene

Adv. Mater. **2024**, *36*, 2307420

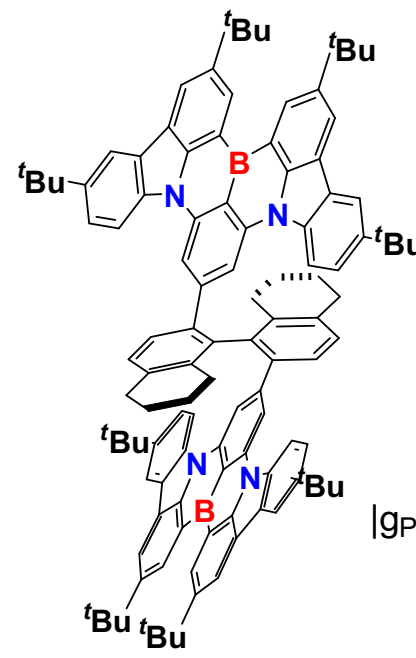

DtCzB-OBN |g_{PL}|= 6.54 × 10^{-4}

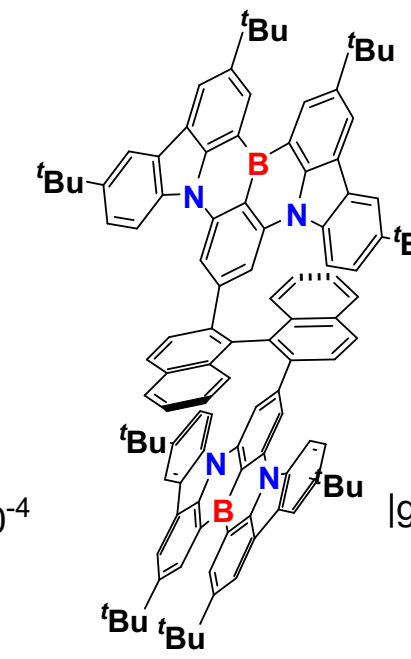

DtCzB-BN |g_{PL}|= 1.82 × 10^{-3}

Both in 5 wt% doped films in 2,6-DCzPPy

Adv. Optical Mater. **2024**, *12*, 2400685

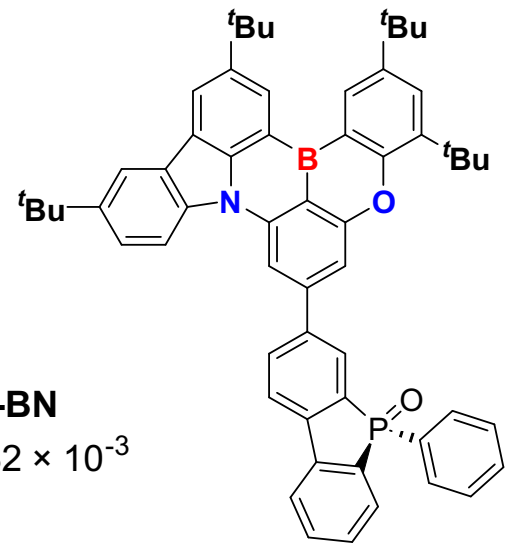

NBOPO |g_{PL}|= 1.18 × 10^{-3}

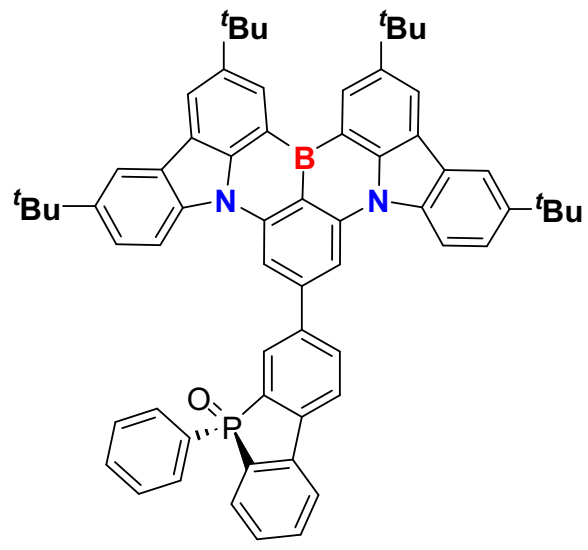

NBNPO |g_{PL}|= 4.30 × 10^{-3}

Both in 5 wt% doped films in mCBP

Mater. Horiz. **2024**,*11*, 4722

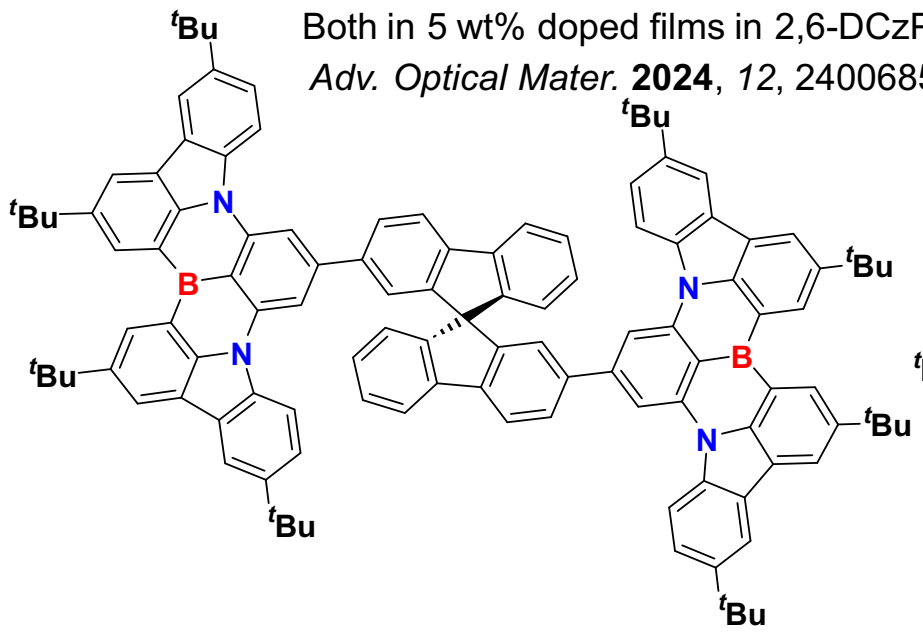

***p*-Spiro-DtBuCzB**

|g_{PL}|= 3.28 × 10^{-4} in toluene

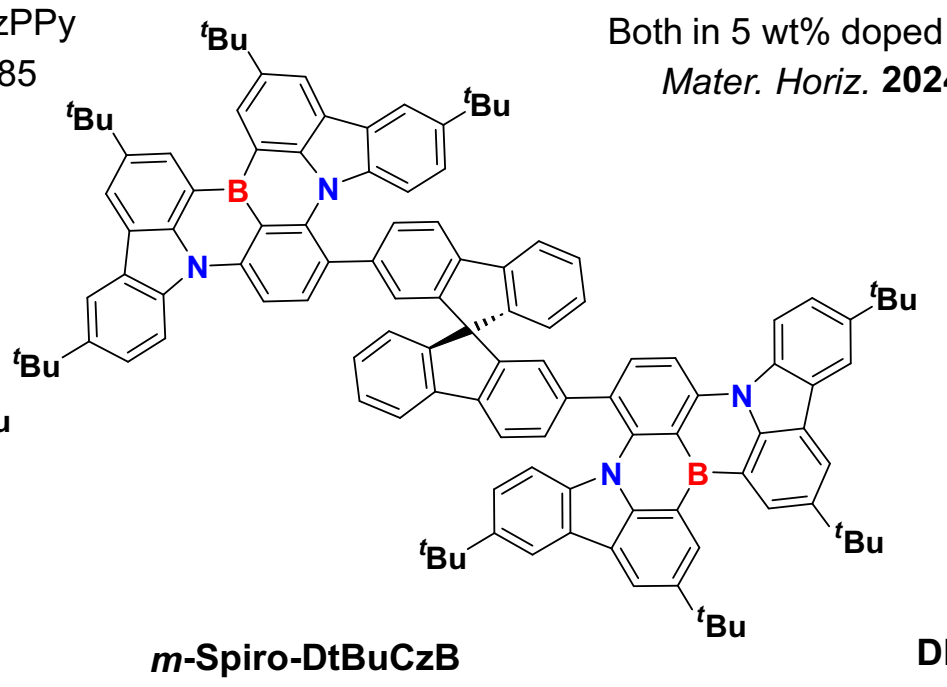

***m*-Spiro-DtBuCzB**

|g_{PL}|= 6.61 × 10^{-4} in toluene

Sci. China. Chem. **2024**, *67*, 2257

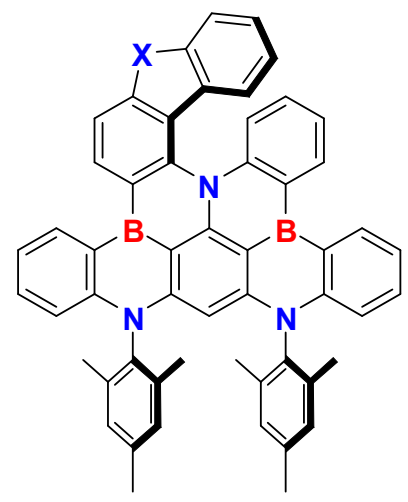

DB-O: X = O |g_{PL}|= 1.4 × 10^{-3} in toluene

DB-S: X = S |g_{PL}|= 1.5 × 10^{-3} in toluene

Adv. Mater. **2024**, *36*, 2308314

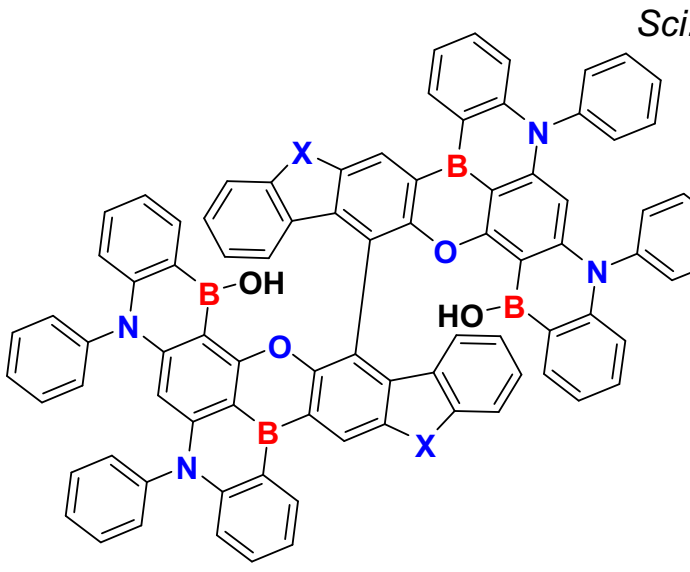

BDBF-BOH: X = O |g_{PL}|= 6.8 × 10^{-4} in toluene

BDBT-BOH: X = S |g_{PL}|= 8.5 × 10^{-4} in toluene

Angew. Chem. Int. Ed. **2024**, *63*, e202407277

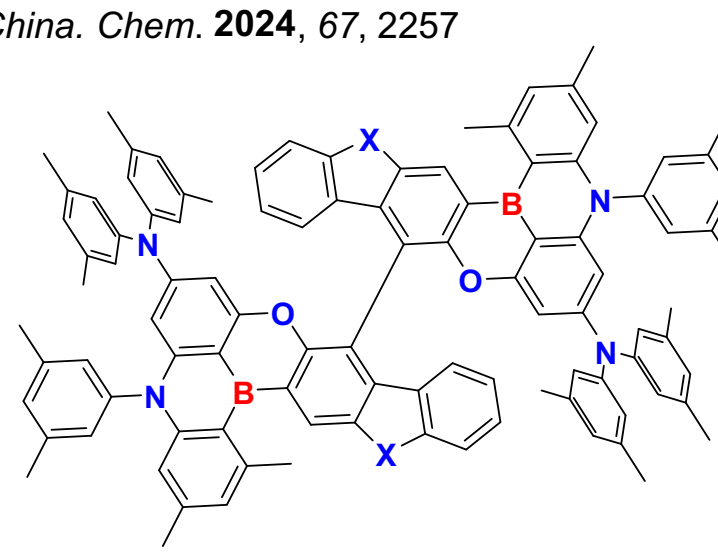

BDBF-BNO: X = O |g_{PL}|= 1.7 × 10^{-3} in toluene

BDBT-BNO: X = S |g_{PL}|= 1.8 × 10^{-3} in toluene

Adv. Funct. Mater. **2024**, *2403803*

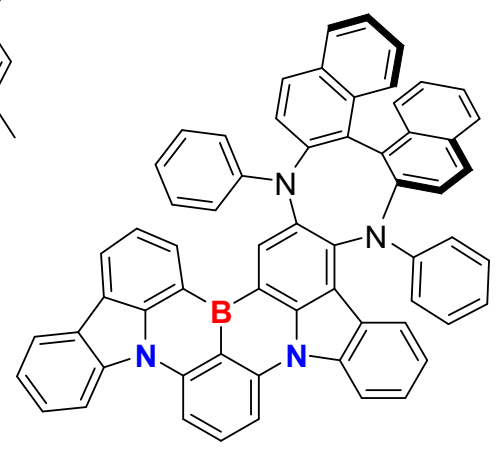

BA34CzBN |g_{PL}|= 3.3 × 10^{-3} in pure film

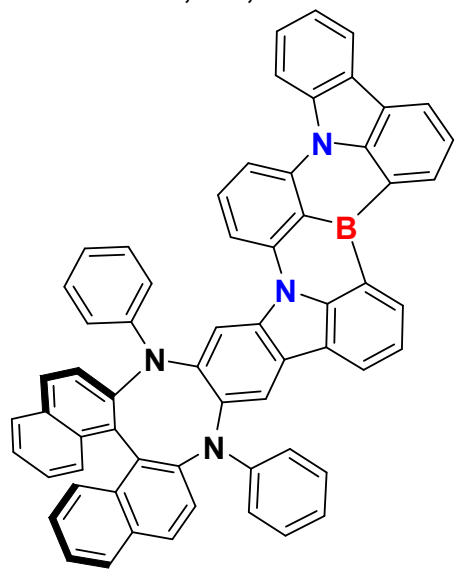

BA23CzBN |g_{PL}|= 0.77 × 10^{-3} in pure film

Adv. Funct. Mater. **2024**, *34,* 2403191

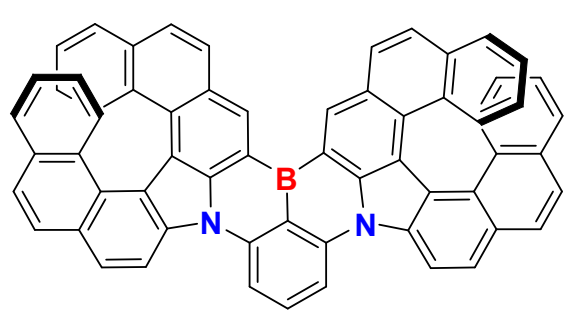

3-C$_2$ |g_{PL}|= 1.5 × 10^{-4} in solution

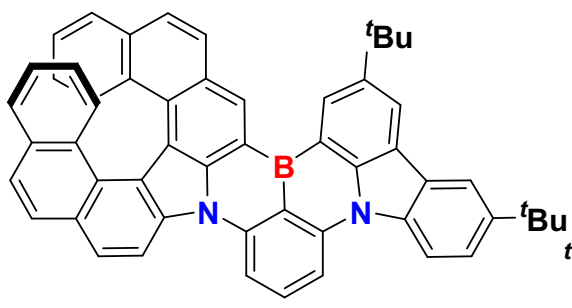

4-C$_1$ |g_{PL}|= 1.1 × 10^{-4} in solution

Angew. Chem. Int. Ed. **2023**, *135,* e202218965

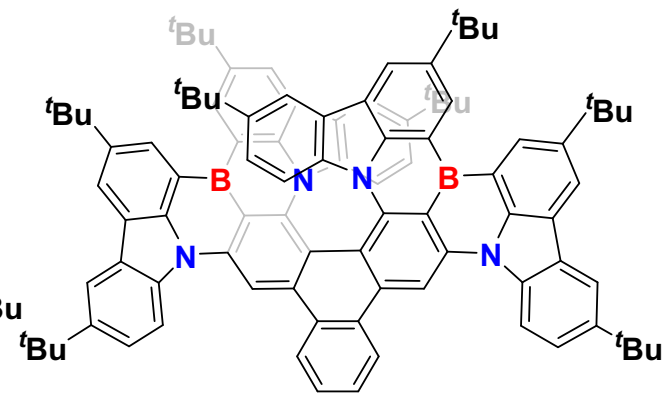

BN[9]H |g_{PL}|= 5.8 × 10^{-3} in toluene

Angew. Chem. Int. Ed. **2024**, e202401835

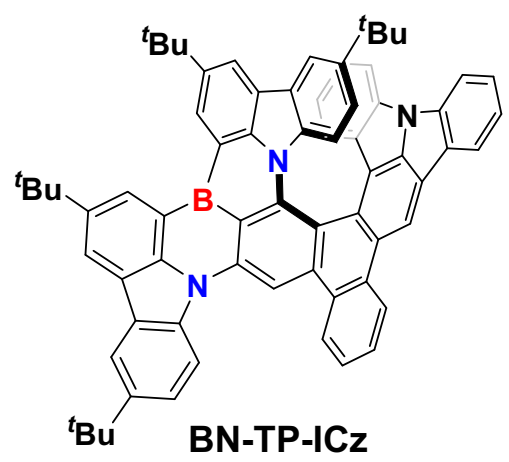

BN-TP-ICz |g_{PL}|= 5.8 × 10^{-4} in toluene

Chem. Sci. **2024**, *15*, 15170

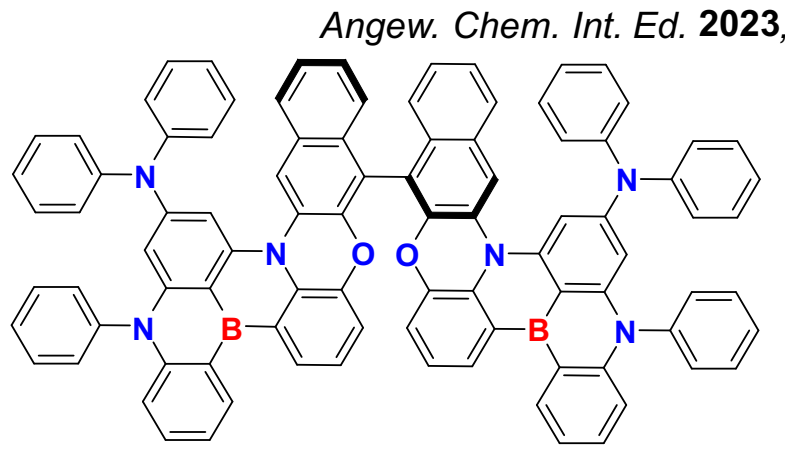

BIPNX-BN

|g_{PL}|= 1.8 × 10^{-3} in toluene

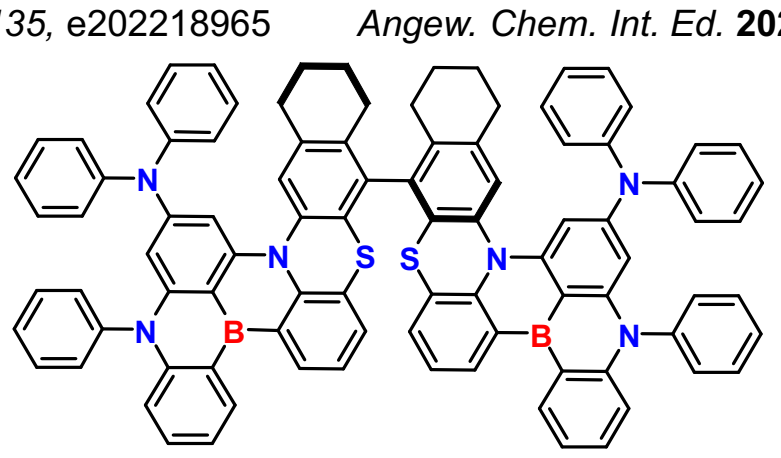

8*H*-BIPTZ-BN

|g_{PL}|= 0.4 × 10^{-3} in toluene

Chem. Eng. J. **2025**, *505*, 159719

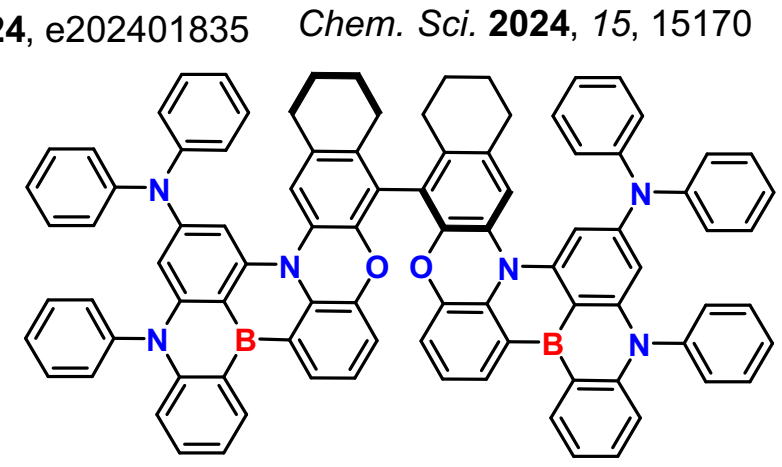

8*H*-BIPNX-BN

|g_{PL}|= 1.2 × 10^{-3} in toluene

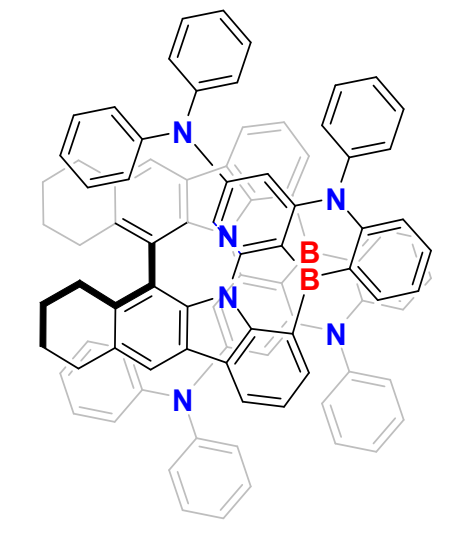

R/S-OBBCz-BN

$|g_{PL}|$ = 2.5×10^{-3} in hexane

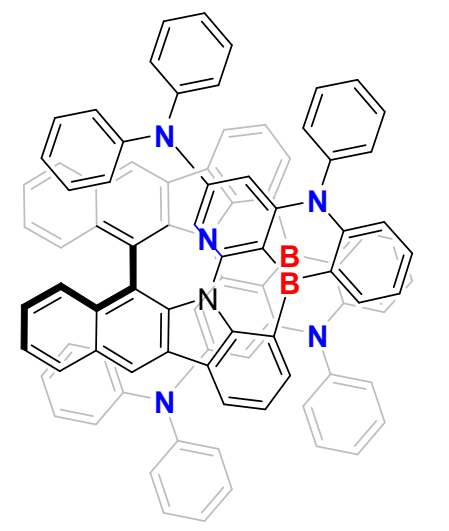

R/S-BBCz-BN

$|g_{PL}|$ = 5.8×10^{-3} in toluene

Adv. Funct. Mater. **2025**, 2421020

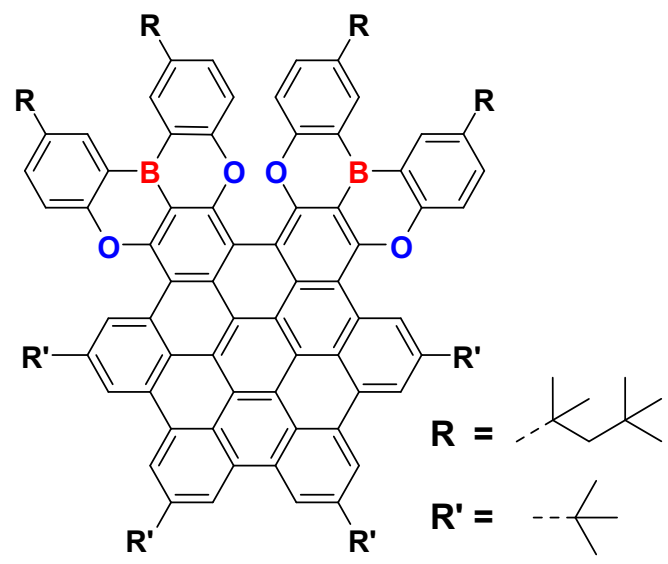

B2NG

$|g_{PL}|$= 2.5 × 10^{-3} in toluene

Chem. Eur. J. **2024**, *30*, e202304169

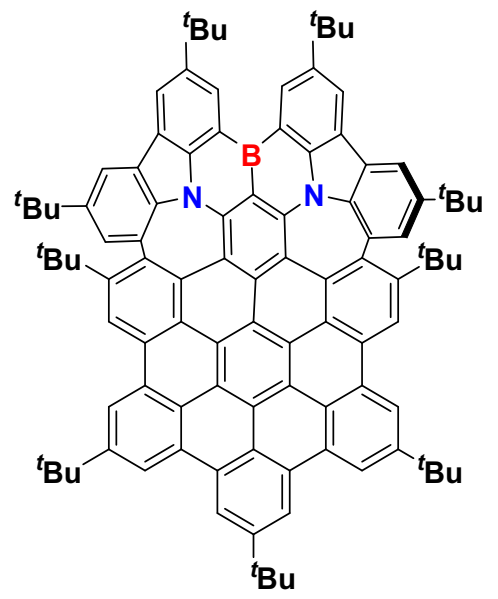

1c

$|g_{PL}|$= 5.0 × 10^{-3} in DCM

Angew. Chem. Int. Ed. **2024**, *137*, e202414383

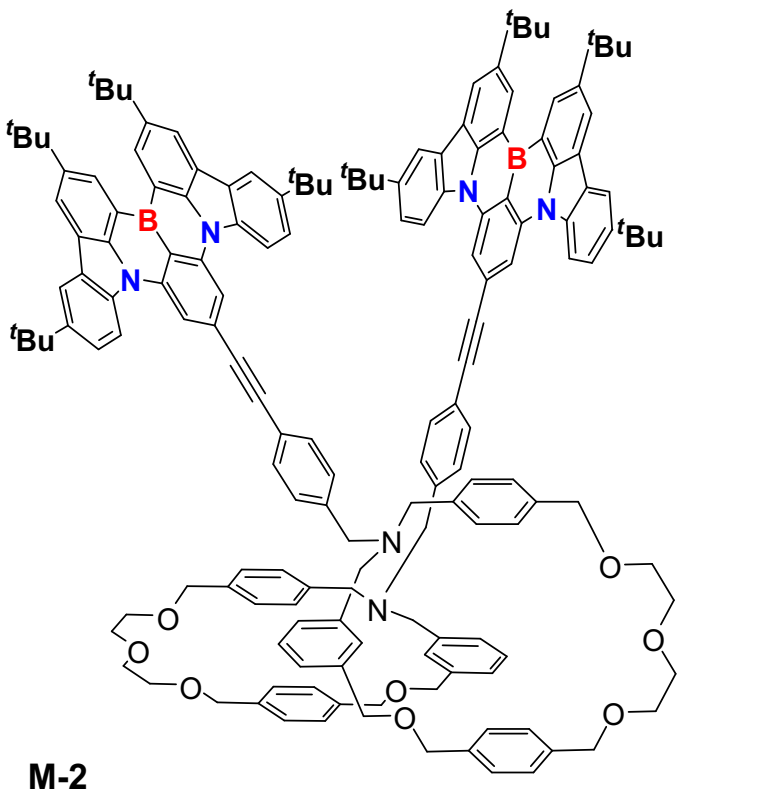

M-2

$|g_{PL}|$ = 1.6×10^{-2} (3 wt% doped film in DMIC-TRZ)

Angew. Chem. Int. Ed. **2025**, *64*, e202417458

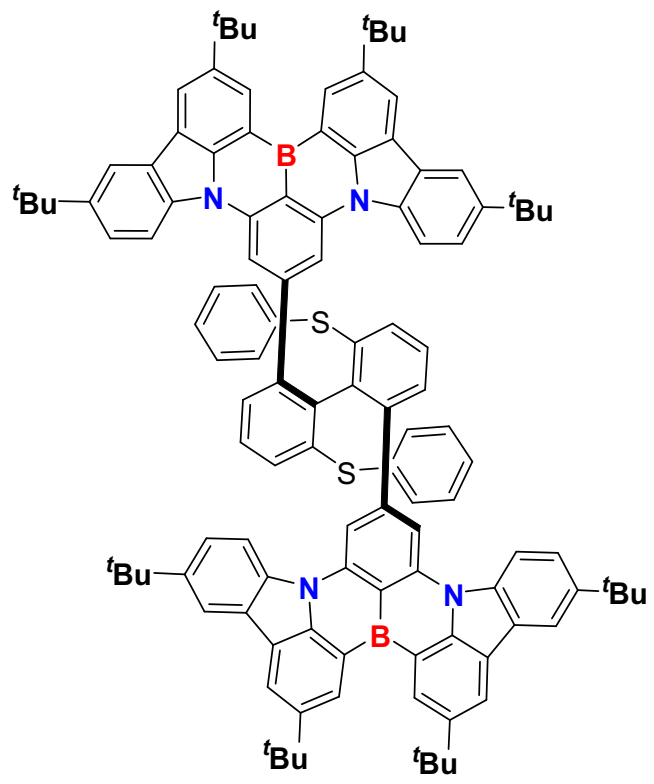

S-AX-BN

$|g_{PL}|$=2.2 × 10^{-3} in toluene

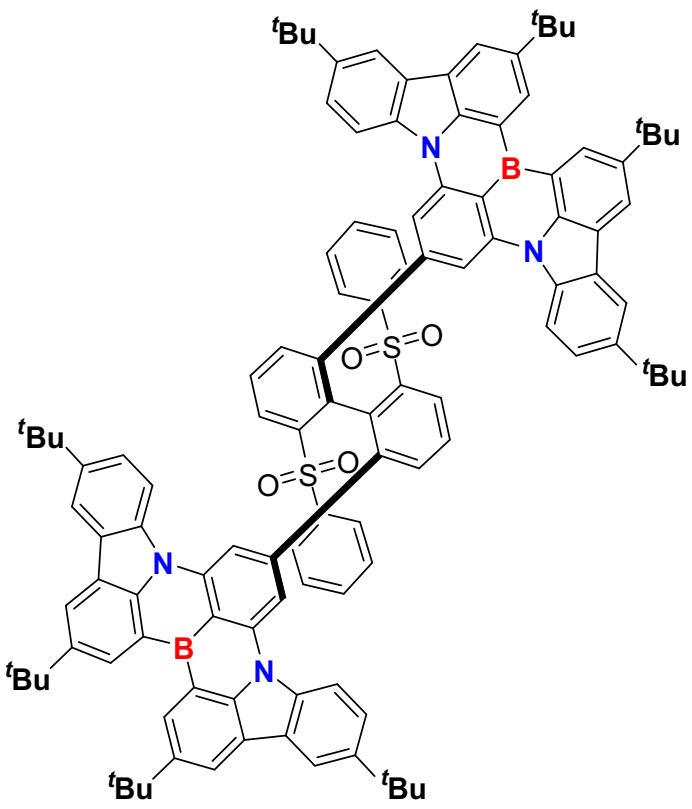

SO$_2$-AX-BN

$|g_{PL}|$=1.4 × 10^{-3} in toluene

Adv. Funct. Mater. **2025**, *35*, 2412044

Supplementary Figure **1**. Molecular structures. CP-MR-TADF emitters reported in the literature

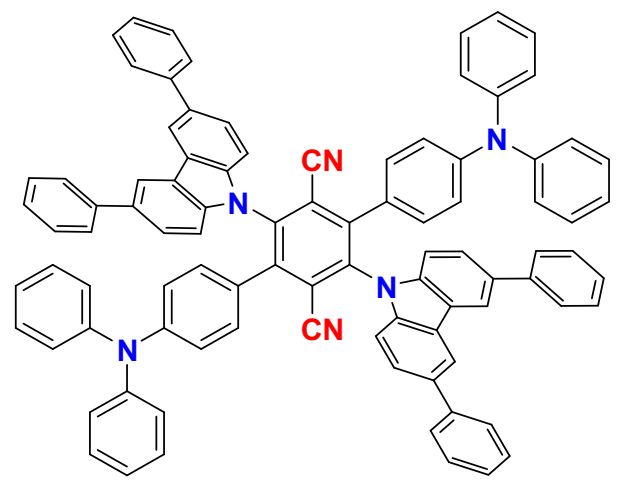

TPCzTPA

Φ = 97% (5 wt% doped film in mCBP)

τ_D = 4.2 μs (5 wt% doped film in mCBP)

$\sigma_{860}{}^2$ = 81 GM (in Toluene)

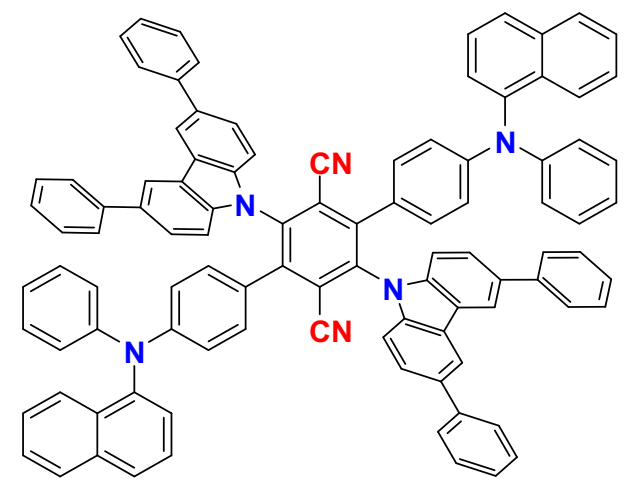

TPCz1NP

Φ = 86% (5 wt% doped film in mCBP)

τ_D = 5.7 μs (5 wt% doped film in mCBP)

$\sigma_{850}{}^2$ = 131 GM (in Toluene)

Angew. Chem. Int. Ed. **2025**, *137*, e202420417

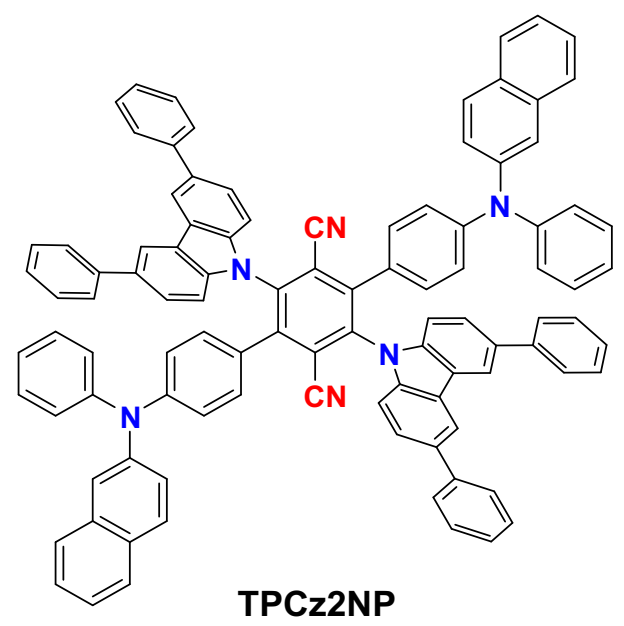

TPCz2NP

Φ = 100% (5 wt% doped film in mCBP)

τ_D = 4.6 μs (5 wt% doped film in mCBP)

$\sigma_{850}{}^2$ = 143 GM (in Toluene)

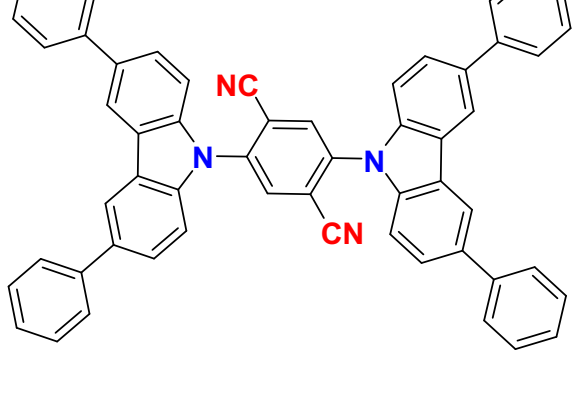

2PhCzTPN

Φ = 64% (5 wt% doped film in mCBP)

τ_D = 4.2 μs (5 wt% doped film in mCBP)

$\sigma_{790}{}^2$ = 40 GM (in Toluene)

Angew. Chem. Int. Ed. **2025**, *137*, e202420417

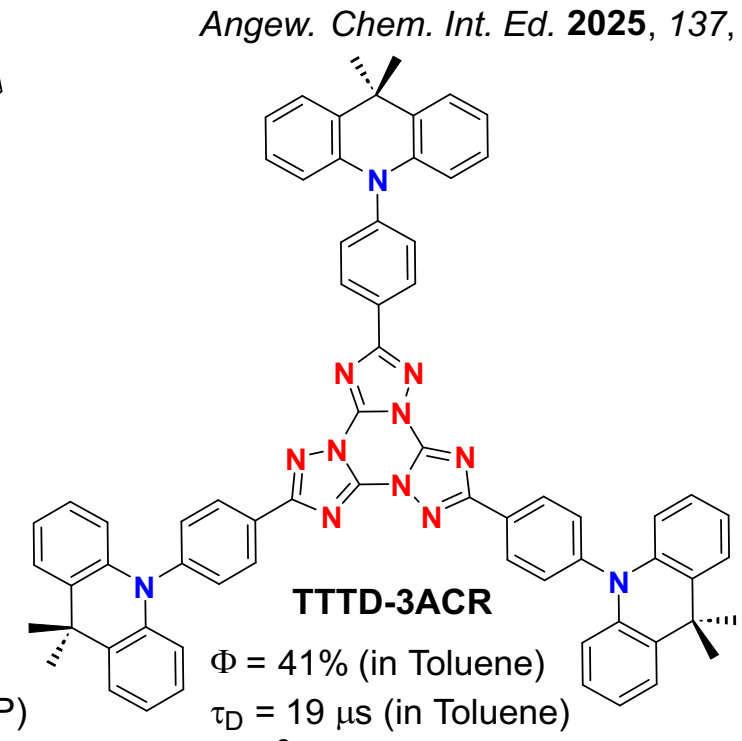

TTTD-3ACR

Φ = 41% (in Toluene)

τ_D = 19 μs (in Toluene)

$\sigma_{740}{}^2$ = 83 GM (in Toluene)

J. Mater. Chem. C, **2021**, *9*, 14342

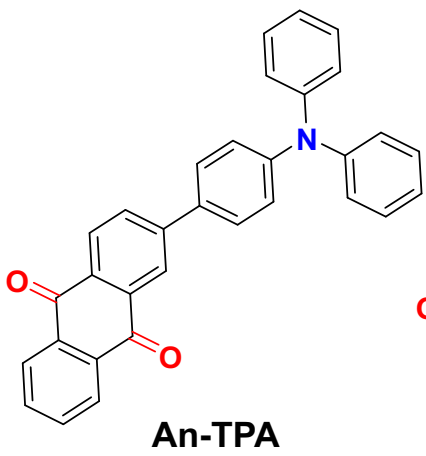

An-TPA

Φ = 3.7%

(Nanoparticles in water)

τ_D = 1.3 μs

(10 wt% doped film in DPEPO)

σ^2 not provided

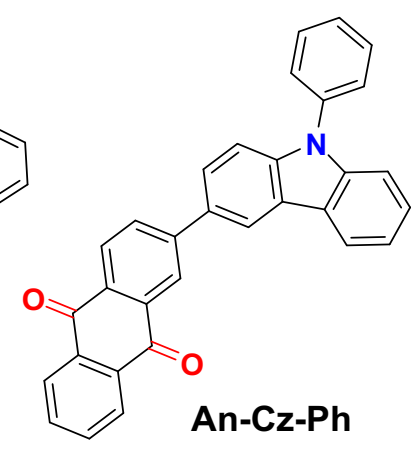

An-Cz-Ph

Φ = 0.8%

(Nanoparticles in water)

τ_D = 2.4 μs

(10 wt% doped film in DPEPO)

σ^2 not provided

ACS Appl. Mater. Interfaces, **2019**, *11*, 41051

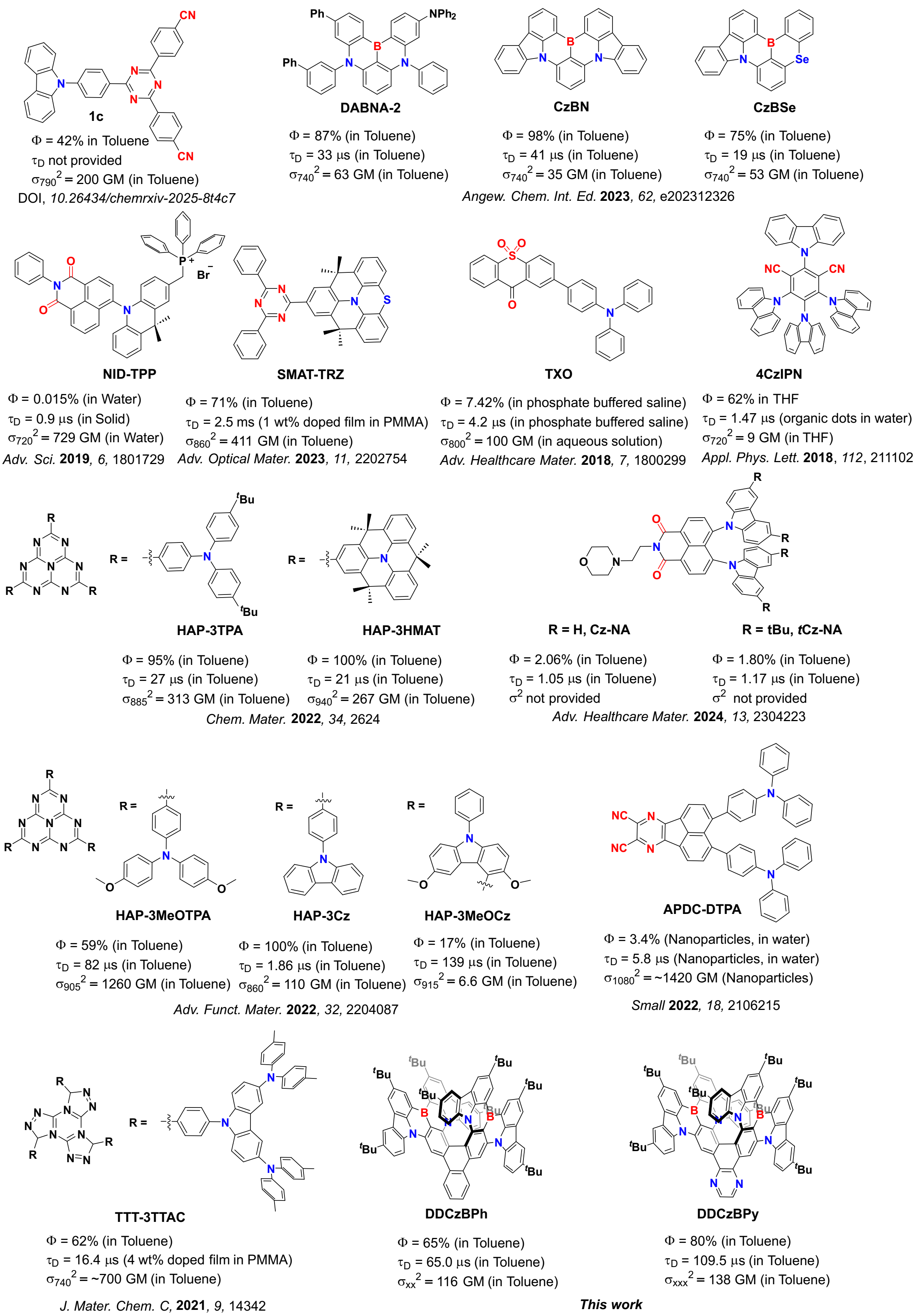

Supplementary Figure **2**. Molecular structures. 2PA-TADF emitters reported in the literature

3. Experimental section

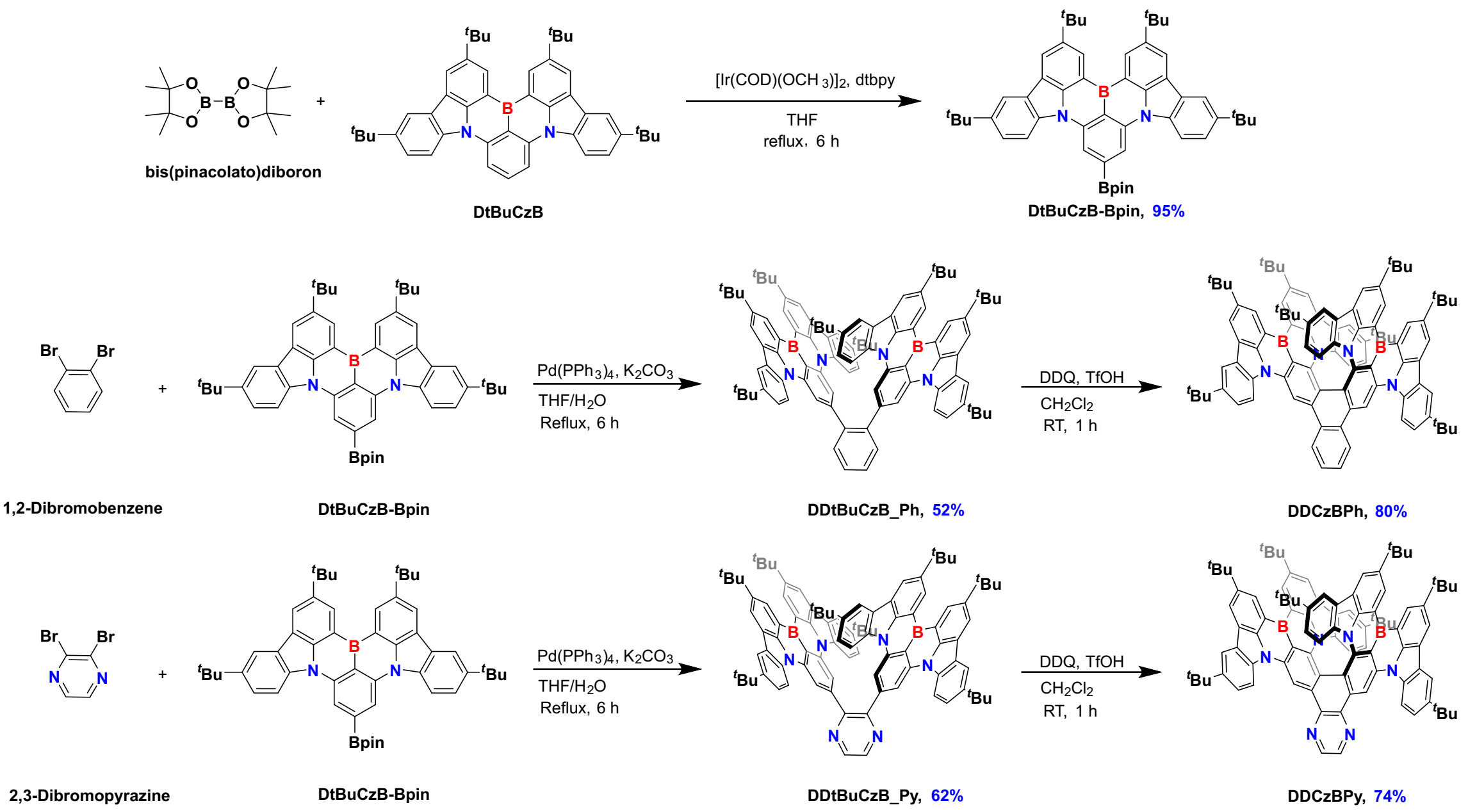

Supplementary Figure **3**. Synthetic procedures for **DDCzBPh** and **DDCzBPy**

Synthesis of DtBuCzB_Bpin

Under a N_2 atmosphere, a mixture of $[Ir(COD)(OCH_3)]_2$ (43.1 mg, 0.065 mmol), dtbpy (34.9 mg, 0.13 mmol), DtCzB (4.20 g, 6.5 mmol) and bis(pinacolato)diboron (B_2Pin) (1.68 g, 6.6 mmol) were dissolved in extra dry tetrahydrofuran (THF) (60 mL) at room temperature. Then the mixture was heated to reflux and stirred for 6 h. After cooling to room temperature, the reaction mixture was directly concentrated under reduced pressure and purified by silica gel flash column chromatography using DCM:hexane = 1:2 as the eluent to afford the desired compound as a yellow solid. **Yield:** 52%. **R_f**: 0.25 (DCM:hexane = 1:2). **^{1}H NMR (400 MHz, $CDCl_3$) δ (ppm):** 9.14 (d, J = 1.9 Hz, 2H), 8.79 (s, 2H), 8.54 (d, J = 8.8 Hz, 2H), 8.48 (d, J = 1.8 Hz, 2H), 8.27 (d, J = 2.0 Hz, 2H), 7.74 (dd, J = 8.8, 2.1 Hz, 2H), 1.67 (s, 18H), 1.50 (s, 12H). The analytical data match those in the literature.[1]

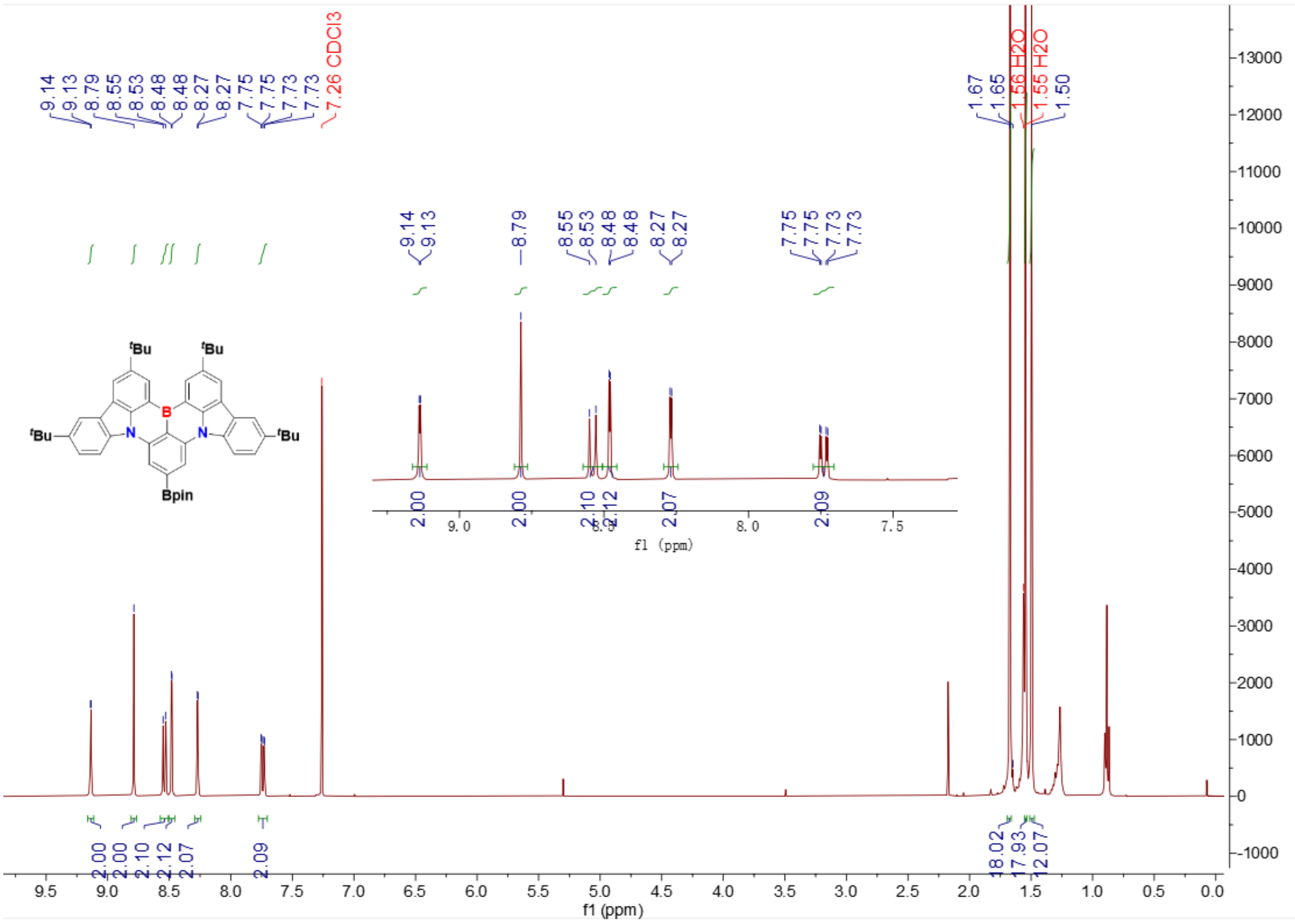

Supplementary Figure **4**. ^{1}H-NMR spectrum of **DDtBuCzB_Bpin** in $CDCl_3$ (400 MHz).

Synthesis of DDtBuCzB_Ph

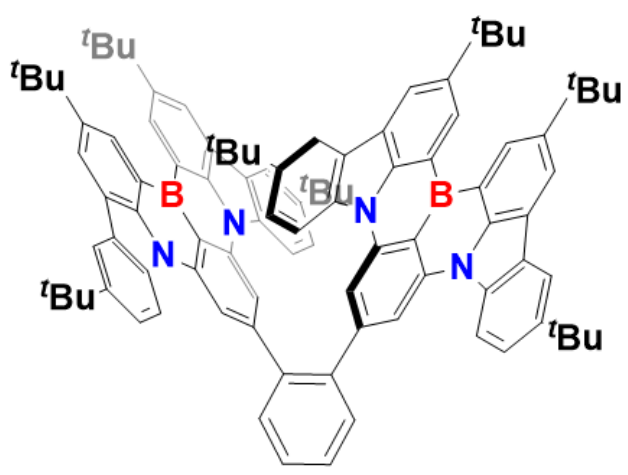

Under a N_2 atmosphere, a mixture of 1,2-dibromobenzene (200.0 mg, 0.85 mmol, 1.0 equiv.), **DtBuCzB-Bpin** (1.14 g, 1.78 mmol, 2.1 equiv.), $Pd(PPh_3)_4$ (49.0 mg, 42.4 μmol 0.05 equiv.) and K_3PO_4 (601.8 mg, 2.54 mmol, 3 equiv.) were dissolved in a mixture of THF and H_2O (10 mL and 3 mL). The reaction mixture was heated to reflux and stirred for 12 h. The reaction mixture was cooled to room temperature and the product was filtered and washed with water (3 × 50 mL) and dichloromethane (3 × 30 mL). The solvents were removed under reduced pressure and the crude mixture was purified by silica gel flash column chromatography using DCM:hexane = 1:5 as the eluent to afford the desired compound as a yellow solid. **Yield:** 52%. **Mp:** 329-331°C. **R*f***: 0.2 (DCM:hexane = 1:5). **^{1}H NMR (500 MHz, $CDCl_3$) δ (ppm):** 8.96 (d, *J* = 1.9 Hz, 4H), 8.53 (s, 4H), 8.33 (d, *J* = 1.8 Hz, 4H), 8.05 (d, *J* = 2.1 Hz, 4H), 7.99-7.94 (m, 6H), 7.73 (dd, *J* = 5.7, 3.3 Hz, 2H), 7.00 (d, *J* = 8.4 Hz, 4H), 1.57 (s, 36H), 1.13 (s, 36H). **^{13}C NMR (126 MHz, $CDCl_3$) δ (ppm)**: 146.3, 145.4, 144.7, 144.2, 141.9, 141.6, 138.1, 131.3, 129.8, 128.9, 126.9, 124.4, 123.8, 122.2, 121.7, 120.7, 117.1, 113.9, 110.8, 35.2, 34.5, 32.3, 31.6. The analytical data match those in the literature.[1]

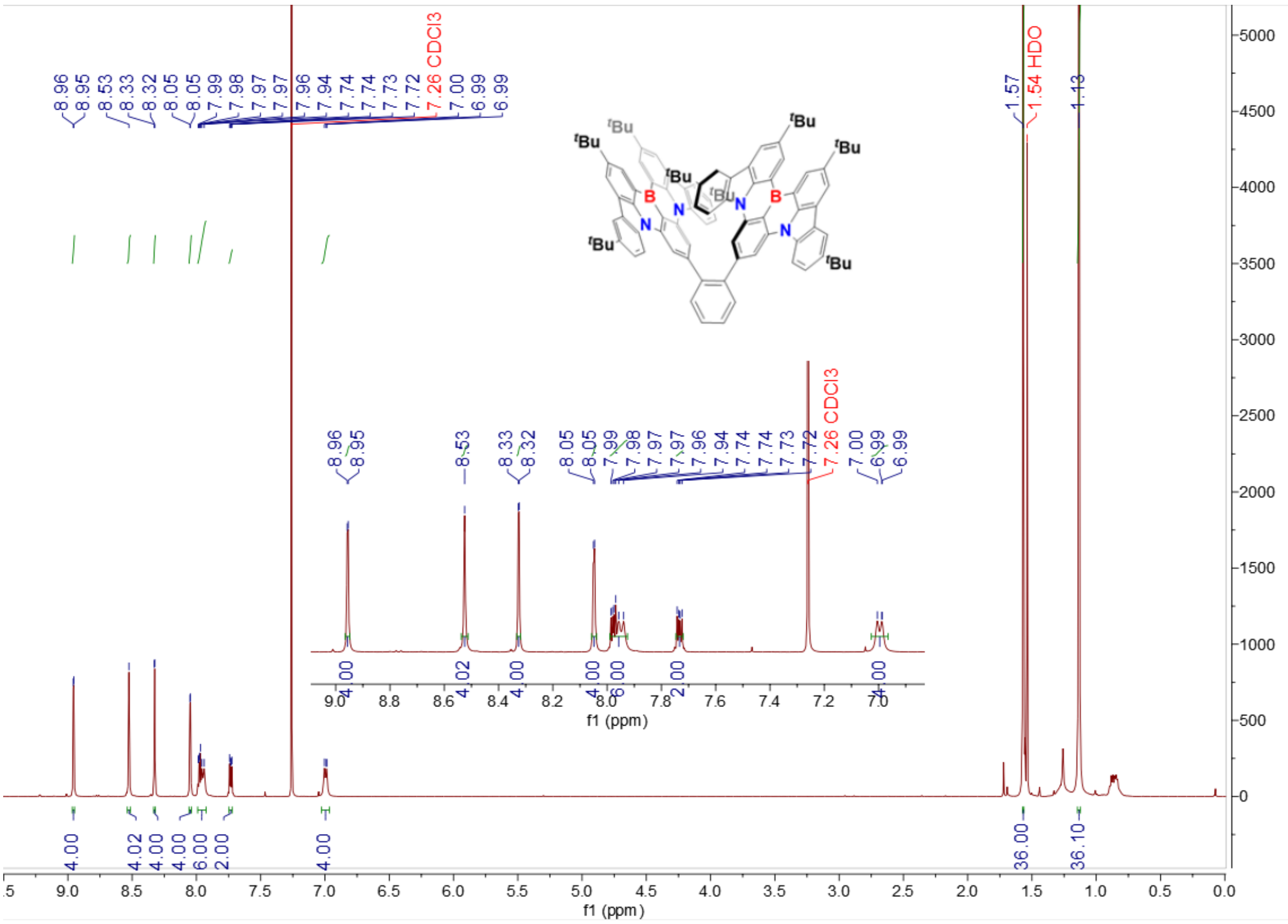

Supplementary Figure **5**. ^{1}H-NMR spectrum of **DDtBuCzB_Ph** in $CDCl_3$ (500 MHz).

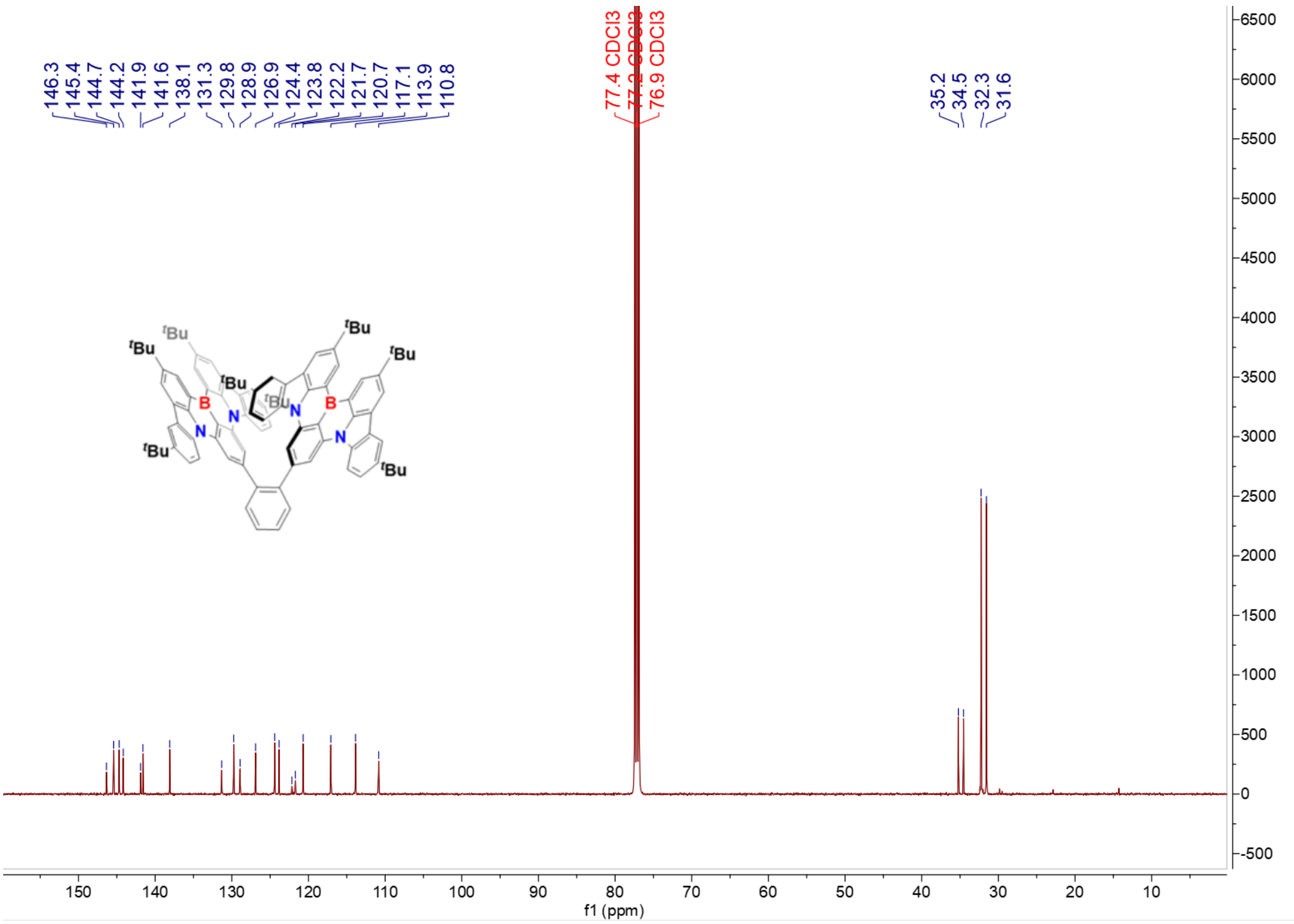

Supplementary Figure **6**. ^{13}C-NMR spectrum of **DDtBuCzB_Ph** in $CDCl_3$ (126 MHz).

Synthesis of DDCzBPh

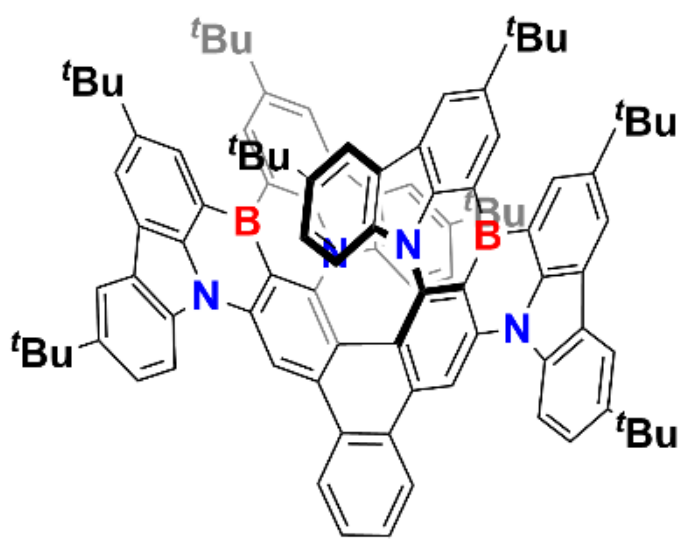

Under a N_2 atmosphere, **DDtBuCzB_Ph** (500.0 mg, 0.37 mmol, 1.0 equiv.) and 2,3-dichloro-5,6-dicyano-1,4-benzoquinone (DDQ, 100.5 mg, 0.44 mmol, 1.2 equiv.) were dissolved in 50 mL of DCM. The solution was cooled to 0 °C, and TfOH (1 mL) was added dropwise. The reaction mixture was allowed to warm to room temperature and stirred for 1 hour. The reaction mixture was filtered through a celite pad and the insoluble residue was washed with dichloromethane (100 mL). The filtrates were combined, the solvent was removed under reduced pressure, and the residue was purified by silica gel flash column chromatography using DCM:hexane = 1:5 as the eluent to afford the desired compound as an orange solid. **Yield**: 80%. **Mp**: > 400 °C. **R_f**: 0.13 (DCM:hexane = 1:5). **^{1}H NMR (500 MHz, CD_2Cl_2) δ (ppm)**: 9.73 (d, J = 1.1 Hz, 2H), 9.18 (dd, J = 6.2, 3.4 Hz, 2H), 8.97 (d, J = 1.9 Hz, 2H), 8.92 (d, J = 8.7 Hz, 2H), 8.82 (d, J = 1.8 Hz, 2H), 8.55 (d, J = 1.8 Hz, 2H), 8.41 (d, J = 2.0 Hz, 2H), 8.05 (dd, J = 6.2, 3.1 Hz, 2H), 7.94 (dd, J = 8.7, 2.1 Hz, 2H), 7.77 (d, J = 1.7 Hz, 2H), 7.24 (d, J = 1.9 Hz, 2H), 5.94 (dd, J = 8.6, 2.0 Hz, 2H), 5.84 (d, J = 8.6 Hz, 2H), 1.76 (s, 18H), 1.69 (s, 18H), 1.63 (s, 18H), 0.68 (s, 18H). **^{13}C NMR (126 MHz, CD_2Cl_2) δ (ppm)**: 145.8, 145.0, 144.9, 144.8, 141.8, 141.5, 141.3, 138.9, 138.8, 136.1, 135.1, 132.1, 130.6, 129.6, 129.3, 127.6, 126.7, 125.1, 125.0, 124.4, 123.7, 121.3, 120.8, 119.7, 117.9, 116.5, 114.4, 112.6, 112.4, 102.4, 35.5, 35.5, 35.2, 34.2, 32.7, 32.4, 32.1, 31.2. **HR-MS $[M+H]^+$ Calculated:** ($C_{98}H_{99}B_2N_4$) 1353.8050; **Found:** 1353.8015. **EA calculated:** ($C_{98}H_{98}B_2N_4$) C, 86.96%; H, 7.30%; N, 4.14%. **Found:** C, 86.97%; H, 6.51%; N, 3.60%. **HPLC purity:** > 99% (retention time: 14.420 minutes and 18.781 minutes for ***P*-DDCzBPh** and ***M*-DDCzBPh**, respectively, employing an eluent consisting of DCM and hexane with a ratio of 3:17). The analytical data match those in the literature.[1]

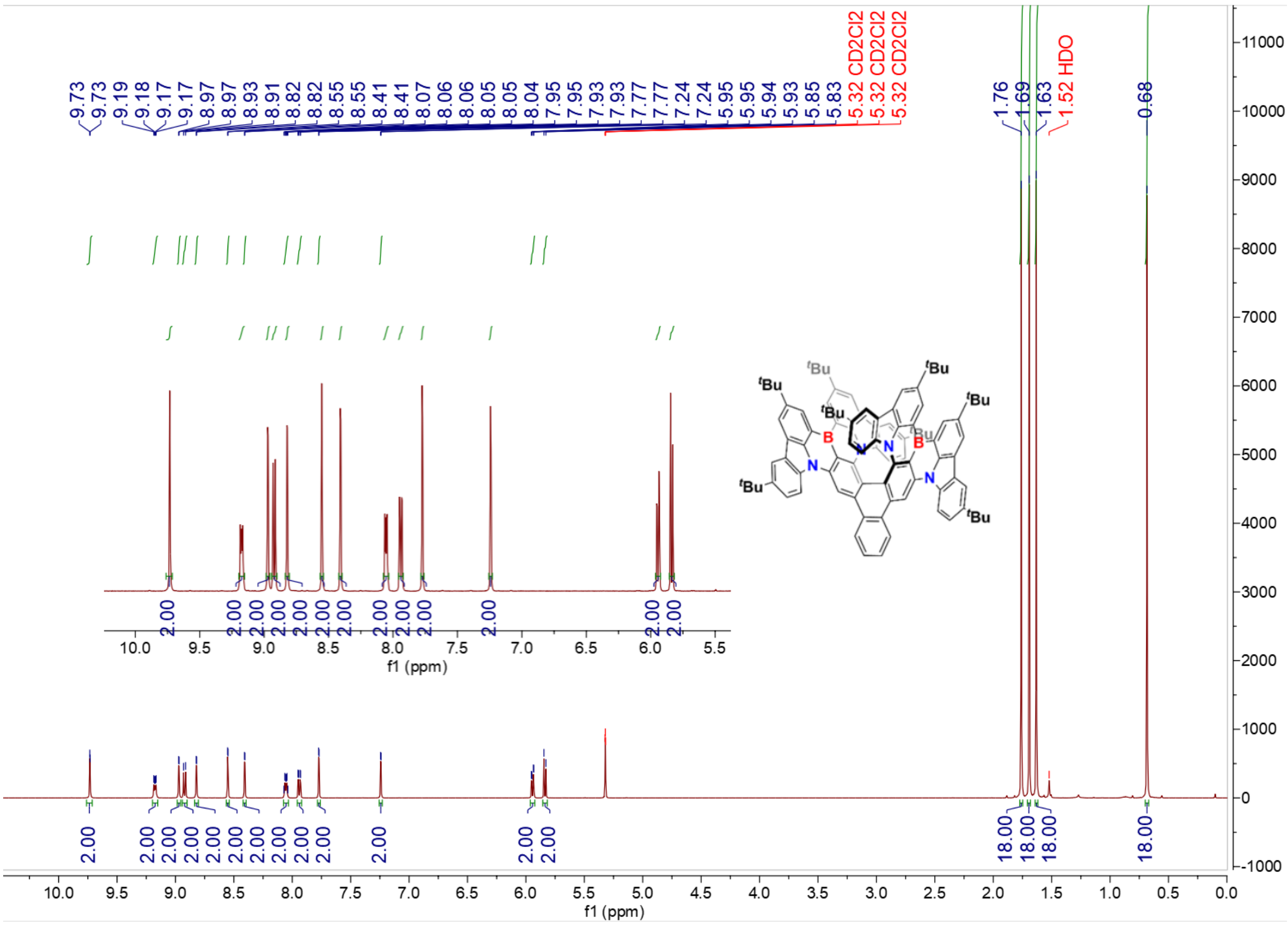

Supplementary Figure **7**. ^{1}H-NMR spectrum of **DDCzBPh** in CD_2Cl_2 (500 MHz).

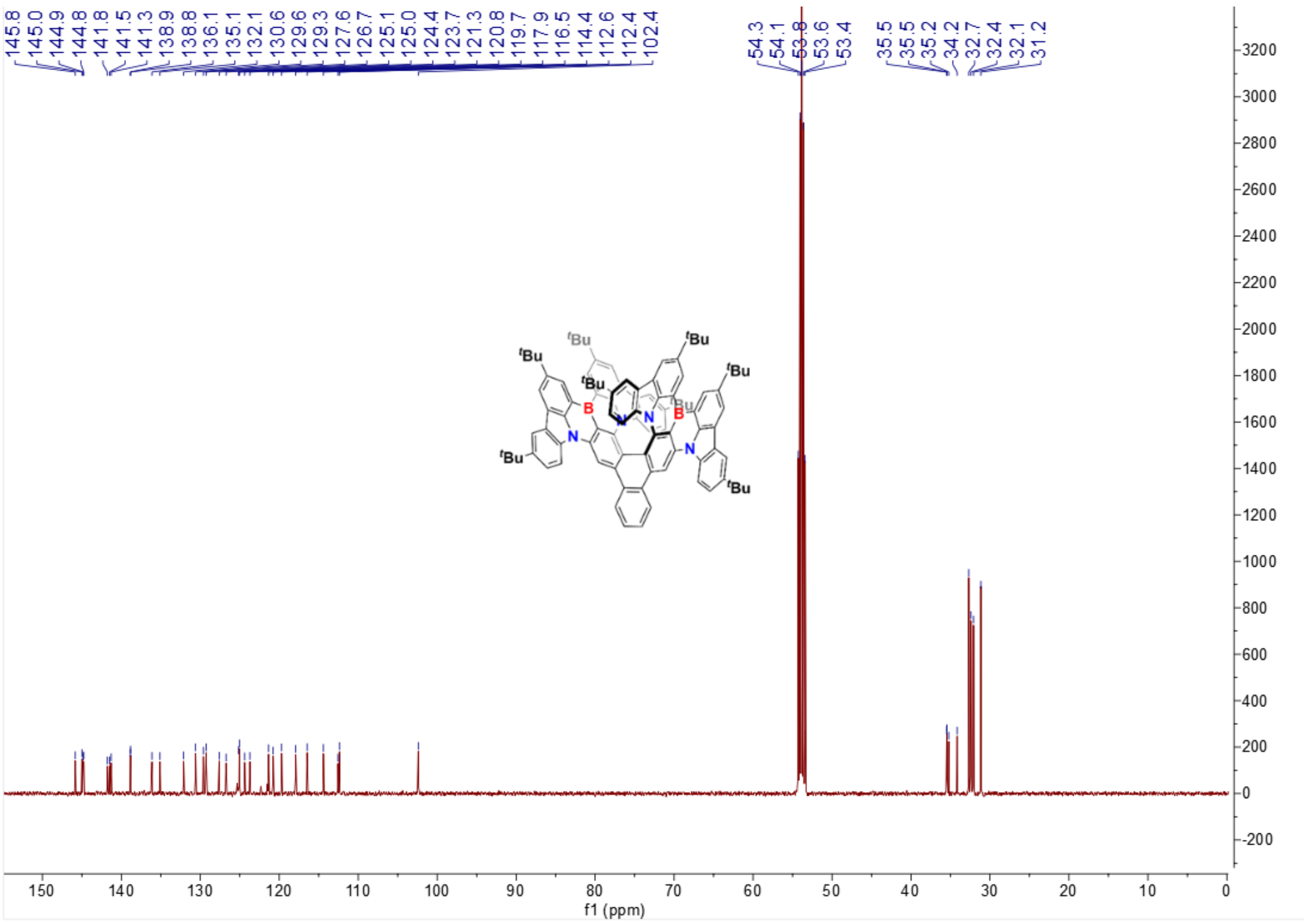

Supplementary Figure **8**. ^{13}C-NMR spectrum of **DDCzBPh** in CD_2Cl_2 (126 MHz).

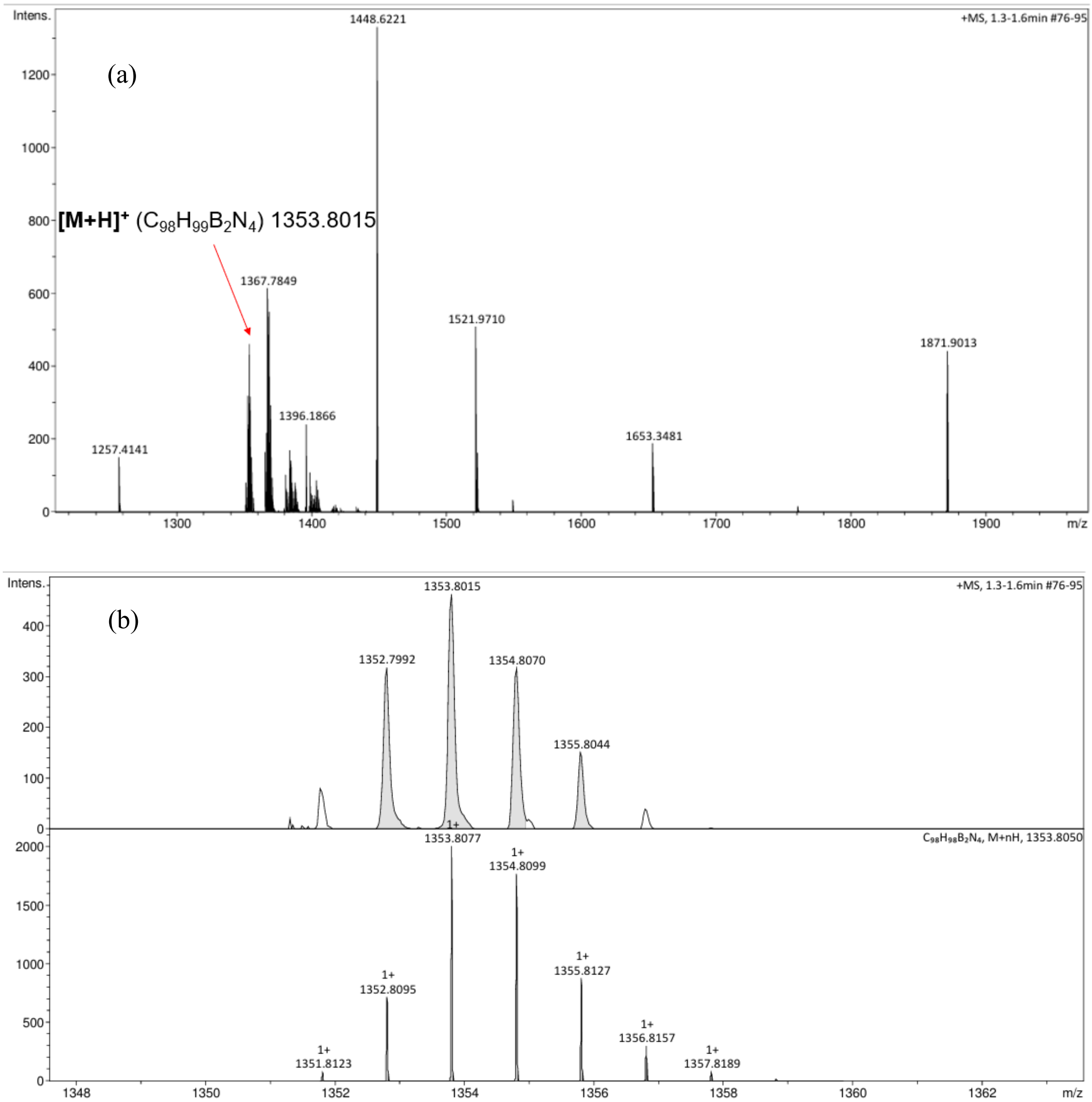

Supplementary Figure **9**. HRMS of **DDCzBPh** (a) full spectrum, (b) **[M+H]**$^{+}$ ($C_{98}H_{99}B_2N_4$)

<Chromatogram>

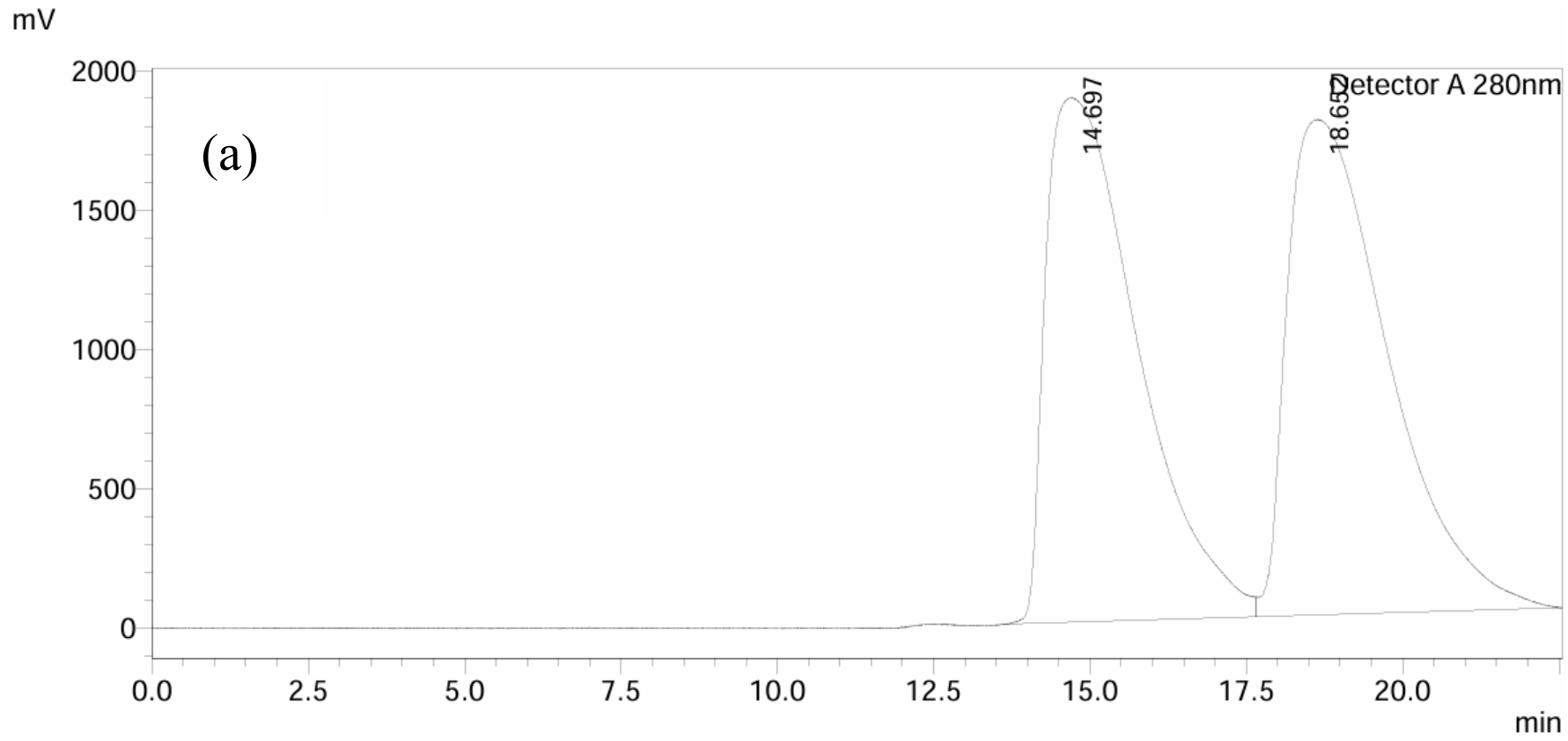

<Peak Table>

Detector A 280nm

Peak#	Ret. Time	Area	Height	Area%	Area/Height	Width at 5% Height
1	14.697	192550863	1880491	49.200	102.394	3.364
2	18.652	198815228	1776726	50.800	111.900	3.695
Total		391366090	3657217	100.000		

<Chromatogram>

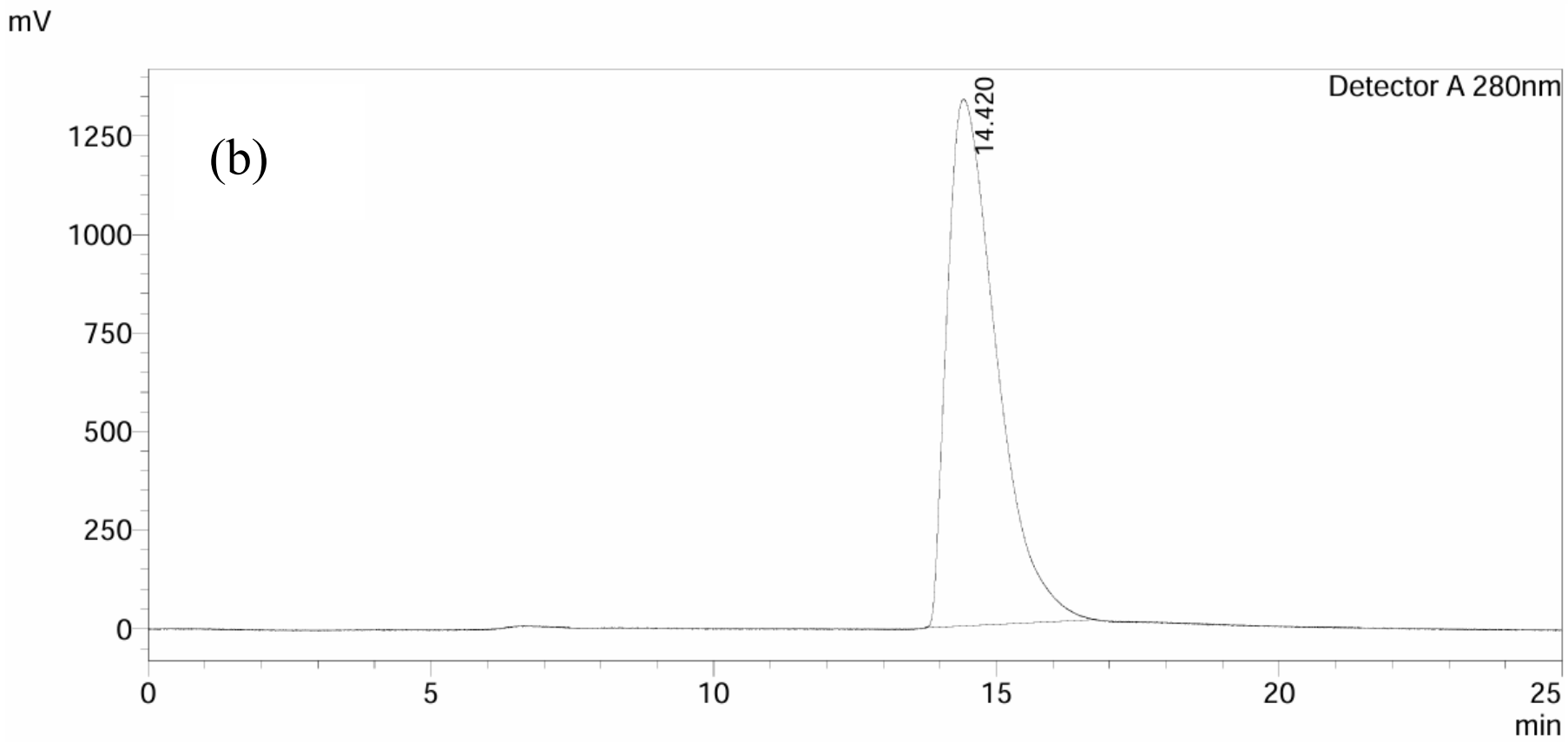

<Peak Table>

Detector A 280nm

Peak#	Ret. Time	Area	Height	Area%	Area/Height	Width at 5% Height
1	14.420	81074732	1336598	100.000	60.658	2.096
Total		81074732	1336598	100.000		

<Chromatogram>

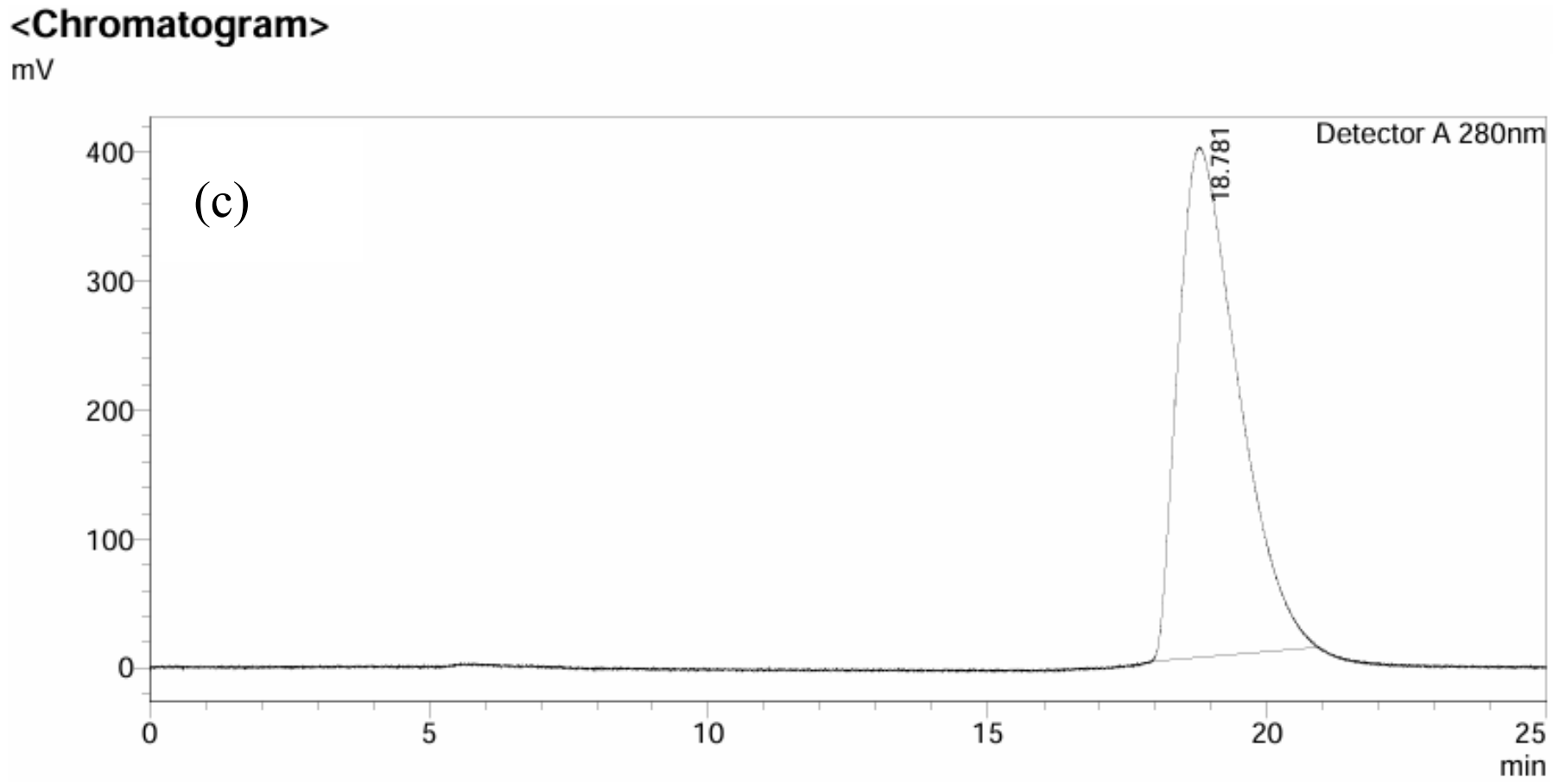

<Peak Table>

Detector A 280nm

Peak#	Ret. Time	Area	Height	Area%	Area/Height	Width at 5% Height
1	18.781	29490889	395480	100.000	74.570	2.450
Total		29490889	395480	100.000		

Supplementary Figure **10**. HPLC report of (a) (*rac*)**-DDCzBPh**, (b) ***P*-DDCzBPh** and (c) ***M*-DDCzBPh**.

LONDON METROPOLITAN UNIVERSITY
HUMAN SCIENCES

Elemental Analysis Sample Results

Name Sen Wu

Organisation Name University of St Andrews

Purchase order number

Standard – Acetanilide		
**Element**	**Expected %**	**Found**
**Carbon**	71.10 (+/- 0.23)	71.02
**Hydrogen**	6.71 (+/- 0.07)	6.64
**Nitrogen**	10.34 (+/- 0.09)	10.29

Analysis – WS-III20			
**Element**	**Expected %**	**Found (1)**	**Found (2)**
**Carbon**	86.96	86.93	87.01
**Hydrogen**	7.30	6.58	6.43
**Nitrogen**	4.14	3.58	3.61

Supplementary Figure **11**. EA results of **DDCzBPh**.

Synthesis of DDtBuCzB_Py

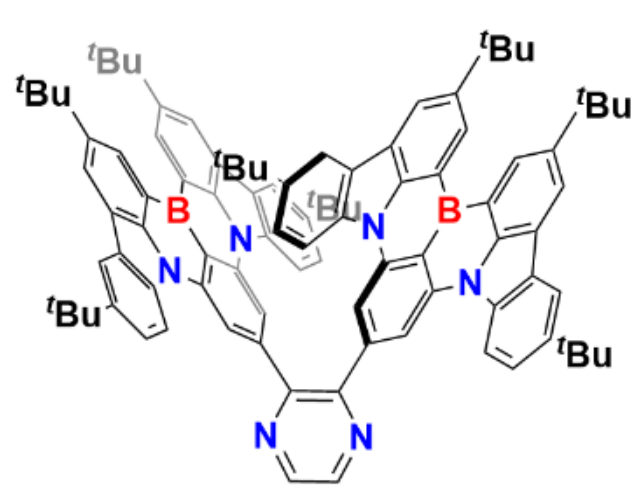

Under a N_2 atmosphere, a mixture of 2,3-dibromopyrazine (200.0 mg, 0.84 mmol, 1.0 equiv.), **DtBuCzB-Bpin** (1.13 g, 1.78 mmol, 2.1 equiv.), $Pd(PPh_3)_4$ (48.0 mg, 42.4 μmol, 0.05 equiv.) and K_3PO_4 (601.8 mg, 2.52 mmol, 3 equiv.) were dissolved in a mixture of THF and H_2O (10 mL and 3 mL). The reaction mixture was heated to reflux and stirred for 12 h. The reaction mixture was cooled to room temperature and the product was filtered and washed with water (3 × 50 mL) and dichloromethane (3 × 30 mL). The filtrates were combined, the solvent was removed

under reduced pressure, and the residue was purified by silica gel flash column chromatography using DCM:hexane = 1:5 as the eluent to afford the desired compound as a yellow solid. **Yield**: 62%. **Mp**: > 400 °C. **R$_f$**: 0.35 (DCM:hexane = 1:2). **^{1}H NMR (500 MHz, CDCl$_3$) δ (ppm)**: 8.96 (d, J = 1.9 Hz, 4H), 8.93 (s, 2H), 8.80 (s, 4H), 8.34 (d, J = 1.8 Hz, 4H), 8.05 (d, J = 2.1 Hz, 4H), 7.88 (d, J = 8.8 Hz, 4H), 6.99 (d, J = 9.1 Hz, 4H), 1.58 (s, 36H), 1.13 (s, 36H). **^{13}C NMR (126 MHz, CDCl$_3$) δ (ppm)**: 154.2, 145.6, 144.9, 144.2, 143.0, 141.6, 138.1, 129.8, 126.9, 124.5, 124.0, 121.7, 120.9, 117.1, 113.8, 110.4, 35.2, 34.6, 32.3, 31.5.

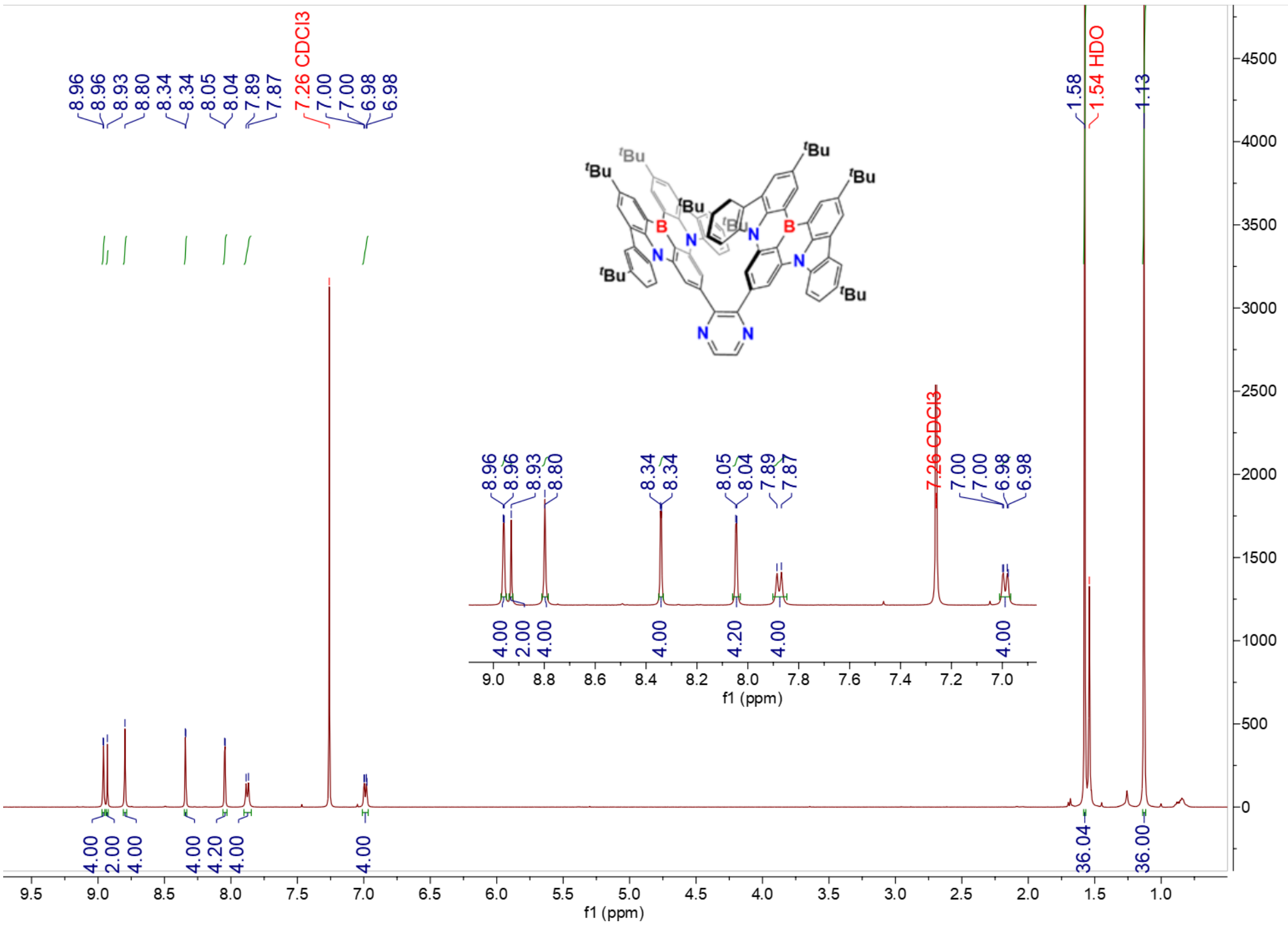

Supplementary Figure **12**. ^{1}H-NMR spectrum of **DDtBuCzB_Py** in CDCl$_3$ (500 MHz).

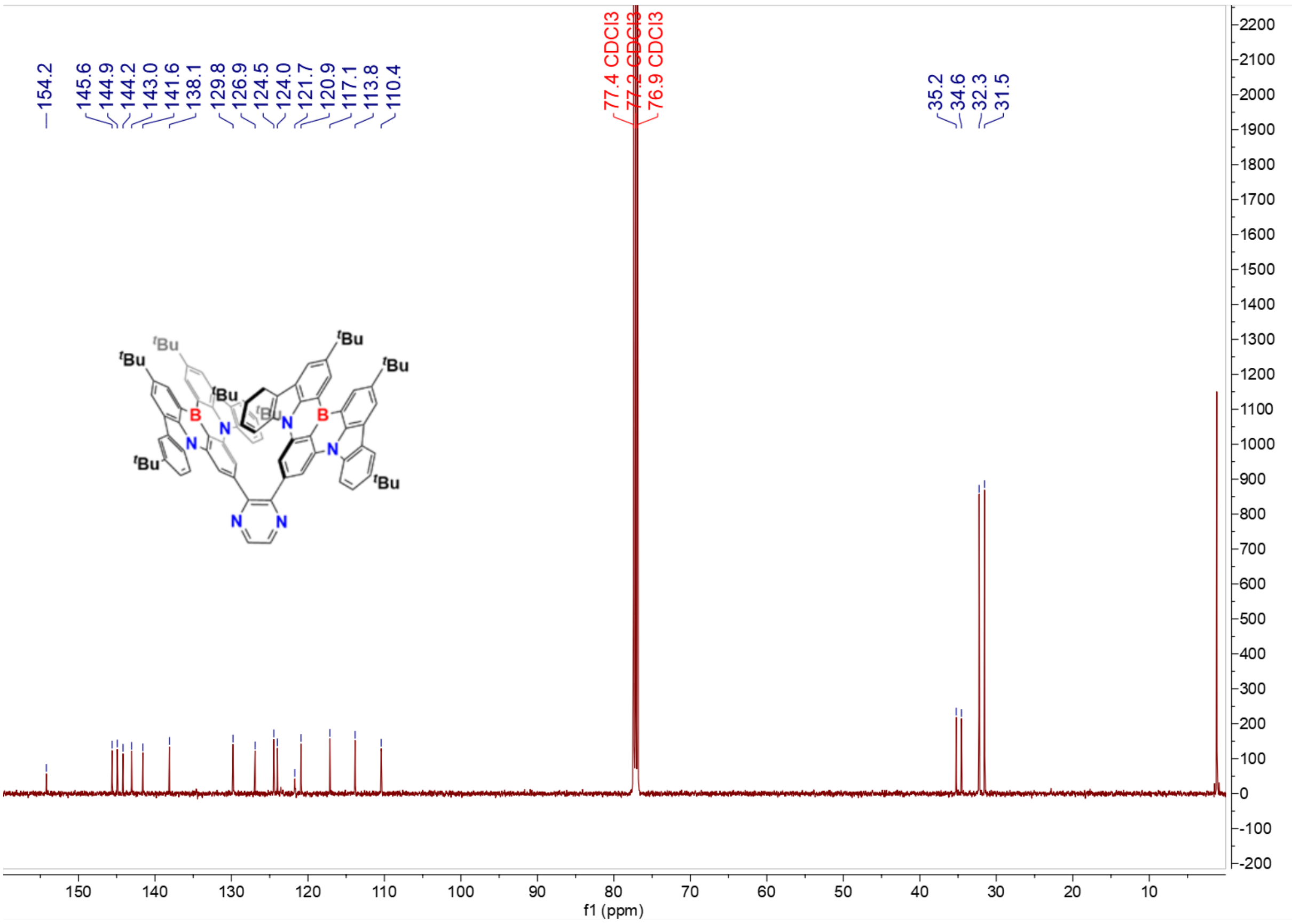

Supplementary Figure **13**. ^{13}C-NMR spectrum of **DDtBuCzB_Py** in $CDCl_3$ (126 MHz).

Synthesis of DDCzBPy

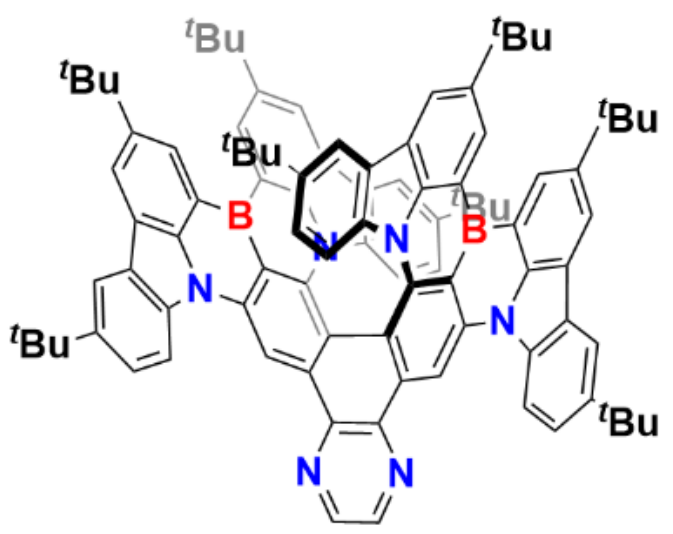

Under a N_2 atmosphere, **DDtBuCzB_Py** (500.0 mg, 0.37 mmol, 1.0 equiv.) and DDQ (100.5 mg, 0.44 mmol, 1.2 equiv.) were dissolved in 50 mL DCM. The solution was cooled to 0 °C, and TfOH (1 mL) was added dropwise. The reaction mixture was allowed to warm to room temperature and stirred for 1 hour. The reaction mixture was filtered through a celite pad and the insoluble residue was washed with dichloromethane (100 mL). The filtrates were combined; solvent was removed under reduced pressure and the residue was purified by silica gel flash column chromatography using DCM:hexane = 1:5 as eluent to afford the desired compound as a red solid. **Yield**: 74%. **Mp**: > 400 °C. **R$_f$**: 0.25 (DCM:hexane = 1:4). **^{1}H NMR (500 MHz, CD_2Cl_2) δ (ppm)**: 10.44 (s, 2H), 9.29 (d, *J* = 0.7 Hz, 2H), 9.05 (d, *J* = 8.8 Hz, 2H), 9.01 (d, *J* = 1.8 Hz, 2H), 8.84 (d, *J* = 1.8 Hz, 2H), 8.57 (d, *J* = 1.8 Hz, 2H), 8.41 (d, *J* = 2.0 Hz, 2H), 7.95 (dd, *J* = 8.7, 2.1 Hz, 2H), 7.84 (d, *J* = 1.8 Hz, 2H), 7.24 (d, *J* = 1.8 Hz, 2H), 5.95 (dd, *J* = 8.6, 2.0 Hz, 2H), 5.80 (dd, *J* = 8.5, 0.6 Hz, 2H), 1.77 (s, 18H), 1.69 (s, 18H), 1.62 (s, 18H), 0.76 (s, 18H). **^{13}C NMR (126 MHz, CD_2Cl_2) δ**

(ppm): 145.9, 145.1, 145.0, 145.0, 144.9, 143.9, 141.9, 141.4, 141.3, 139.0, 138.7, 136.1, 134.8, 130.6, 129.5, 127.5, 126.6, 125.2, 124.6, 123.8, 121.4, 120.9, 120.1, 117.7, 116.5, 115.0, 113.6, 112.4, 104.1, 35.5, 35.2, 34.3, 32.7, 32.4, 32.0, 31.3 **HR-MS [M+H]$^+$ Calculated:** ($C_{96}H_{97}B_2N_6$) 1355.8207; **Found:** 1355.8161. **EA calculated:** ($C_{96}H_{96}B_2N_6$) C, 85.07%; H, 7.14%; N, 6.20%. **Found:** C, 85.56%; H, 7.07%; N, 5.84%. **HPLC purity:** > 99% (retention time: 12.781 minutes and 17.403 minutes for ***P*-DDCzBPy** and ***M*-DDCzBPy**, respectively, employing an eluent consisting of DCM and hexane with a ratio of 1:4).

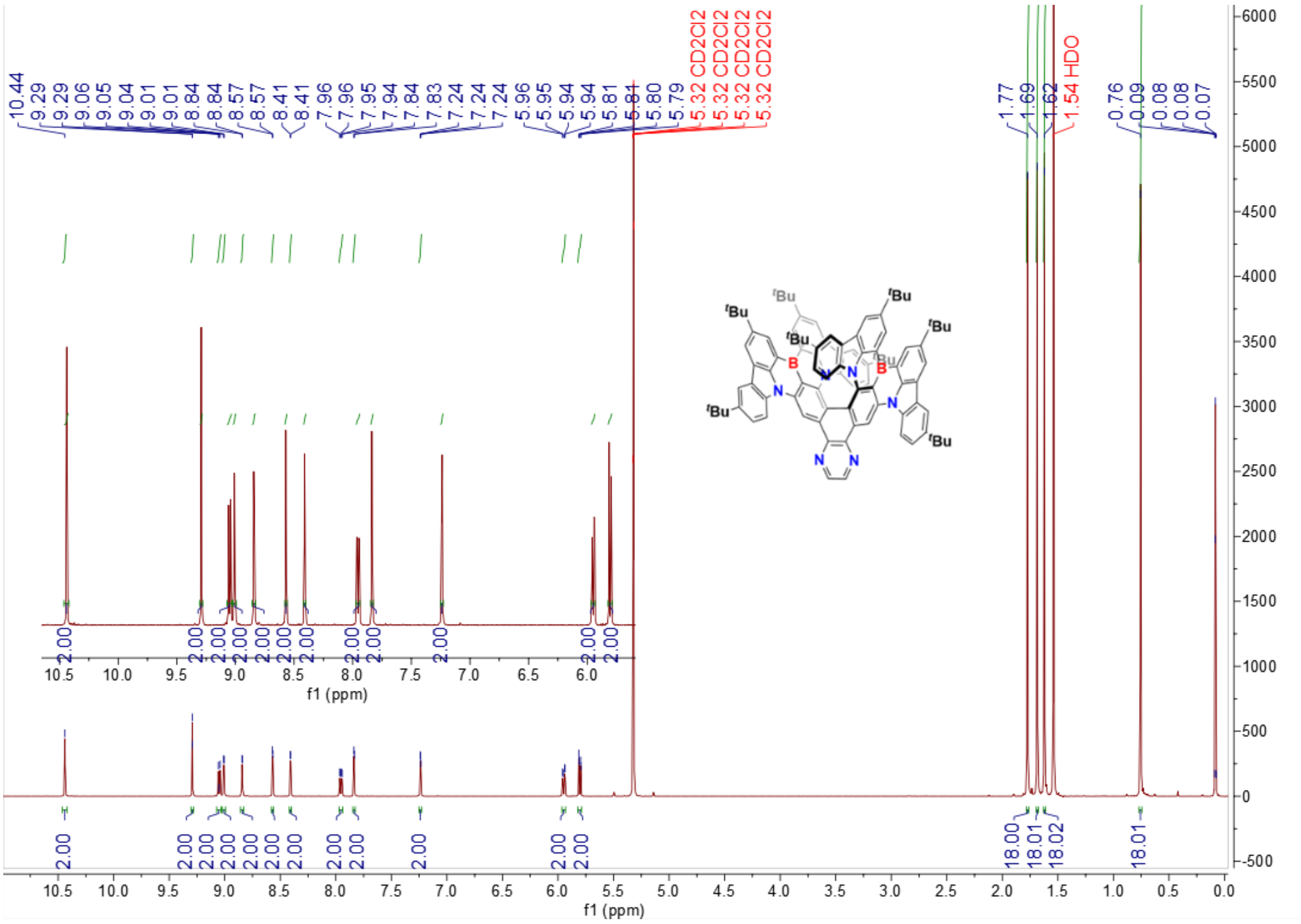

Supplementary Figure **14**. ^{1}H-NMR spectrum of **DDCzBPy** in CD_2Cl_2 (500 MHz).

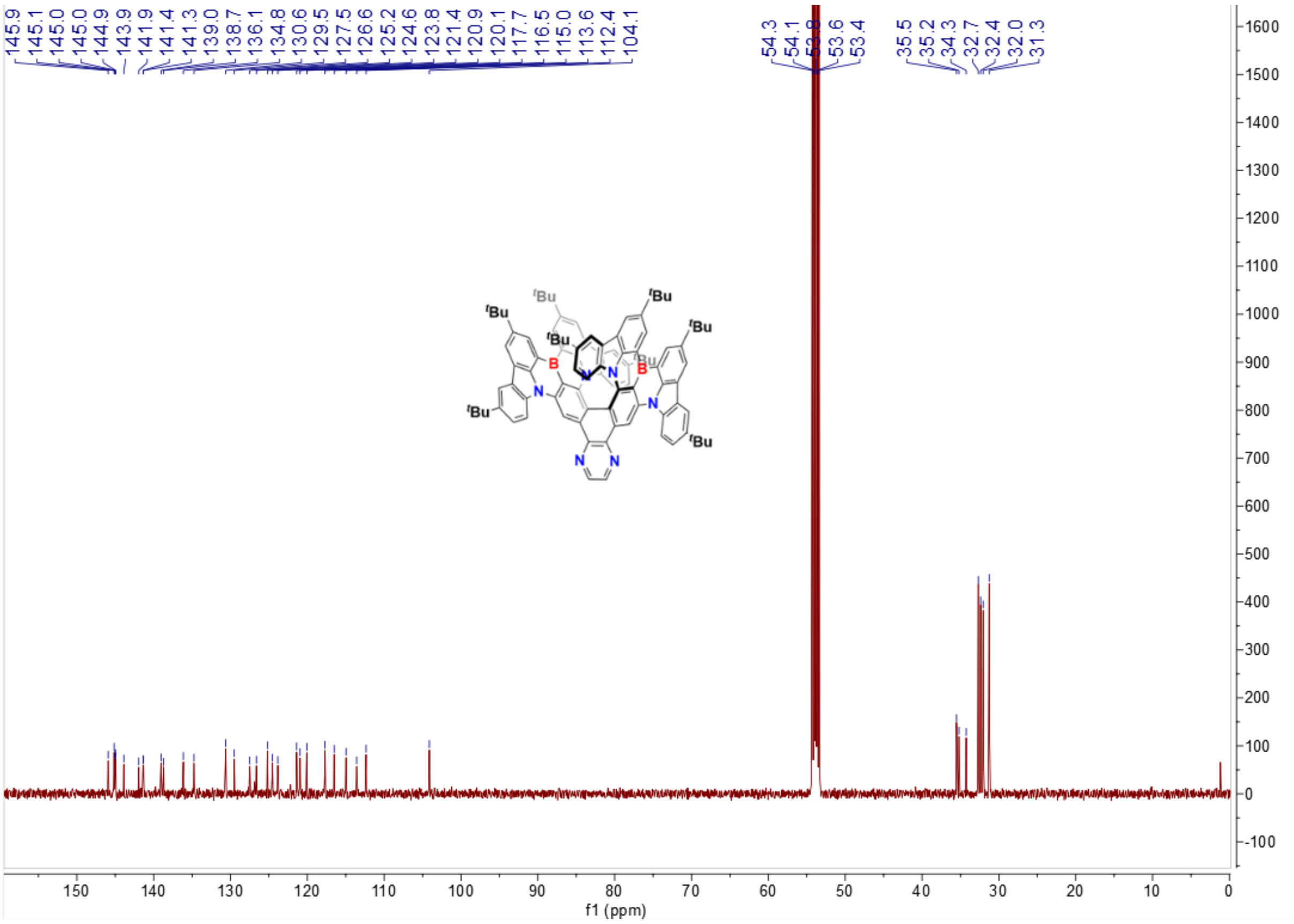

Supplementary Figure **15**. ^{13}C-NMR spectrum of **DDCzBPy** in CD_2Cl_2 (126 MHz).

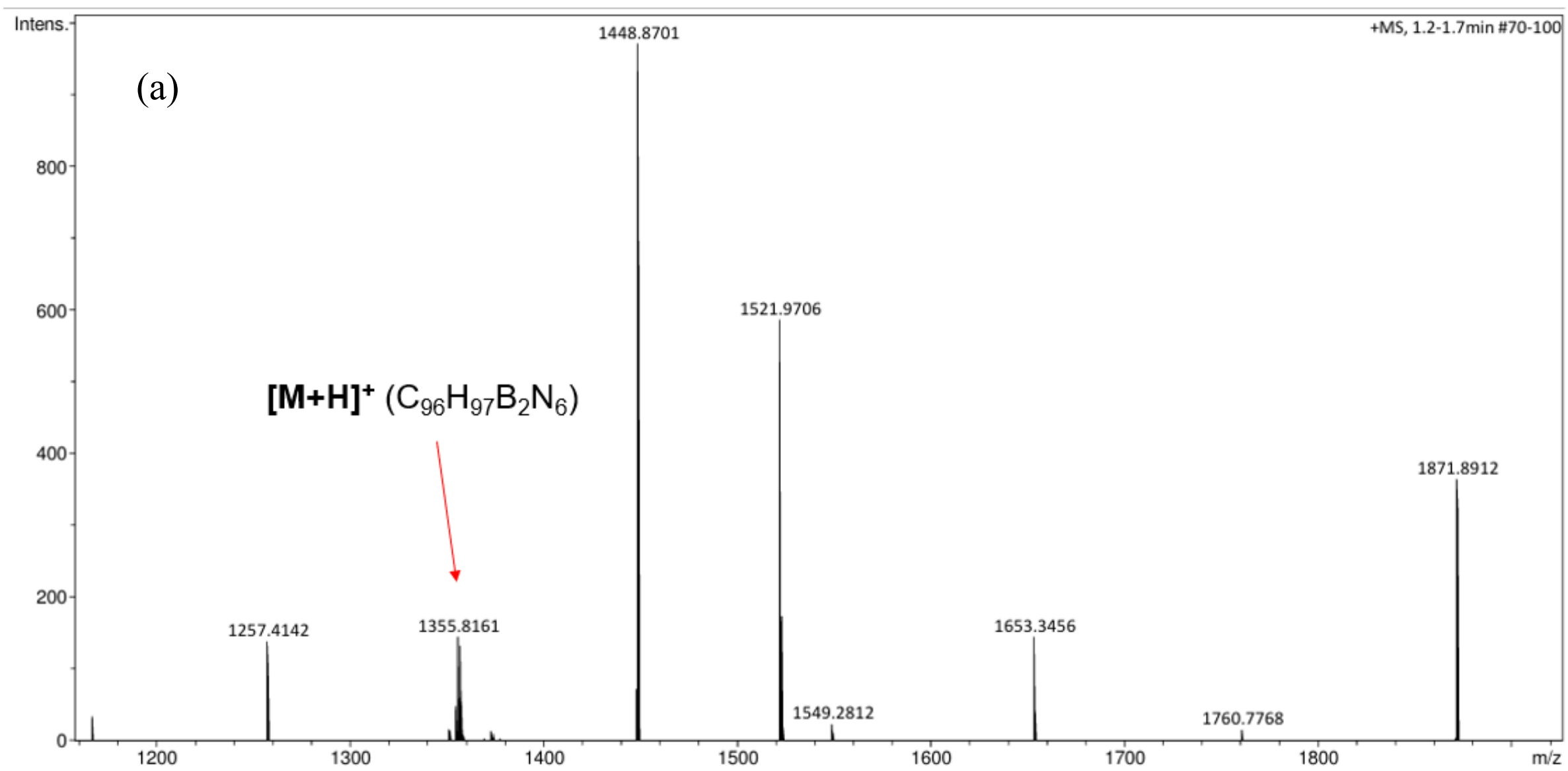

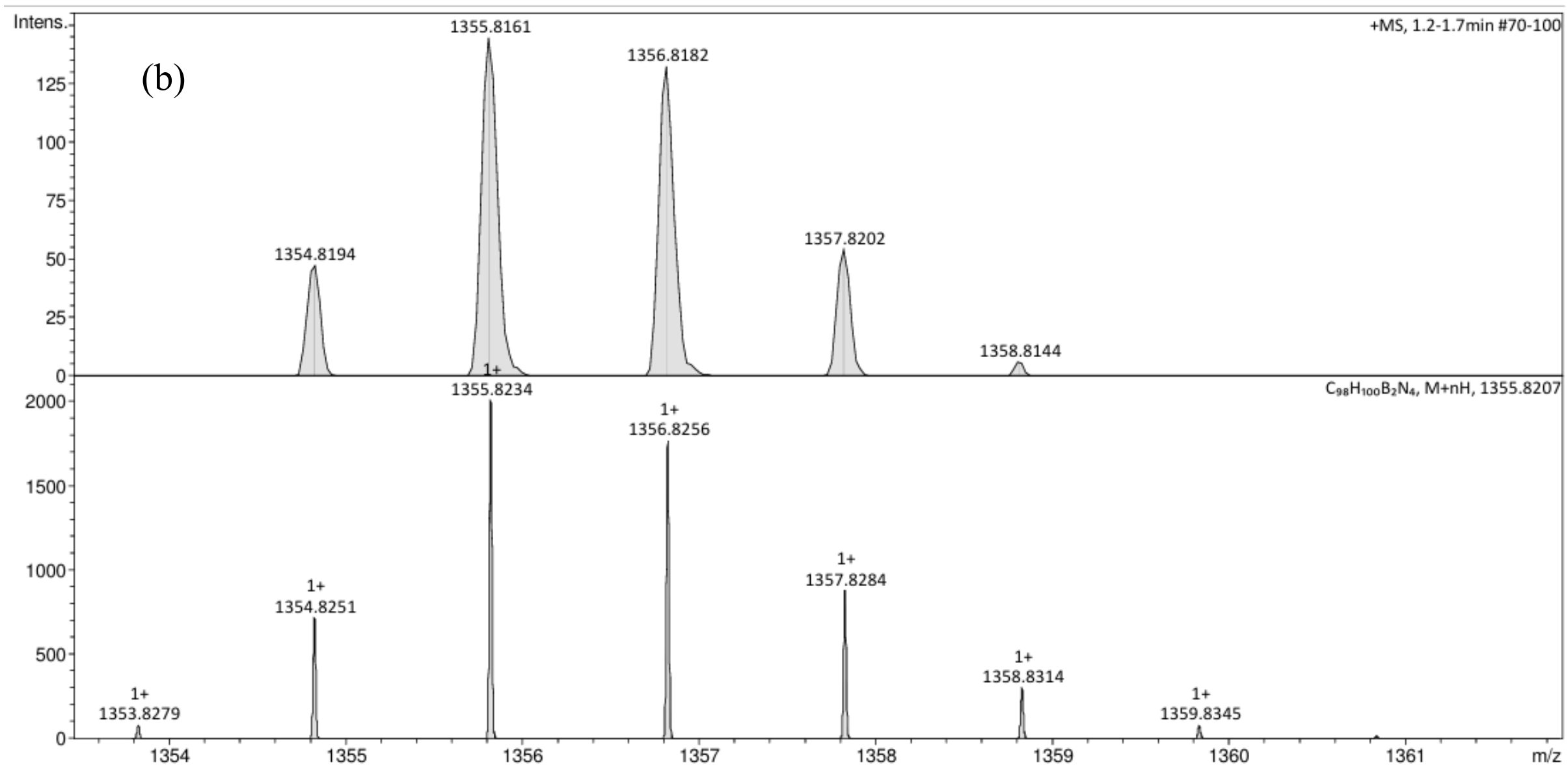

Supplementary Figure **16**. HRMS of **DDCzBPy** (a) wide range spectrum, (b) **[M+H]**$^+$ ($C_{96}H_{97}B_2N_6$)

<Chromatogram>

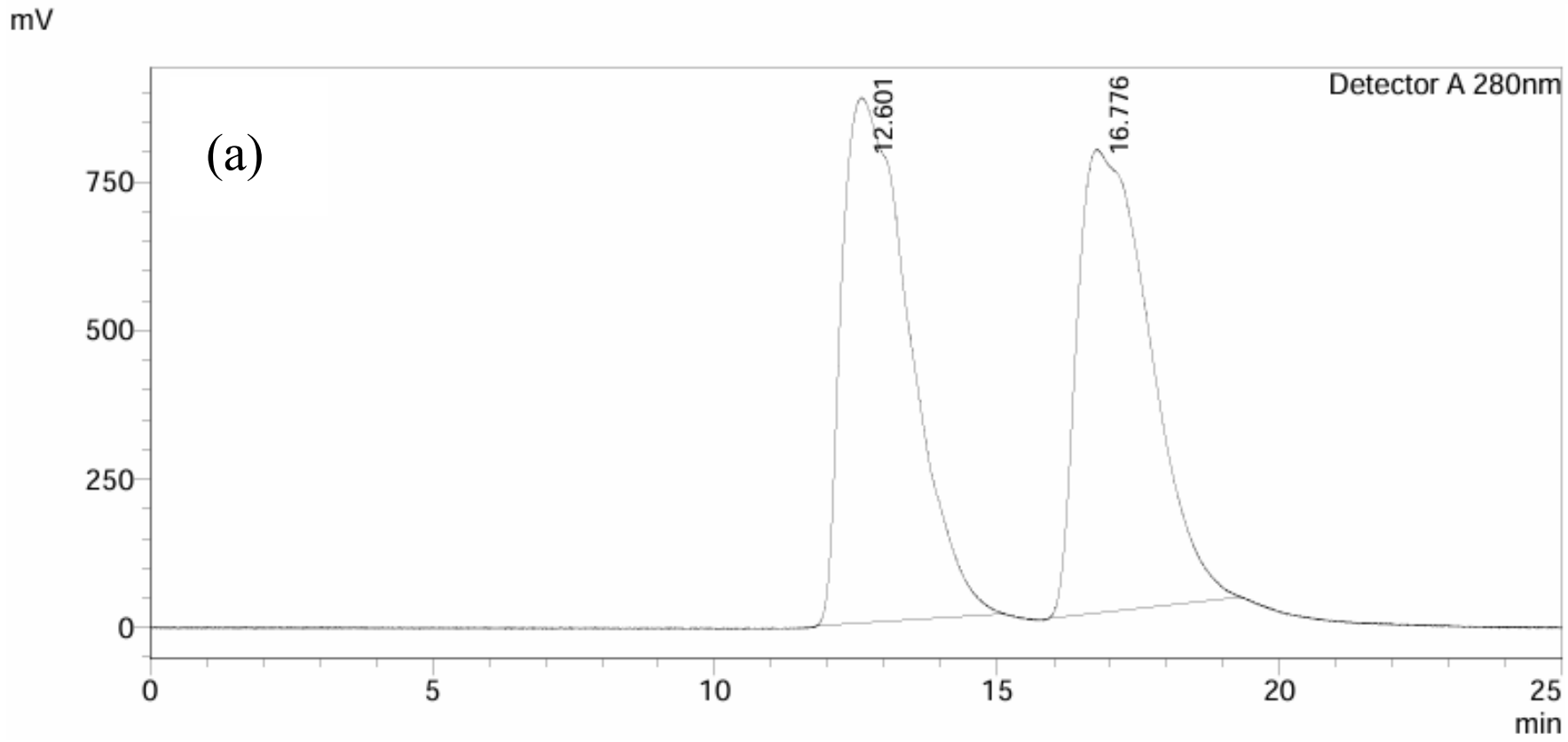

<Peak Table>

Detector A 280nm

Peak#	Ret. Time	Area	Height	Area%	Area/Height	Width at 5% Height
1	12.601	73490870	883612	51.226	83.171	2.564
2	16.776	69972859	779654	48.774	89.749	2.722
Total		143463729	1663266	100.000		

<Chromatogram>

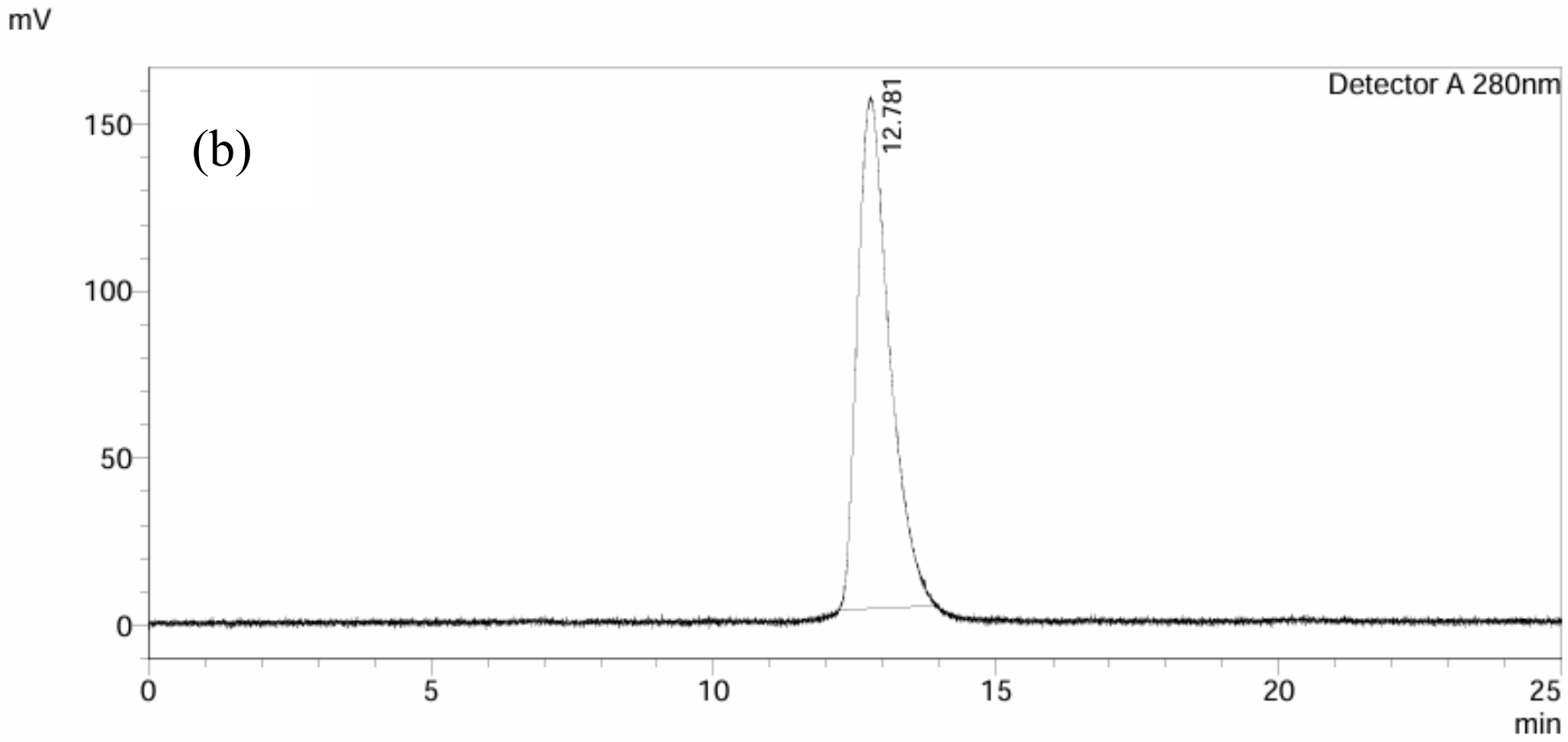

<Peak Table>

Detector A 280nm

Peak#	Ret. Time	Area	Height	Area%	Area/Height	Width at 5% Height
1	12.781	6029016	152754	100.000	39.469	1.350
Total		6029016	152754	100.000		

<Chromatogram>

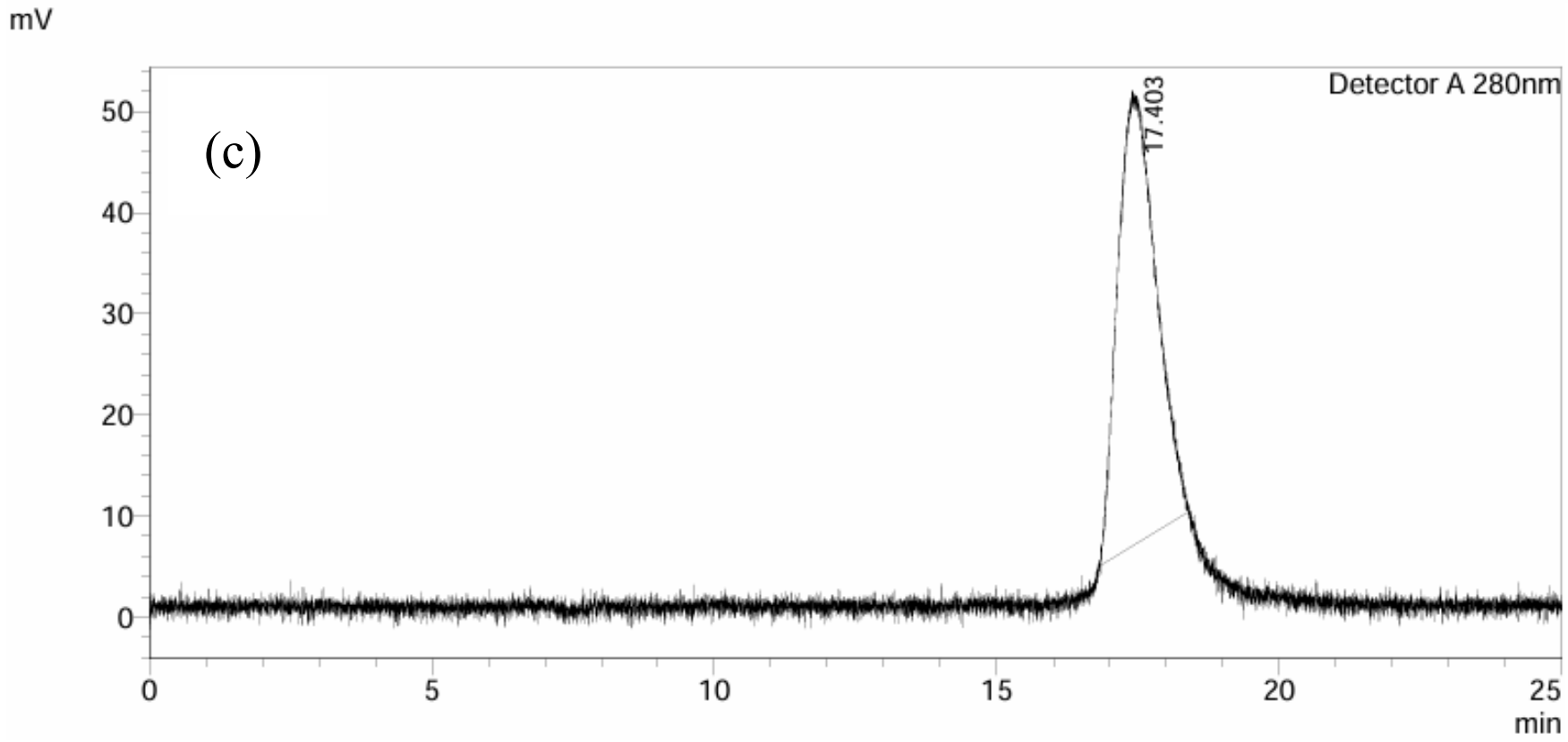

<Peak Table>

Detector A 280nm

Peak#	Ret. Time	Area	Height	Area%	Area/Height	Width at 5% Height
1	17.403	2117517	44549	100.000	47.532	1.401
Total		2117517	44549	100.000		

Supplementary Figure **17**. HPLC report of (a) (*rac*)-**DDCzBPy**, (b) ***P*-DDCzBPy** and (c) ***M*-DDCzBPy**.

LONDON METROPOLITAN UNIVERSITY
HUMAN SCIENCES

Elemental Analysis Sample Results

**Name**	Yan Xu
**Organisation Name**	University of St Andrews
**Purchase order number**	

Standard – Acetanilide		
**Element**	**Expected %**	**Found**
**Carbon**	71.10 (+/- 0.23)	71.33
**Hydrogen**	6.71 (+/- 0.07)	6.68
**Nitrogen**	10.34 (+/- 0.09)	10.42

Analysis – DDCzBPy			
**Element**	**Expected %**	**Found (1)**	**Found (2)**
**Carbon**	85.07	85.36	85.76
**Hydrogen**	7.14	7.02	7.11
**Nitrogen**	6.20	5.94	5.74

**Date completed**	18.11.2024
**Signature**	O. McCullough
**Comments**	

Supplementary Figure **18**. EA results of **DDCzBPy**.

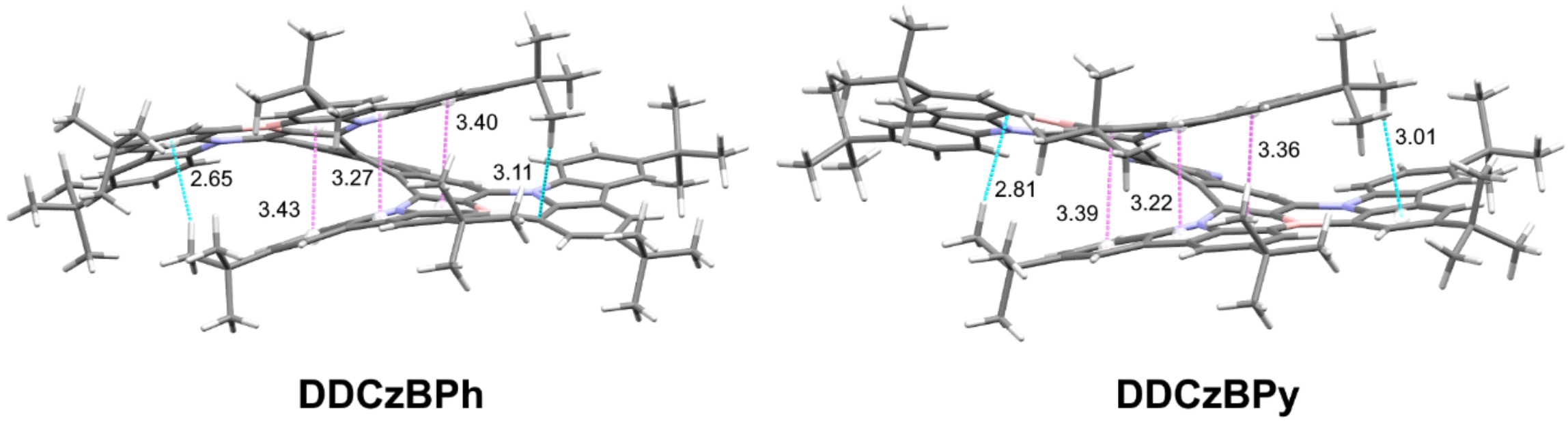

Supplementary Figure **19**. Single crystal structures. Intramolecular CH···π (light blue) and π···π (violet) interactions between two **DtBuCzB** planes (indicative distances in Å), with a single independent molecule shown, and minor components of disorder and solvents omitted for clarity in both structures.

4. Theoretical and optoelectronic data

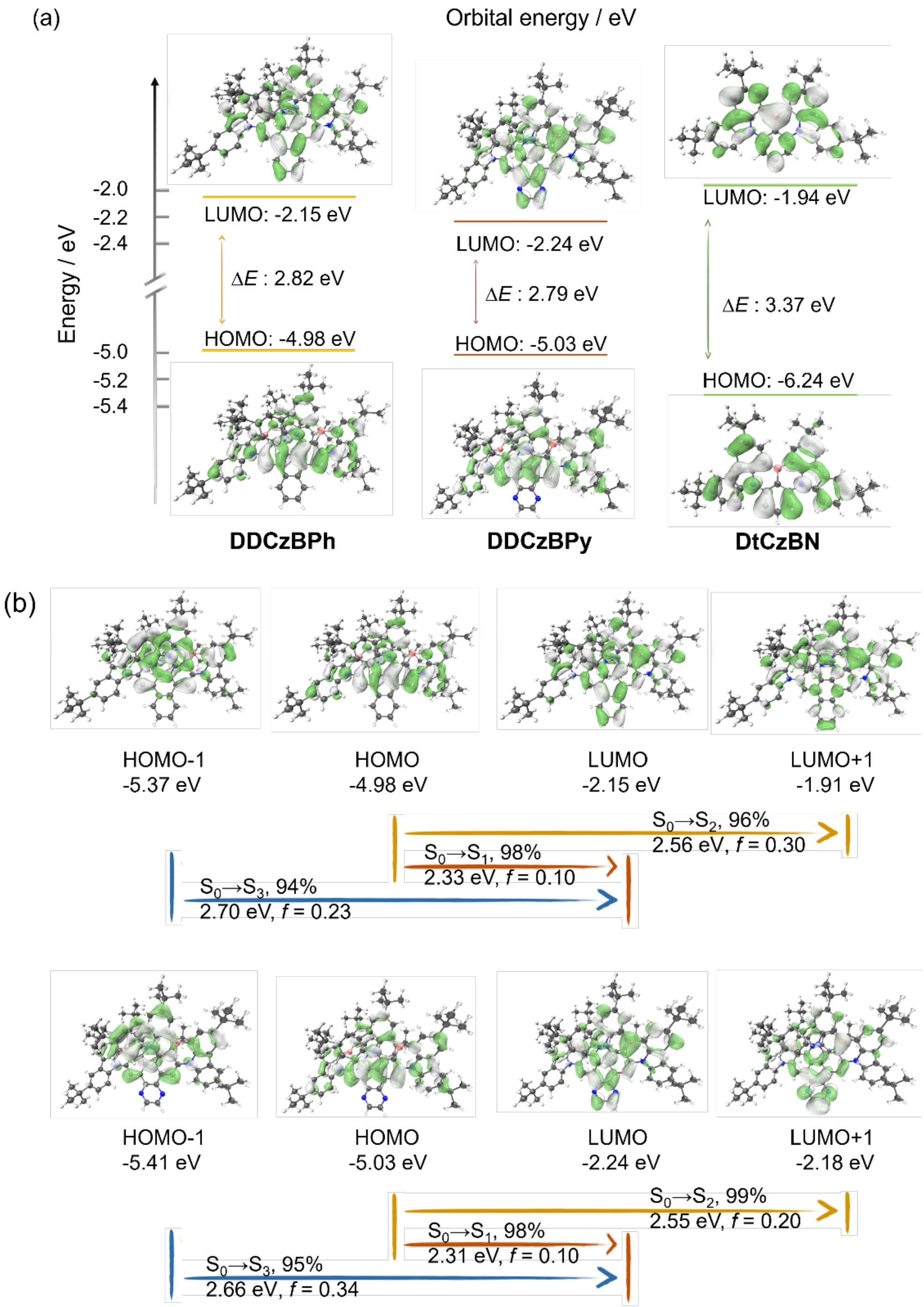

Supplementary Figure **20**. Theoretical modeling of **DDCzBPh** and **DDCzBPy** in the toluene solution at the B3LYP/def2-SVP level. (a) the energies and electron density distributions of the HOMO/LUMO (ISO value = 0.02); (b) the major molecular orbitals in the three lowest singlet states, along with the energy levels and contributions. *f* is the oscillator strength.

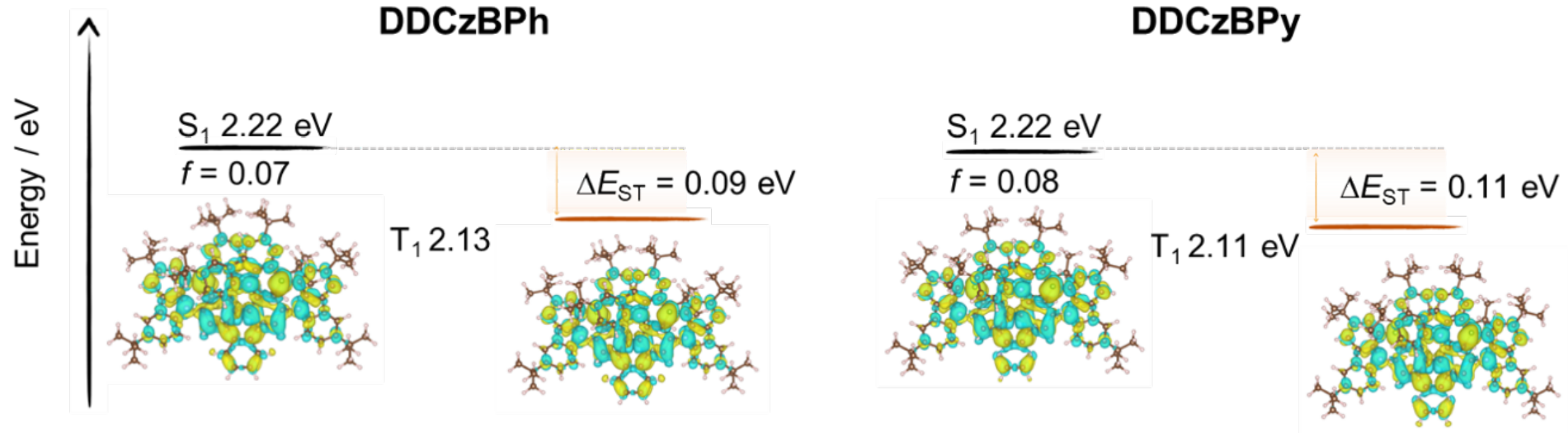

Supplementary Figure **21**. Difference density plots and energies for the S_1 and T_1 states of **DDCzBPh** and **DDCzBPy** calculated at the SCS-ADC2/cc-pVDZ level in the gas phase (ISO value = 0.02). The blue color represents an area of decreasing electron density, and the yellow color represents an increased electron density.

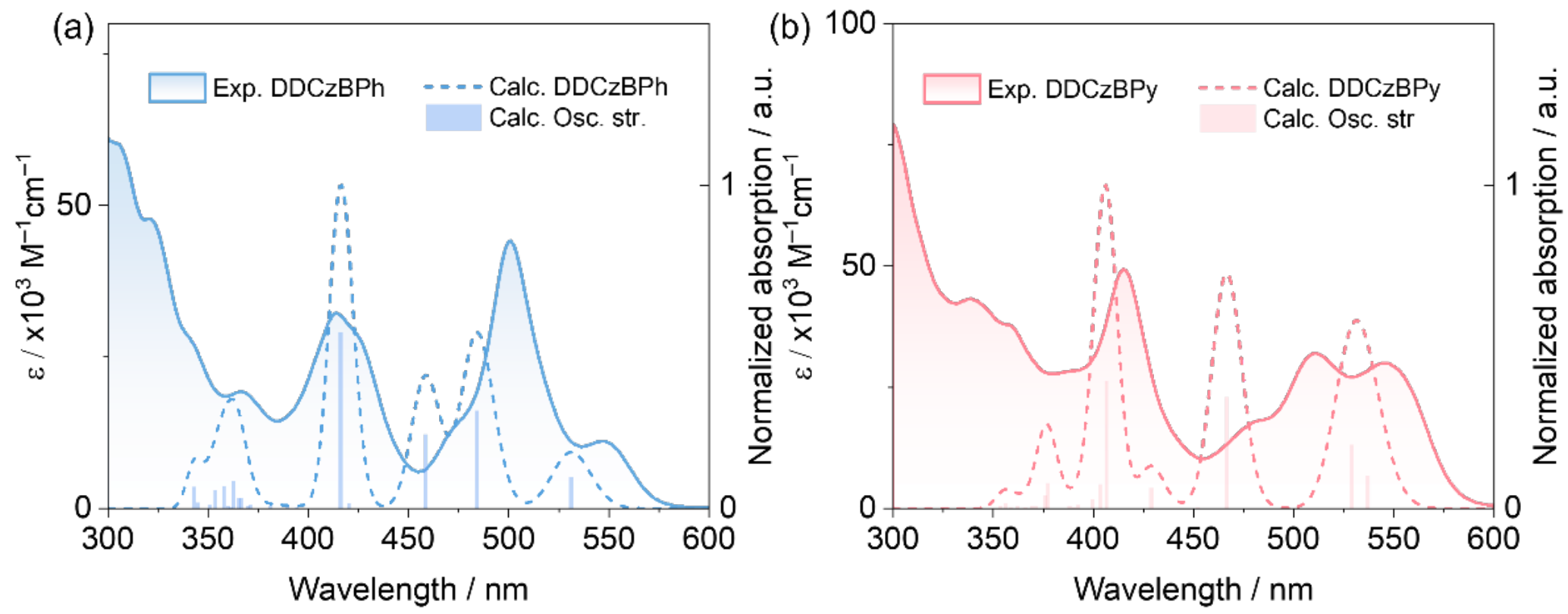

Supplementary Figure **22**. Simulated absorption spectra with a Gaussian function having FWHM of 0.05 eV (dashed line) and experimental absorption spectra in toluene (solid line) for (a) **DDCzBPh** and (b) **DDCzBPy**.

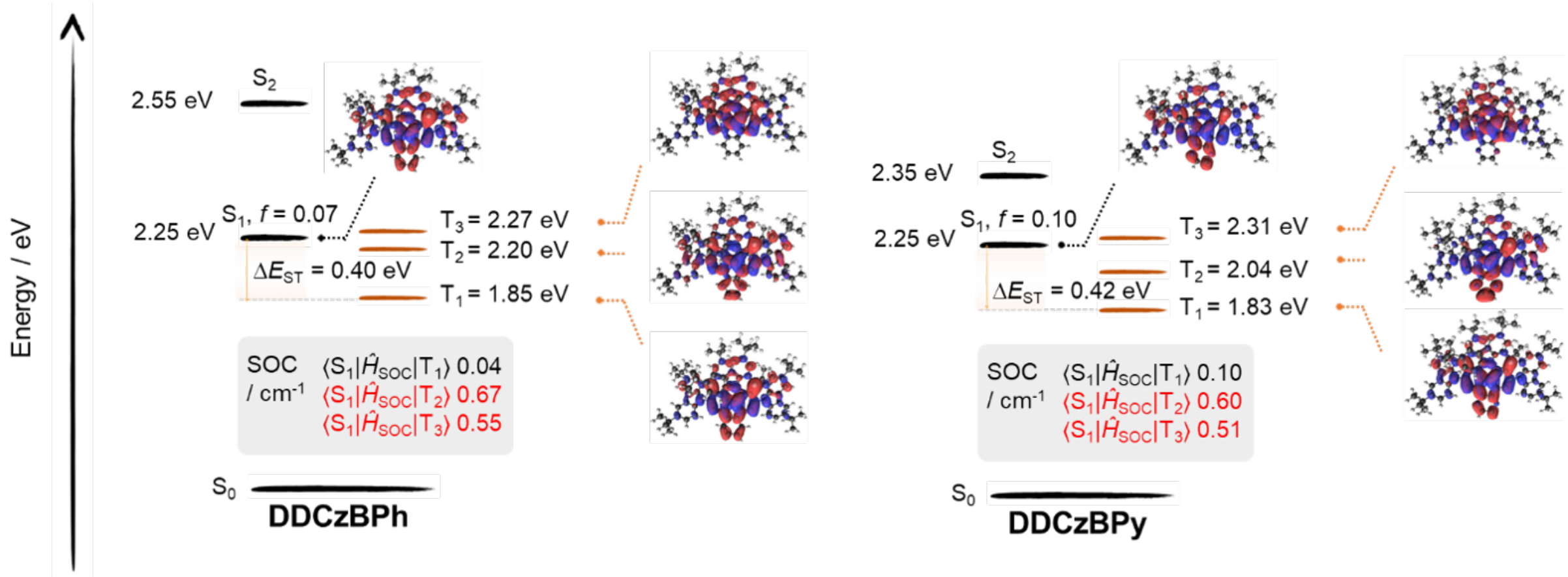

Supplementary Figure **23**. Theoretical calculation results for excited states. NTOs (particle and hole are represented by red and blue colors, respectively), the associated vertical excitation energies, SOC between the singlet state and triplet states are computed based on the optimized T_1 geometries at the

PBE0/6-31G(d,p) level in the gas phase for **DDCzBPh** and **DDCzBPy**. *f* is the oscillator strength.

Supplementary Table **2**. The summary of the calculated parameters for **DDCzBPh** and **DDCzBPy**.

**Transitions**	**Wavelength / nm**	***f*[a]**	**μ/ x 10^{-18} /esu·cm^{-1} [b]**	**m[h]/×10^{-21} erg·G^{-1} [c]**	**cos($\theta_{\mu,m}$)**	**$g_{lum/abs}$**
			**DDCzBPh**			
S_0-S_1	531	0.0968	3.31	6.88	-1.00	-0.008
S_0-S_2	484	0.3027	5.58	5.77	0.61	0.003
S_0-S_3	458	0.3027	4.72	1.00	0.97	0.001
S_1-S_0	593	0.0936	3.44	6.30	-0.99	-0.007
			**DDCzBPy**			
S_0-S_1	537	0.1006	3.39	6.42	-0.99	-0.008
S_0-S_2	529	0.1966	4.70	2.63	0.75	0.002
S_0-S_3	467	0.3435	5.84	1.40	0.82	0.001
S_1-S_0	597	0.0918	3.41	6.11	-0.99	-0.007

[a] Oscillator strength; [b] Electronic transition dipole moment; [c] Magnetic transition dipole moment; Calculated at the TD-DFT-B3LYP/def2-SVP level in toluene.

We estimated the energies of the frontier molecular orbitals (FMOs) using solution-state electrochemical methods. The oxidation and reduction potentials (E_{ox}/E_{red}) were investigated by cyclic voltammetry (CV) and differential pulse voltammetry (DPV) in deaerated DCM (Supplementary Figure **23**, Table **3**). **DDCzBPh** shows reversible oxidation and reduction waves at 0.70 and -1.65 V vs SCE. Compared to the parent **DtCzBCz** (1.03/-1.73 V),[2] the oxidation potential is cathodically shifted, while the reduction potential is slightly anodically shifted. This indicates that there is enhanced conjugation in **DDCzBPh**, resulting in destabilized HOMO and stabilized LUMO energies, and narrower ΔE_{H-L} of -5.04/-2.69 eV and 2.35 eV for **DDCzBPh**, compared to -5.37/-2.61 eV and 2.76 eV for **DtCzBCz**. These calculated HOMO/LUMO energy levels for **DDCzBPh** are much more destabilized than those previously reported (-5.91/-3.20 eV).[1] After replacing the bridging unit from naphthalene in **DDCzBPh** to quinoxaline in **DDCzBPy**, both the oxidation and reduction potentials shift anodally to 0.77 V and -1.52 V, respectively, owing to the electron-withdrawing character of the nitrogen-containing bridging unit. This results in slightly stabilized HOMO/LUMO energy levels and narrower ΔE_{H-L} of -5.11/2.83 eV and 2.28 eV for **DDCzBPy**. The trends in the ΔE_{H-L} inferred from the electrochemical data largely mirror those from the DFT study.

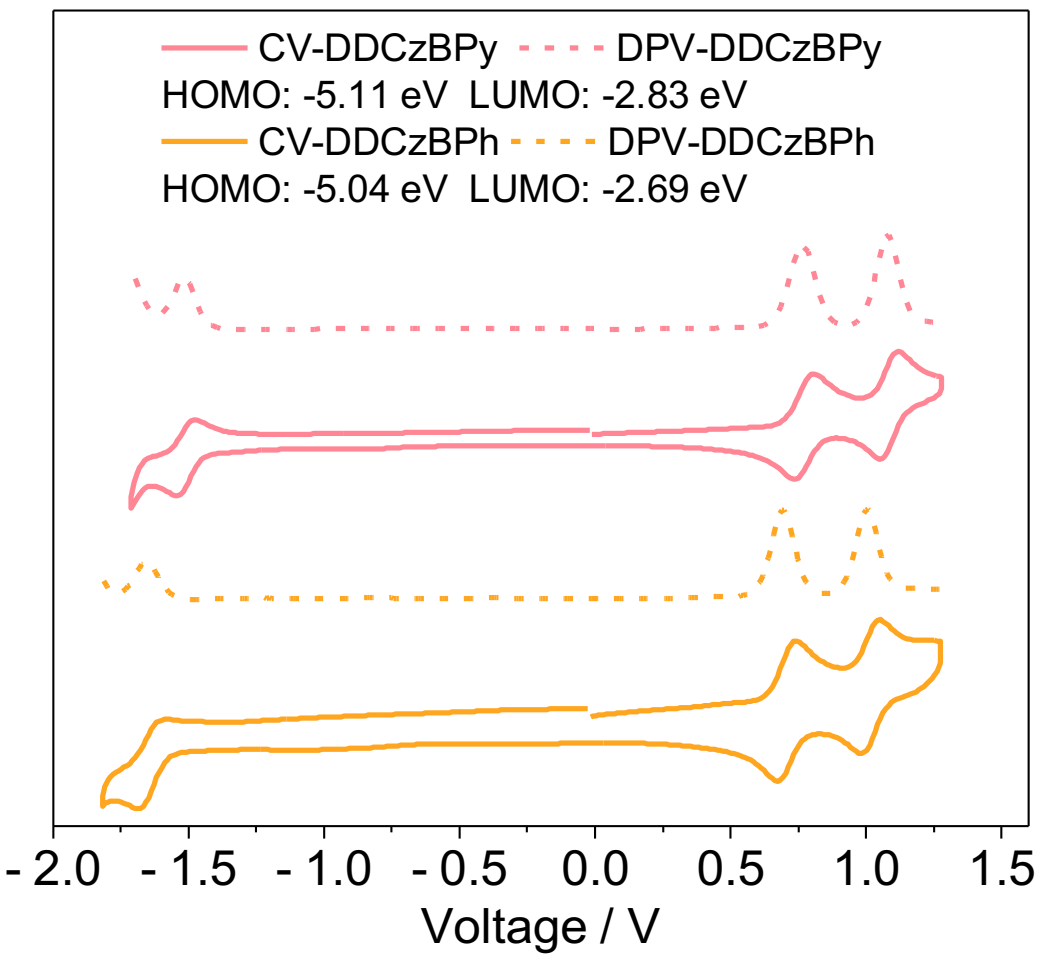

Supplementary Figure **24**. CVs and DPVs of **DDCzBPh** and **DDCzBPy** in deaerated DCM with 0.1 M [^{n}Bu$_4$N]PF$_6$ as the supporting electrolyte and Fc/Fc$^+$ as the internal reference (0.46 V vs. SCE), scan rate 100 mV/s.[3]

Supplementary Table **3**. Electrochemical data.

	E_{ox} [a] / v *vs* SCE	E_{red} [a] / v *vs* SCE	HOMO[b] / eV	LUMO[b] / eV	ΔE_{redox} [c] / eV
**DDCzBPh**	0.70	-1.65	-5.04	-2.69	2.35
**DDCzBPy**	0.77	-1.52	-5.11	-2.83	2.28

[a] Measured in DCM (0.46 V for DCM *vs* SCE) with a scan rate of 0.1 V/s. [b] The HOMO and LUMO energies were determined using $E_{HOMO/LUMO} = -(E_{ox}/E_{red}+4.8)$ eV[4] where E_{ox} and E_{red} are anodic and cathodic peak potentials, respectively, versus Fc/Fc^+. [c] $\Delta E_{redox} = |E_{HOMO}-E_{LUMO}|$.

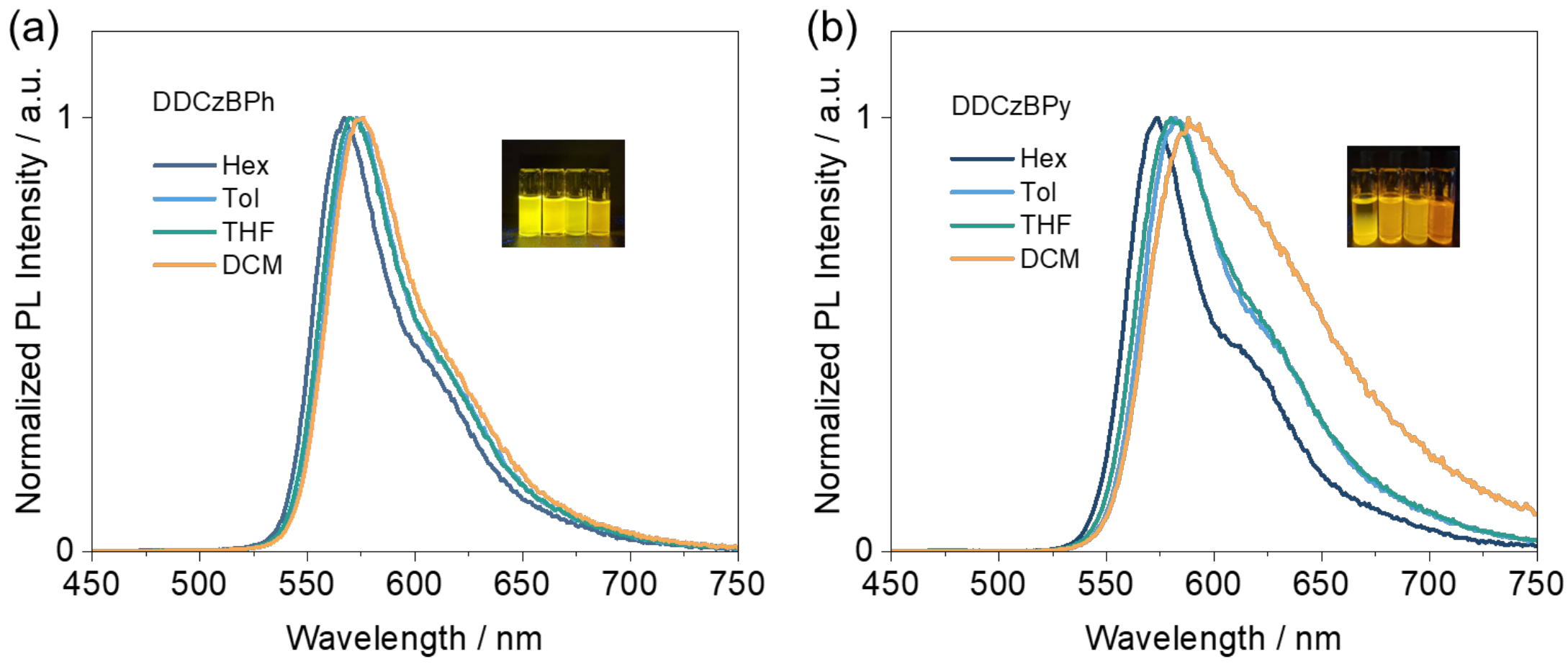

Supplementary Figure **25**. Steady-state PL spectra in different solvents. (a) **DDCzBPh** and (b) **DDCzBPy**. (λ_{exc} = 340 nm).

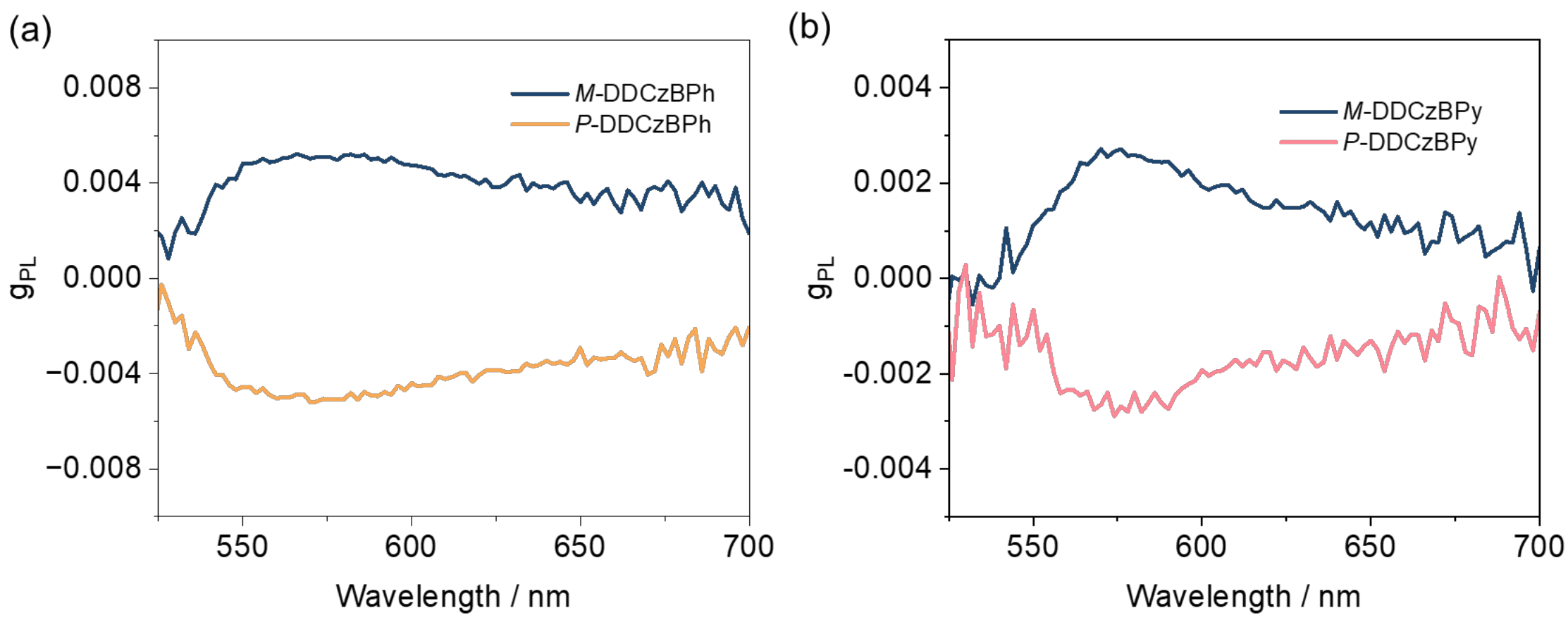

Supplementary Figure **26**. g_{PL} measured in toluene under 1PE. (a) **DDCzBPh** and (b) **DDCzBPy** (λ_{exc} = 416 nm).

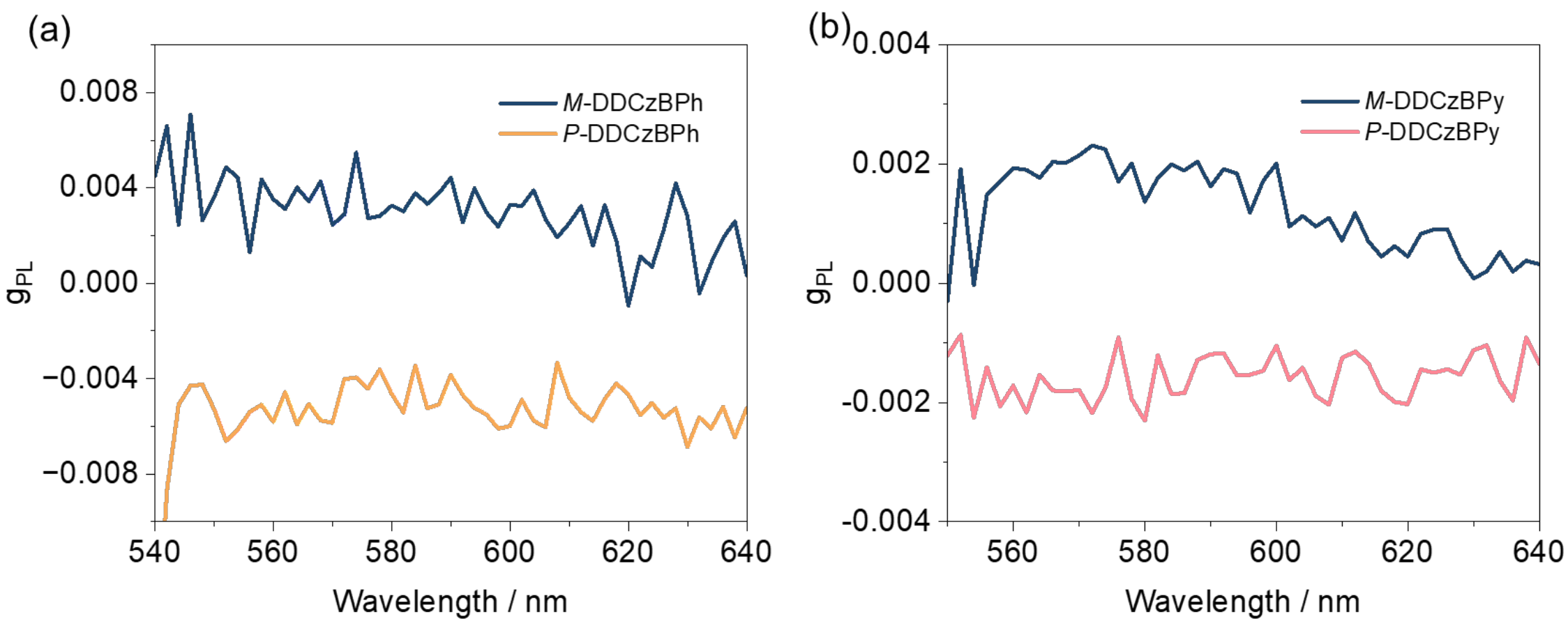

Supplementary Figure **27**. g_{PL} measured in toluene under 2PE. (a) **DDCzBPh** and (b) **DDCzBPy** (λ_{exc} = 780 nm).

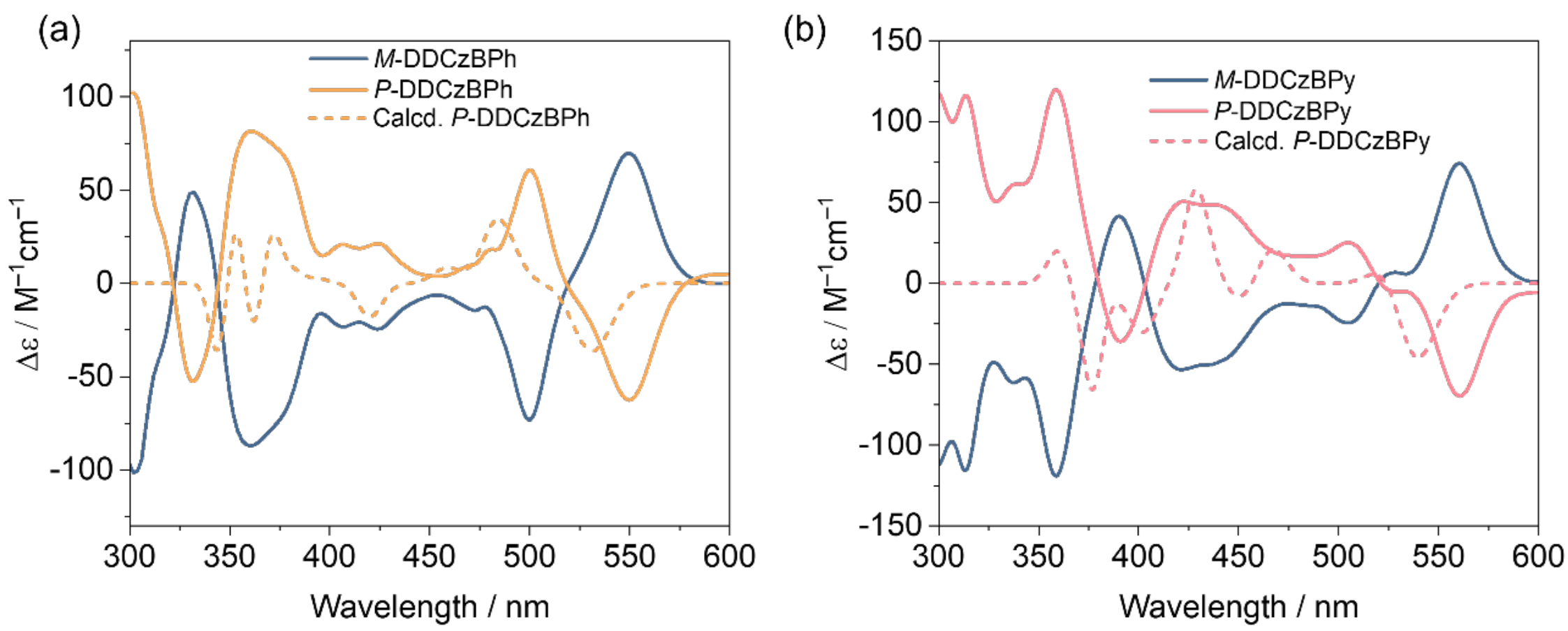

Supplementary Figure **28**. Experimental and calculated ECD spectra. (a) ***P-/M*-DDCzBPh** and (b) ***P-/M*-DDCzBPy** measured in toluene. Predicted ECD spectra were calculated at the B3LYP/def2-SVP with an implicit toluene solvent field (IEFPCM).

5. Kinetics parameters

The kinetics parameters were calculated according to the following equations and summarized in Table **S3**.[5]

$\Phi_{PL} = \Phi_p + \Phi_d$ (1)

$k_p = \frac{1}{\tau_p}$ (2)

$k_d = \frac{1}{\tau_d}$ (3)

$k_r^S = k_p \Phi_p$ (4)

$k_{ISC} = k_p(1\text{-}\Phi_p)$ (5)

$k_{RISC} = \frac{k_p k_d}{k_{ISC}} \frac{\Phi_d}{\Phi_p}$ (6)

$k_{nr}^T = k_d\text{-}\Phi_p k_{RISC}$ (7)

where the Φ_p and Φ_d are the prompt fluorescent and delayed fluorescent quantum efficiencies, respectively. The overall photoluminescence quantum efficiency was measured using the optically dilute method in toluene mentioned previously, and the Φ_p and Φ_d were determined as the fraction of prompt and delayed emission based on the ratio of prompt and delayed components obtained from the integration of their time-resolved PL decays; k_p is the rate constant of prompt fluorescence; k_d is the rate constant of delayed fluorescence;

$k^T{}_{nr}$ is the non-radiative decay rate constant of T_1; $k^S{}_r$ is the radiative decay rate constant of S_1; k_{ISC} is the intersystem crossing rate constant; k_{RISC} is the reverse intersystem crossing rate constant.

Supplementary Table **4**. Summary of kinetics parameters for **DDCzBPh** and **DDCzBPy** in degassed toluene

	Φ_p / %	Φ_d / %	k_p / 10^7 s^{-1}	k_d / 10^4 s^{-1}	$k^T{}_{nr}$ / 10^3 s^{-1}	$k^S{}_r$ / 10^7 s^{-1}	k_{ISC} / 10^7 s^{-1}	k_{RISC} / 10^4 s^{-1}
**DDCzBPh**	30	35	8.97	1.54	7.75	2.73	6.24	2.51
**DDCzBPy**	41	39	8.47	0.91	3.12	3.51	4.96	1.45

Supplementary Table **5**. Summary of key parameters for 1PE CPL brightness in toluene.

Compound	$\|g_{PL}\|$ / 10^{-3}	ε / 10^3 M^{-1} cm^{-1}	Φ_{total} / %	1PE-CPB / M^{-1} cm^{-1}
***P*-DDCzBPh**	5.2	44.09	65	74.51
***M*-DDCzBPh**	5.2	44.09	65	74.51
***P*-DDCzBPy**	2.9	29.92	80	34.71
***M*-DDCzBPy**	2.7	29.92	80	32.31
***P*-DDCzBPh**[1]	5.5	77.8	98	209.70
***M*-DDCzBPh**[1]	5.8	77.8	98	220.75

Supplementary Table **6**. Summary of key parameters for 2PE CPL brightness in toluene.

Compound	$\|g_{PL}\|$ / 10^{-3}	σ^2 / GM	Φ_{total} / %	2PE-CPB[a] / GM
***P*-DDCzBPh**	5.2	116	65	0.20
***M*-DDCzBPh**	5.2	116	65	0.20
***P*-DDCzBPy**	2.9	138	80	0.16
***M*-DDCzBPy**	2.7	138	80	0.15

[a] 2PE-CPB = $\sigma^2 \cdot \Phi_{PL} \cdot (1PE\text{-}g_{PL}/2)$

References

[1] W. C. Guo, W. L. Zhao, K. K. Tan, M. Li, C. F. Chen, *Angew. Chem. Int. Ed. Engl.* **2024**, *63*, e202401835.

[2] S. Wu, D. Chen, X. H. Zhang, D. Sun, E. Zysman-Colman, *Adv. Mater.* **2025**, *37*, e2415289.

[3] N. G. Connelly, W. E. Geiger, *Chem. Rev.* **1996**, *96*, 877-910.

[4] C. M. Cardona, W. Li, A. E. Kaifer, D. Stockdale, G. C. Bazan, *Adv. Mater.* **2011**, *23*, 2367-2371.

[5] (a) K. Masui, H. Nakanotani, C. Adachi, *Org. Electron.* **2013**, *14*, 2721-2726; (b) Y. Tsuchiya, S. Diesing, F. Bencheikh, Y. Wada, P. L. Dos Santos, H. Kaji, E. Zysman-Colman, I. D. W. Samuel, C. Adachi, *J. Phys. Chem. A* **2021**, *125*, 8074-8089.
