# Supplementary material for: Two-photon excited circularly polarized luminescence of hetero[9]helicene with multiresonant thermally activated delayed fluorescence
Source: Nat Commun. 2026 Sep 15;17:10814. doi: 10.1038/s41467-026-77554-5 (PMC13669335; doi:10.1038/s41467-026-77554-5)
Supplement: Supplementary file 2 [file 41467_2026_77554_MOESM2_ESM.pdf]

nature portfolio

Peer Review File

Two-Photon Excited Circularly Polarized Luminescence of Hetero[9]helicene with Multi-resonant Thermally Activated Delayed Fluorescence

Corresponding Author: Professor Eli Zysman-Colman

This file contains all reviewer reports in order by version, followed by all author rebuttals in order by version.

Version 0:

Reviewer comments:

Reviewer #1

(Remarks to the Author)
In this paper the authors report two chiral hetero[9]helicene MR-TADF molecules, consisting of two annulated MR-TADF units to a central naphthyl or quinoxanyl group. The two pairs of CP-MR-TADF enantiomers show strong CPL, two-photon absorption, high photoluminescence quantum yields, and long-lived delayed fluorescence. This paper reports some interesting results because there are only few MR-TADF molecules showing the 2PA properties. Especially, the authors think they demonstrate for the first time two-photon CPL of TADF emitters, with their circularly polarized brightness above 0.1 GM, demonstrating their potential for CPL biological imaging. But there are also some problems for this work.
1. Though the authors report the first time two-photon CPL of TADF emitters, the chiral hetero[9]helicene MR-TADF molecule DDCzBPh has been reported by Chen et al. (Angew. Chem. Int. Ed. 2024, 63, e202401835), as they commented in the text. Another one has very similar molecular structure. The synthesis method, crystal determination, photophysical and chiroptical (including the theoretical calculation for electronic, magnetic transition dipole moments) properties are consistent with literature reports. Therefore, the novelty of this research is not very high. The authors believe these materials have potential for CPL biological imaging, but there is no corresponding results to support this point. I noticed that the authors added the sentence "This is supported by a prior report in which (S)-BOD1, with a small |gPL| of 6×10-4, was used as a CPL probe in living cells using CPL-laser scanning confocal microscopy (CPL-LSCM)." in the text, but it is not enough. The results and advantages of these CP-MR-TADF enantiomers for CPL biological imaging are necessary.
2. In the Introduction section, the authors gave one example of functionalized cationic chiral diaza[4]helicenes, which showed one photon exciton (1PE) gPL values of +5.8/–6.7×10–4 and 2PE gPL values of +9.4/–9.2×10–4. It seems that the 2PE gPL values are higher than that of 1PE gPL data. But in this case, the 2PE gPL values are a little lower than that of 1PE gPL data. The authors should explain this difference from literatures.
3. I think the position of Figure 3 is not suitable. In the "Theoretical calculations" section the authors did not mention this figure, but discussed the corresponding results after Figure 5.
4. Furthermore, the authors also should pay attention to their writings. For example,
In the title, the authors used "Multiresonant", but in the Abstract and text they used "Multi-resonant"; and "Multi-resonant" is an adjective word, which is not suitable as a Keyword;
" F" and " PL"
In "To date, dozens of CP-MR-TADF emitters have been reported.", the full name of "CP-MR-TADF" should be provided;
"two-photon excitation (2PE)" appears twice

Reviewer #2

(Remarks to the Author)
The manuscript by Pal, Zysman-Colman, and co-workers describes two hetero[7]helicenes exhibiting two-photon (2P) CPL. In light of the limited number of prior reports in this area, and the good–though not exceptional–2P-CPL performance of the present compounds, I believe the work has sufficient significance and novelty to deserve publication in Nature Communications. This holds true even though one of the two compounds has been previously reported, along with its chiroptical properties, although without evaluation of its 2P-CPL activity.

My main concerns relate to the theoretical and computational rationalization of the (chiro)optical properties. The calculations were performed at the PBE0/6-31G(d) level of theory. In a recent review I co-authored on CPL calculations (Chem. Rev., 2025, 125, 10492–10656), we collected extensive literature data on CPL simulations for helicenes. Our survey highlights, among other aspects, the strong dependence of computed results on the choice of functional and basis set. In the present case, the selected functional may not adequately describe the charge-transfer character of the key electronic transitions, while the basis set is too limited in size to provide quantitatively reliable results. Although computational analysis is not the main topic of the manuscript, a more proper choice of the functional and basis set may possibly solve a few inconsistencies listed below.

1) Although the two compounds share the same ground-state symmetry (C_2), the symmetry of the $S_1 \rightarrow S_0$ transition appears to differ: the transition dipole moment is oriented parallel to the C_2 axis in DDCzBPh and orthogonal to it in DDCzBPy. The origin of this discrepancy is not addressed by the authors. A plausible explanation is excited-state desymmetrization in S_1, a phenomenon that is particularly relevant for CPL emitters (see review cited above) and deserves a more in-depth analysis.
2) The authors stress a different degree of CT character in the two molecules, which they deem responsible for the discrepancy in gPL values. However, frontier molecular orbitals, transition density plots and NTO's provided in the Supporting Information look extremely similar for the two compounds. One must conclude that the current level of theory is not able to describe properly the ICT state.
3) In line with previous point, the calculated absorption spectra (Fig. S20) do not at all "match well the experimental ones". The shape and relative band intensities are not reproduced, and some bands are apparently missing (including the 1st experimental band for DDCzBPh?)
4) Since the calculation of the absorption profile also provides calculated ECD spectra, as a quality check the authors are asked to compare experimental and calculated ECD spectra, as well.

Summarizing, the authors are kindly asked to address the following points:
(a) Re-run the calculations using a higher level of theory, for example CAM-B3LYP/def2-TZVP, including an appropriate solvent model.
(b) Provide updated comparisons between calculated and experimental absorption *and* ECD spectra.
(c) Reassess the nature of the $S_1 \rightarrow S_0$ transition.
(d) Discuss the (possible) desymmetrization of the S_1 excited state.
(e) If warranted, include a quantitative evaluation of the charge-transfer character using one of the established metrics available in the literature.

From an experimental perspective, I note that the two-photon excitation spectra lack long-wavelength bands. Could the laser excitation range be extended to probe these regions as well?

Finally, a more critical comparison between the data reported for DDCzBPh and those from the previous study (ref. 13) would be useful. While most parameters are in good agreement, this is not the case for CPB, where the reported values differ by approximately a factor of 3.

Reviewer #3

(Remarks to the Author)
In this manuscript, the authors report two-photon-excited circularly polarized luminescence (2PE-CPL) from MR-TADF emitters. The experimental work is of high quality: the synthesis, chiral resolution, and comprehensive photophysical and chiroptical characterization are carefully executed and convincingly presented.

With respect to novelty and positioning within the literature, the principal advance is the first demonstration of 2PE-CPL in an MR-TADF molecular system. However, one of the investigated molecules, as well as the underlying synthetic design of both emitters, has been reported previously. As a result, the present contribution relies primarily on extending the characterization of known MR-TADF scaffolds into the two-photon CPL regime, rather than introducing a fundamentally new molecular architecture.

While the authors correctly emphasize the scarcity of organic 2PE-CPL systems, the reported emission dissymmetry factors are moderate and lie within the typical range observed for small-molecule organic CPL emitters, rather than constituting a step-change in performance. In this context, the description of the gpl values as "high" in several instances appears overstated. Similarly, the measured two-photon absorption cross-sections are respectable but remain in the moderate regime when compared with the literature on two-photon-active CPL emitters.

Version 1:

Reviewer comments:

Reviewer #2

(Remarks to the Author)
I thank the authors for the efforts they made to take my previous comments into account. Although I fully understand the difficulties of approaching the current system computationally, some of the results provided in the revised manuscript are not

yet of sufficient quality for publication, especially in a high-impact journal. The agreement between experimental and calculated UV/vis and ECD spectra shown in Figures S20 and S26 is very poor. As an expert of ECD calculations, I would not trust an assignment of absolute configuration based on the comparison shown in Figure S26. Although this is marginal to the paper's overall argument, it impacts its soundness. On the other hand, the agreement between experimental and calculated g(lum) values is pretty good, and the justification added at p. 12 is unnecessary. As far as I understand, both ECD calculations and CPL calculations were run with TDA at the same level. If this is correct, it would be a rather unique case where the g(lum) is predicted much better than g(abs). I kindly ask the authors to clarify the situation, and to add a table to the Supporting Information with calculated parameters (uncorrected wavelength, oscillator and rotational strength) for the first 3 S0-Sn excitations and the S1-S0 emission.

Version 2:

Reviewer comments:

Reviewer #2

(Remarks to the Author)
I thank the authors for further computational efforts. The paper can be published in its current version.

Open Access This Peer Review File is licensed under a Creative Commons Attribution 4.0 International License, which permits use, sharing, adaptation, distribution and reproduction in any medium or format, as long as you give appropriate credit to the original author(s) and the source, provide a link to the Creative Commons license, and indicate if changes were made.
In cases where reviewers are anonymous, credit should be given to 'Anonymous Referee' and the source.
The images or other third party material in this Peer Review File are included in the article’s Creative Commons license, unless indicated otherwise in a credit line to the material. If material is not included in the article’s Creative Commons license and your intended use is not permitted by statutory regulation or exceeds the permitted use, you will need to obtain permission directly from the copyright holder.
To view a copy of this license, visit https://creativecommons.org/licenses/by/4.0/

NCOMMS-26-005008A

Reviewer #1 (Remarks to the Author):

In this paper the authors report two chiral hetero[9]helicene MR-TADF molecules, consisting of two annulated MR-TADF units to a central naphthyl or quinoxanyl group. The two pairs of CP-MR-TADF enantiomers show strong CPL, two-photon absorption, high photoluminescence quantum yields, and long-lived delayed fluorescence. This paper reports some interesting results because there are only few MR-TADF molecules showing the 2PA properties. Especially, the authors think they demonstrate for the first time two-photon CPL of TADF emitters, with their circularly polarized brightness above 0.1 GM, demonstrating their potential for CPL biological imaging. But there are also some problems for this work.

1. Though the authors report the first time two-photon CPL of TADF emitters, the chiral hetero[9]helicene MR-TADF molecule DDCzBPh has been reported by Chen et al. (Angew. Chem. Int. Ed. 2024, 63, e202401835), as they commented in the text. Another one has very similar molecular structure. The synthesis method, crystal determination, photophysical and chiroptical (including the theoretical calculation for electronic, magnetic transition dipole moments) properties are consistent with literature reports. Therefore, the novelty of this research is not very high. The authors believe these materials have potential for CPL biological imaging, but there is no corresponding results to support this point. I noticed that the authors added the sentence “This is supported by a prior report in which (S)-BOD1, with a small |gPL| of 6×10-4, was used as a CPL probe in living cells using CPL-laser scanning confocal microscopy (CPL-LSCM).” in the text, but it is not enough. The results and advantages of these CP-MR-TADF enantiomers for CPL biological imaging are necessary.

Our response: We acknowledge that we were scooped on the reporting of the compound DDCzBPh, published in Angew. Chem. Int. Ed. 2024, 63, e202406497 (a paper focusing on CP-OLEDs, not 2PE CPL as in our case) and Angew. Chem. Int. Ed. 2024, 63, e202401835 (a paper focusing on anion-responsiveness and CPL). However, the core contribution of our work lies in the first demonstration of two-photon CPL from an organic TADF emitter. Our compound represents one of the very few known examples of an MR-TADF emitter exhibiting two-photon absorption and the first to display measurable 2PE-CPL with a relatively large two-photon cross-section. Especially, by strengthening the intramolecular charge transfer (ICT) character of the emissive excited state via embedding the quinoxanyl group for **DDCzBPy**, the 2PA cross section was improved to 142 GM for **DDCzBPy**, compared with 118 GM for **DDCzBPh**.

We agree that direct CPL cell-imaging data would increase the impact of the paper and these materials. However, the CPL-resolved imaging is beyond the scope of the current study and will form the subject of a follow up paper where the focus is the imaging itself

and not the materials design or underpinning photophysical properties discussion. Notably, given the modest low 2PE CPB at this stage, any observable CPL signal under biological conditions would be expected only under single-photon excitation instead of multiphoton conditions. In order to keep the manuscript focused and internally consistent, we have therefore limited our claims to a potential for CPL bioimaging, supported by (1) favorable photophysical properties (high brightness and appreciable g_{PL}) and (2) previous report demonstrate that emitters with modest g_{PL} (such as (S)-BOD1 with $|gPL| \approx 6\times10^{-4}$) can already serve as effective CPL probes in living cells using CPL-laser scanning confocal microscopy, as what we have in the manuscript now.

We hope that this explanation makes our positioning clearer: our contribution is to establish MR-TADF helicenes as promising 2PE-CPL platforms and to elucidate the structure–property relationships relevant to future CPL-bioimaging probe design, while leaving the actual biological validation as an important direction for follow-up studies.

2. In the Introduction section, the authors gave one example of functionalized cationic chiral diaza[4]helicenes, which showed one photon exciton (1PE) gPL values of +5.8/–6.7×10–4 and 2PE gPL values of +9.4/–9.2×10−4. It seems that the 2PE gPL values are higher than that of 1PE gPL data. But in this case, the 2PE gPL values are a little lower than that of 1PE gPL data. The authors should explain this difference from literatures.

Our response: We thank the reviewer for this insightful comment. In principle, the g_{PL} value reflects the intrinsic chiroptical property of the excited state and therefore should be independent of the excitation mechanism. However, in practice, measurement of 2PE-CPL signals is experimentally challenging due to the weak two-photon-induced emission intensity. As a result, the experimental errors can lead to apparent discrepancies between the 1PE and 2PE g_{PL} values.

3. I think the position of Figure 3 is not suitable. In the "Theoretical calculations" section the authors did not mention this figure, but discussed the corresponding results after Figure 5.

Our response： We appreciate the reviewer's comment. We did reference Figure 3 in the "Theoretical Calculations" section ("The calculated $|g_{PL}|$ values are 7×10^{-3} and 2×10^{-3} for DDCzBPh and DDCzBPy, respectively (Figure **3**)"). However, since more detailed discussion of this figure appears later in the manuscript, we have now moved Figure **3** to appear as Figure **5** (page 9) for an improved logical flow.

4. Furthermore, the authors also should pay attention to their writings. For example, In the title, the authors used "Multiresonant", but in the Abstract and text they used "Multi-resonant"; and "Multi-resonant" is an adjective word, which is not suitable as a

Keyword;“ΦF” and “ΦPL” In “To date, dozens of CP-MR-TADF emitters have been reported.”, the full name of “CP-MR-TADF” should be provided; “two-photon excitation (2PE)” appears twice

Our response： We thank the reviewer for pointing out these inconsistencies. All the mentioned issues have been corrected in the revised manuscript as follows:

1. Changed all the multiresonant to multi-resonant throughout for consistency.
2. Replaced "Multiresonant" keyword with " Multi-resonanant TADF".
3. Expanded first instance of "CP-MR-TADF" to "circularly polarized MR-TADF" (page 3).
4. Removed redundant "two-photon excitation (2PE)" definition.

Reviewer #2 (Remarks to the Author):

The manuscript by Pal, Zysman-Colman, and co-workers describes two hetero[7]helicenes exhibiting two-photon (2P) CPL. In light of the limited number of prior reports in this area, and the good–though not exceptional–2P-CPL performance of the present compounds, I believe the work has sufficient significance and novelty to deserve publication in Nature Communications. This holds true even though one of the two compounds has been previously reported, along with its chiroptical properties, although without evaluation of its 2P-CPL activity.

My main concerns relate to the theoretical and computational rationalization of the (chiro)optical properties. The calculations were performed at the PBE0/6-31G(d) level of theory. In a recent review I co-authored on CPL calculations (Chem. Rev., 2025, 125, 10492–10656), we collected extensive literature data on CPL simulations for helicenes. Our survey highlights, among other aspects, the strong dependence of computed results on the choice of functional and basis set. In the present case, the selected functional may not adequately describe the charge-transfer character of the key electronic transitions, while the basis set is too limited in size to provide quantitatively reliable results. Although computational analysis is not the main topic of the manuscript, a more proper choice of the functional and basis set may possibly solve a few inconsistencies listed below.

1) Although the two compounds share the same ground-state symmetry (C□), the symmetry of the S□→S□ transition appears to differ: the transition dipole moment is oriented parallel to the C□ axis in DDCzBPh and orthogonal to it in DDCzBPy. The origin of this discrepancy is not addressed by the authors. A plausible explanation is excited-state desymmetrization in S□, a phenomenon that is particularly relevant for CPL emitters (see review cited above) and deserves a more in-depth analysis.

2) The authors stress a different degree of CT character in the two molecules, which they deem responsible for the discrepancy in gPL values. However, frontier molecular orbitals, transition density plots and NTO's provided in the Supporting Information look extremely similar for the two compounds. One must conclude that the current level of theory is not able to describe properly the ICT state.

3) In line with previous point, the calculated absorption spectra (Fig. S20) do not at all "match well the experimental ones". The shape and relative band intensities are not reproduced, and some bands are apparently missing (including the 1st experimental band for DDCzBPh?)

4) Since the calculation of the absorption profile also provides calculated ECD spectra, as a quality check the authors are asked to compare experimental and calculated ECD spectra, as well.

Summarizing, the authors are kindly asked to address the following points:

(a) Re-run the calculations using a higher level of theory, for example CAM-B3LYP/def2-TZVP, including an appropriate solvent model.

Our response： We thank the reviewer for this valuable suggestion. We fully agree that the use of a higher level of theory is desirable for improving the reliability of the excited-state calculations. In fact, we performed extensive test calculations at the CAM-B3LYP/def2-TZVP level. However, for the present system containing more than 300 atoms, a full excited-state geometry optimization at this level was found to be computationally prohibitive and could not be completed within practical computational limits. For systems of this size, hybrid-DFT calculations can become impractical, and multi-level/composite strategies are commonly used as a reasonable compromise between accuracy and feasibility (*Adv. Mater.* **2022**, *34*, 2207416). Therefore, in the revised manuscript we adopted a composite protocol: the geometries were optimized at the CAM-B3LYP/6-31G(2d,p) level, while the investigation of the excited states was calculated at the TDA-DFT-CAM-B3LYP/def2-TZVP level with the solvent model (PCM, Toluene) included. This strategy enables us to retain the higher-level description for the key excited-state properties while keeping the calculations feasible for such a large molecular system. We have clarified this point in the revised Computational Details section. Now the Calculation results were replaced by the new calculation results.

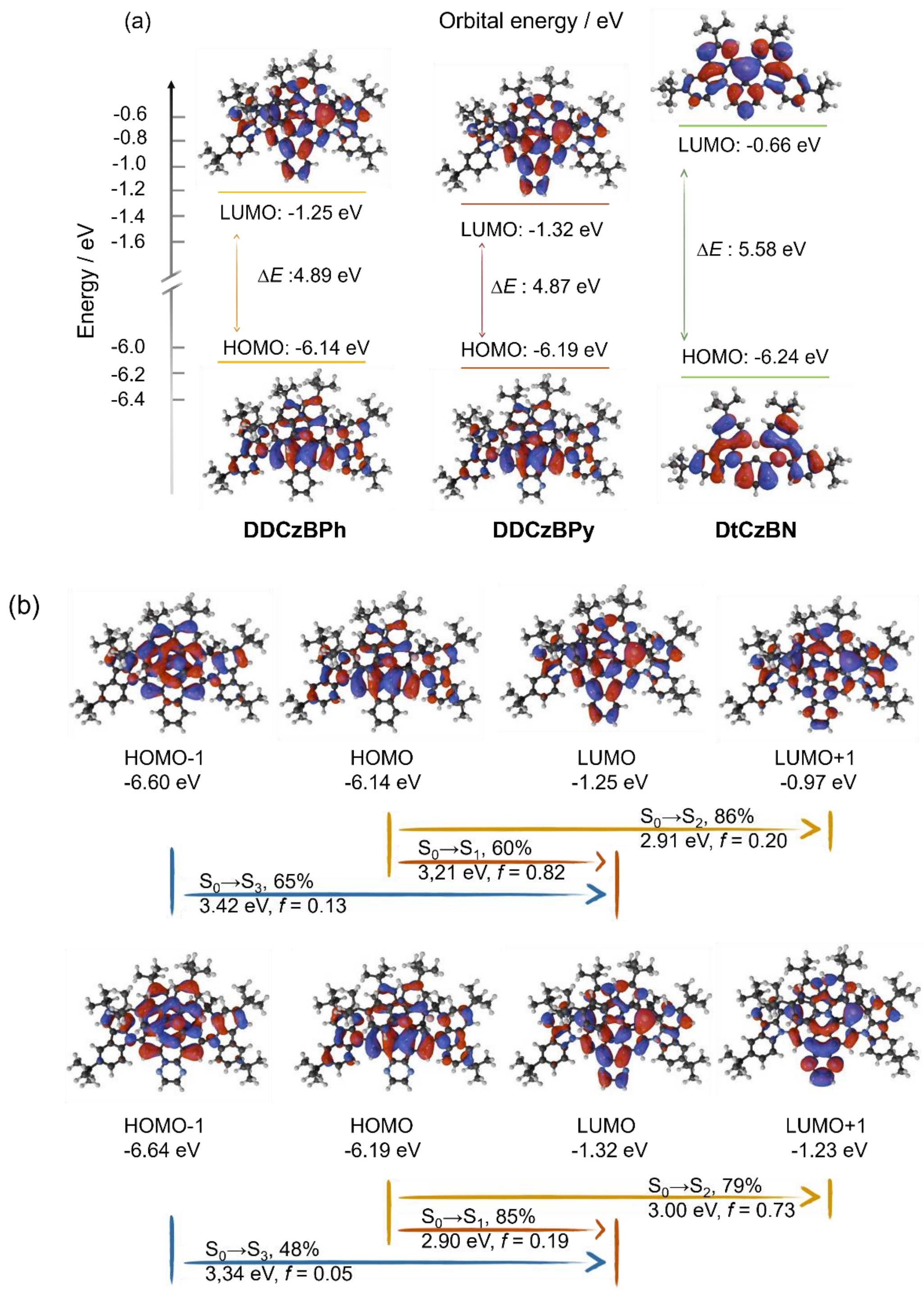

Figure **S18**. Theoretical modeling of **DDCzBPh** and **DDCzBPy** in the toluene solution at the CAM-B3LYP/def2-TZVP level. (a) the energies and electron density distributions of the HOMO/LUMO (ISO value = 0.02); (b) the major molecular orbitals in the three lowest singlet states, along with the energy levels and contributions. *f* is the oscillator strength.

(b) Provide updated comparisons between calculated and experimental absorption *and* ECD spectra.

Our response： Thanks for your suggestion. In response, we have updated the comparison between the calculated and experimental absorption and ECD spectra in the revised manuscript. The results obtained at the CAM-B3LYP/def2-TZVP level show a pronounced overestimation of the transition wavelengths relative to both the PBE0/6-31G(d) results and the experimental spectra, although the nature of the excited states is reproduced reasonably well. Therefore, to enable a more meaningful visual comparison between the simulated and experimental spectra, we applied a constant wavelength correction to the calculated spectra. Specifically, shifts of 104 nm for DDCzBPh and 98 nm for DDCzBPy were used in the plotted absorption and ECD spectra.

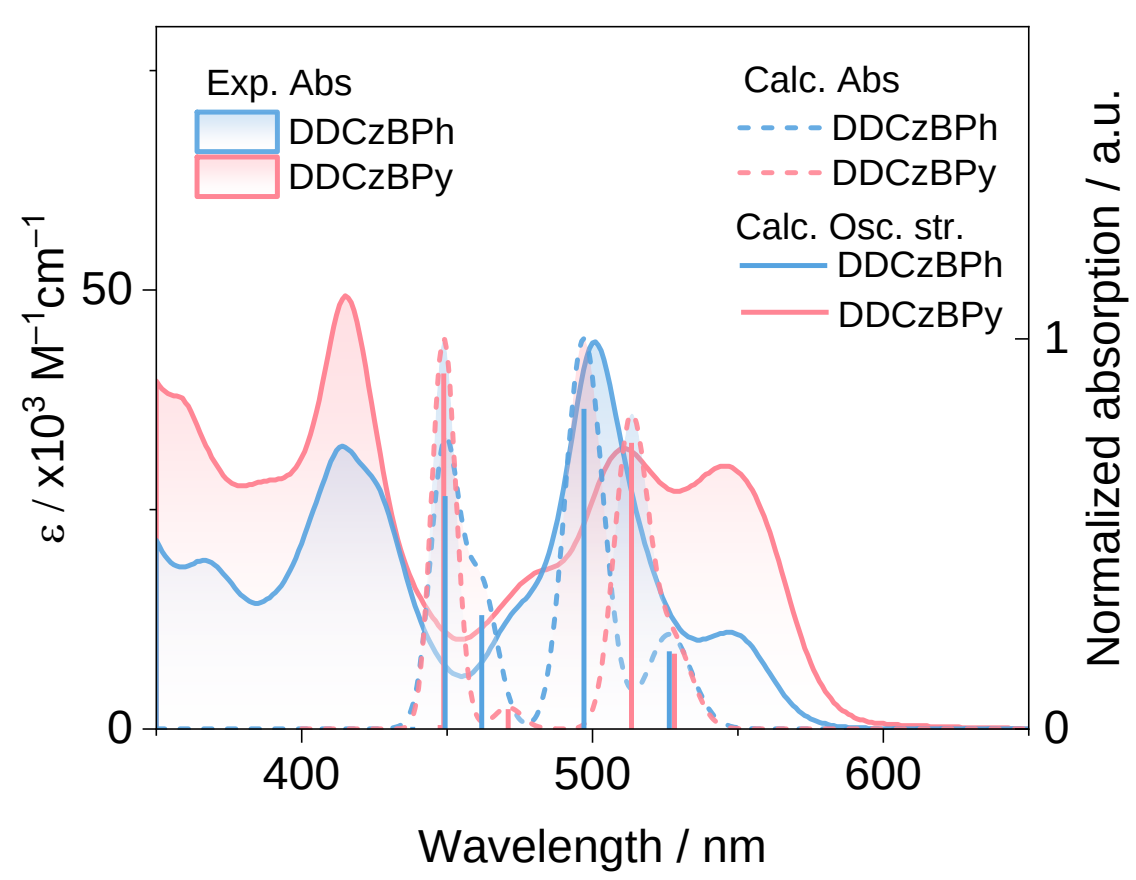

Figure **S20**. Simulated absorption spectra with a Gaussian function having FWHM of 0.10 eV (dashed line) and experimental absorption spectra in toluene (solid line). The simulated absorption spectra were generated by applying a Gaussian band shape with a half-bandwidth of 0.05 eV. To correct for the systematic overestimation of the excitation energies by the TDA-DFT method, a constant wavelength shift of 104 nm for **DDCzBPh** and 98 nm for **DDCzBPy**, determined by aligning the calculated UV maxima with the experimental ones (501 nm for **DDCzBPh** and 511 nm for **DDCzBPy**), was applied to the calculated absorption spectra.

“The absolute configurations were inferred by comparing the experimental circular dichroism spectra with predicted spectra based on TDA-DFT calculations at the CAM-B3LYP/def2-TZVP level.”

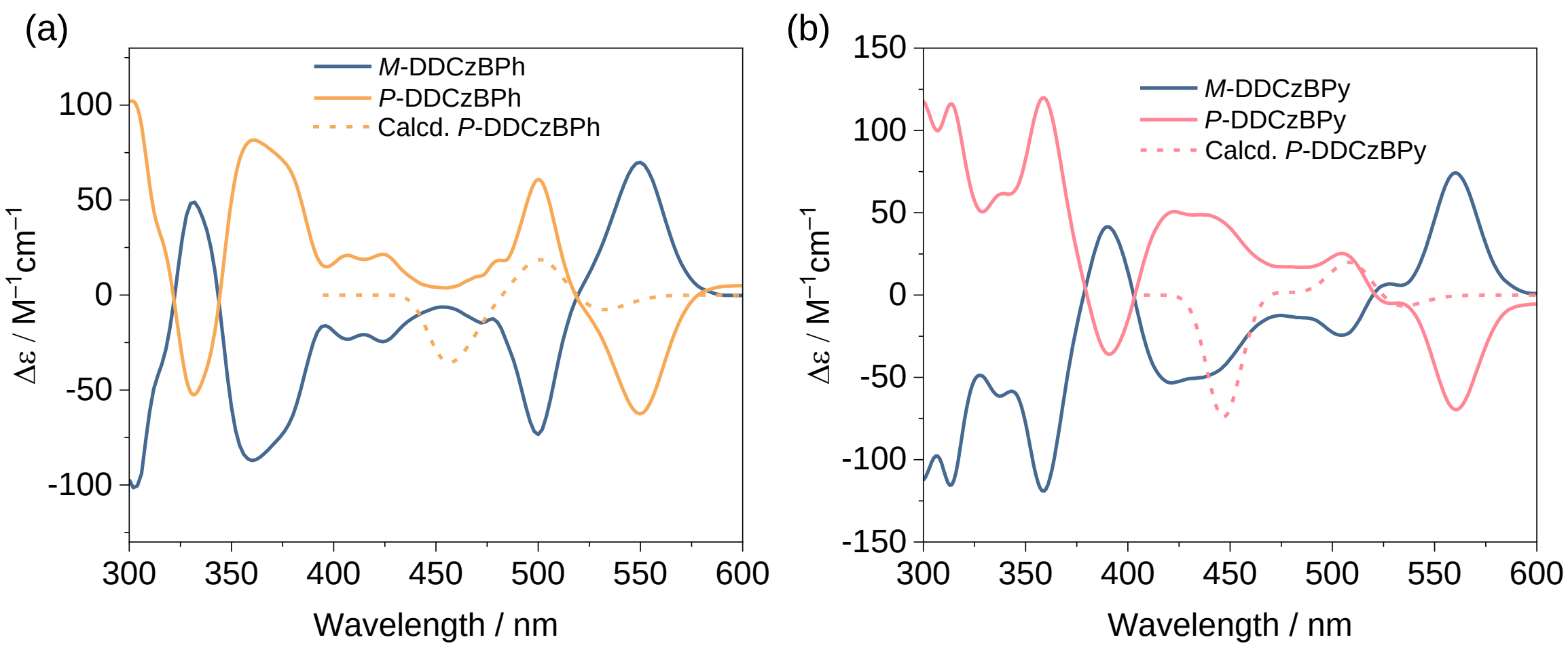

Figure **S26**. Experimental and calculated ECD spectra of (a) P/M-DDCzBPh and (b) P/M-DDCzBPy measured in toluene. Predicted spectra were calculated at the CAM-B3LYP/def2-TZVP with an implicit toluene solvent field (IEFPCM). The calculated ECD spectra were generated by applying a Gaussian band shape with a FWHM of 0.1 eV. The same correction to absorption was applied to the calculated ECD spectra.

(c) Reassess the nature of the S□→S□ transition.

(d) Discuss the (possible) desymmetrization of the S□ excited state.

(e) If warranted, include a quantitative evaluation of the charge-transfer character using one of the established metrics available in the literature.

Our response: We thank the Reviewer for this insightful suggestion. In the revised manuscript, we have recalculated the excited-state properties at the TDA-CAM-B3LYP/def2-TZVP level and included a quantitative discussion of the S_1 excited-state character in the manuscript (See below).

"The NTO analysis indicates that the S1 states of both emitters are predominantly short-range charge-transfer (SRCT) in nature, with only limited intramolecular charge-transfer character between the peripheral phenyl/pyridyl unit and the DCzBN backbone. Furthermore, both emitters exhibit similar, approximately C_2-symmetric transition patterns rather than pronounced symmetry breaking, which is consistent with the similar orientations of the electric and magnetic transition dipole moments (Figure 5). The calculated g_{PL} value reflects the intrinsic chiral luminescence of an idealized isolated molecule in the excited state, whereas the experimental value is affected by conformational distribution, vibrational averaging, solvent or matrix polarization, and

intermolecular interactions. Hence, the experimental g_{PL} is reasonably slightly smaller than the calculated value. For DDCzBPy, although the S_1 state is still predominantly SRCT in nature, its greater sensitivity to environment due to its larger charge transfer distance (D_{CT} =0.618 Å for DDCzBPy and D_{CT}=0.268Å for DDCzBPh) may account for the noticeably lower experimental g_{PL} than the calculated value."

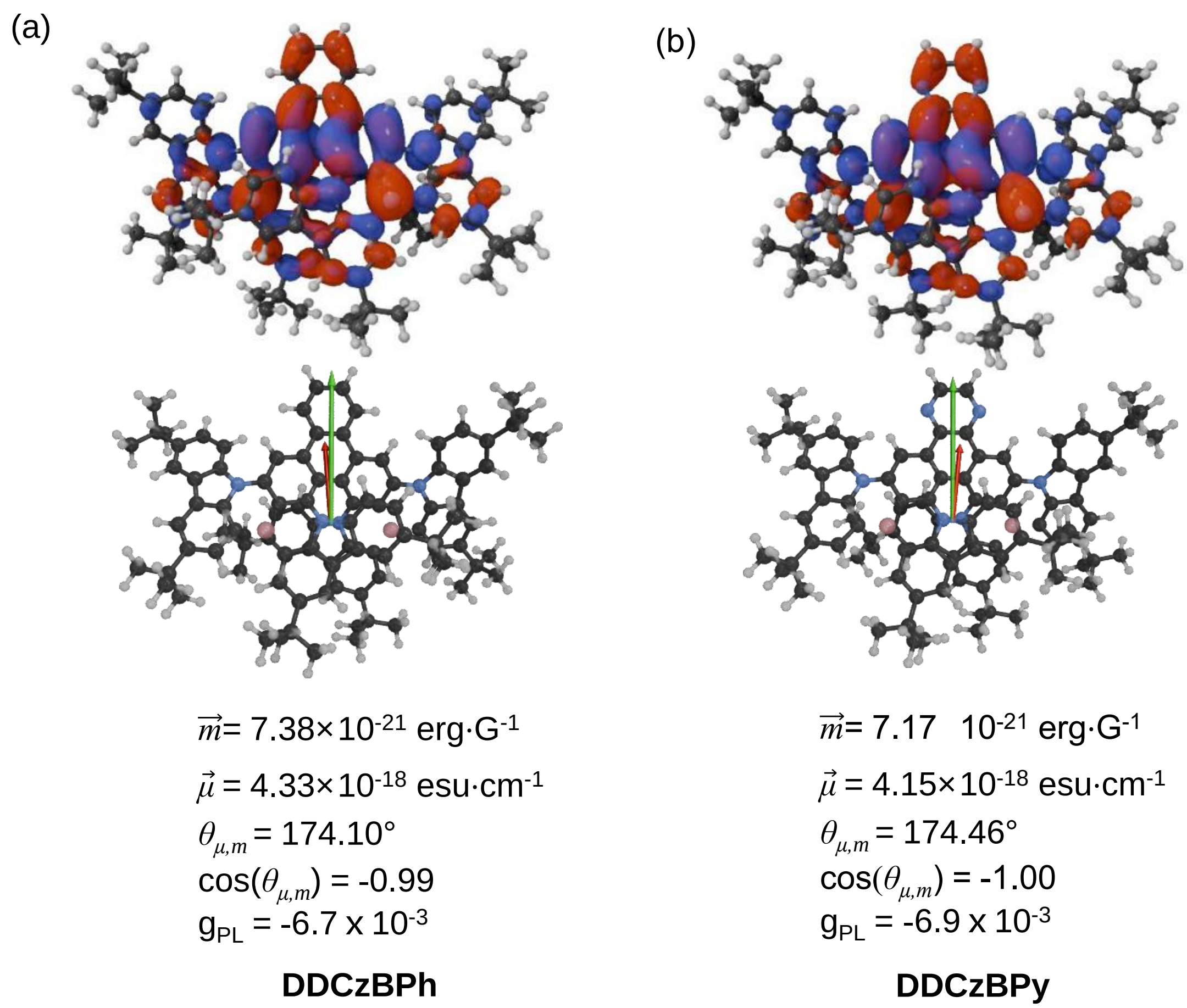

Figure **5**. NTOs (particle and hole are represented by red and blue colors, respectively) and theoretical predicted electronic (red arrow), magnetic (green arrow) transition dipole moment and their angles during the S_1 to S_0 transition processes, and predicted g_{PL} values based on the optimized S_1 geometries at the CAM-B3LYP/def2-TZVP level in the toluene for (a) **DDCzBPh** and (b) **DDCzBPy**.

From an experimental perspective, I note that the two-photon excitation spectra lack long-wavelength bands. Could the laser excitation range be extended to probe these regions as well?

Our response： For the two-photon excitation spectra, now we have the excitation range of 750-1200 nm for **DDCzBPy**. There seems to be no absorption beyond 850 nm, therefore we did not extend the measurement to longer wavelengths for **DDCzBPh**.

Finally, a more critical comparison between the data reported for DDCzBPh and those from the previous study (ref. 13) would be useful. While most parameters are in good agreement, this is not the case for CPB, where the reported values differ by approximately a factor of 3.

Our response： The circularly polarized brightness (CPB) is defined as CPB = $\varepsilon_{abs} \times \Phi_{PL} \times (|g_{PL}| / 2)$, which depends on three key parameters: the molar absorption coefficient (ε_{abs}), photoluminescence quantum yield (Φ_{PL}), and dissymmetry factor (g_{PL}). Our measured g_{PL} values (-5.5×10^{-3} and 5.8×10^{-3}) are consistent with those reported in the literature (Angew. Chem. Int. Ed. 2024, 63, e202401835). However, our ε_{abs} and Φ_{PL} values (44.09×10^{3} M^{-1} cm^{-1} and 65%) are notably lower than the reported values (~80×10^{3} M^{-1} cm^{-1} and 98%), which explains the discrepancy in CPB (approximately a factor of 3). Importantly, when compared with another publication reporting the same molecular structure (Angew. Chem. Int. Ed. 2024, 63, e202406497, $\varepsilon_{abs} \approx 47\times10^{3}$ M^{-1} cm^{-1} and $\Phi_{PL} \approx 78\%$), our results ($\varepsilon_{abs} \approx 44\times10^{3}$ M^{-1} cm^{-1} and $\Phi_{PL} \approx 65\%$) are in good agreement. We therefore attribute the difference mainly to instrumental or experimental variations rather than a true material inconsistency.

Reviewer #3 (Remarks to the Author):

In this manuscript, the authors report two-photon-excited circularly polarized luminescence (2PE-CPL) from MR-TADF emitters. The experimental work is of high quality: the synthesis, chiral resolution, and comprehensive photophysical and chiroptical characterization are carefully executed and convincingly presented.

With respect to novelty and positioning within the literature, the principal advance is the first demonstration of 2PE-CPL in an MR-TADF molecular system. However, one of the investigated molecules, as well as the underlying synthetic design of both emitters, has been reported previously. As a result, the present contribution relies primarily on extending the characterization of known MR-TADF scaffolds into the two-photon CPL regime, rather than introducing a fundamentally new molecular architecture.

While the authors correctly emphasize the scarcity of organic 2PE-CPL systems, the reported emission dissymmetry factors are moderate and lie within the typical range observed for small-molecule organic CPL emitters, rather than constituting a step-change

in performance. In this context, the description of the gpl values as “high” in several instances appears overstated. Similarly, the measured two-photon absorption cross-sections are respectable but remain in the moderate regime when compared with the literature on two-photon-active CPL emitters.

Our response: We thank the reviewer for the positive assessment of our work.

As we indicated in our response to Reviewer 1, We acknowledge that we were scooped on the reporting of the compound **DDCzBPh**, published in Angew. Chem. Int. Ed. 2024, 63, e202406497 (a paper focusing on CP-OLEDs, not 2PE CPL as in our case) and Angew. Chem. Int. Ed. 2024, 63, e202401835 (a paper focusing on anion-responsiveness and CPL). However, the core contribution of our work lies in the first demonstration of two-photon CPL from an organic TADF emitter. Our compound represents one of the very few known examples of an MR-TADF emitter exhibiting two-photon absorption and the first to display measurable 2PE-CPL with a relatively large two-photon cross-section. Especially, by strengthening the intramolecular charge transfer (ICT) character of the emissive excited state via embedding the quinoxanyl group for **DDCzBPy**, the absorption crossing was improved to 142 GM for **DDCzBPy**, compared with 118 GM for **DDCzBPh**. Figure **S1** summarizes examples of recently reported CP-MR-TADF emitters, and most of them have g_{PL} less than 2×10^{-3}, so a g_{PL} 5.2×10^{-3} therefore reasonably can be regarded as comparatively high within this class of emitters.

Notably, in Angew. Chem. Int. Ed. 2023, 62,e202312326, Adachi and co-workers focused on the study of Anti-Stokes Luminescence (ASPL) in MR-TADF emitters (**DABNA-2**, **CzBN**, and **CzBSe**). The two-photon absorption properties of these compounds were discovered when the authors eliminated the possibility of the TPA-assisted ASPL in the excitation condition. The cross-sections of these emitters all show relatively low values (e.g., only 35 GM for the parent emitter **CzBN** upon which our materials are based). To improve the cross-section and extend the multi-function of the MR-TADF emitters, we designed these two helical structures by extending the conjugation and tuning the ICT character. Here, we report two emitters that show quite high cross-sections (142 GM), high g_{PL} (5.2×10^{-3}) and 2PE CPL, with 2PE-CPB of 0.20 GM.

The 2PE CPB value reported here is among the highest for circularly polarized luminescent materials, surpassing those of reported emitters (0.05 GM for a fluorescent emitter in Chem. Eur. J. 2025, 31, e202501212; 0.16 GM for Eu complex in *J. Am. Chem. Soc.* 2023, 145, 46, 25170–25176) benefited from synergistically higher 2PA cross-section (σ^2) and photoluminescence quantum yield (Φ_{PL}) of MR-TADF with extended conjugation.

The use of chiroptical information for multiplexed imaging in biology could provide a step-change in sensitivity, and our work is a necessary first step towards demonstrating

this possibility, and with organic emitters to boot. This represents a crucial step toward broadening the use of MR-TADF emitters in bioimaging, leveraging their superior advantages: high Φ_{PL}, extended delayed lifetimes, and theoretically 100% exciton utilization.

NCOMMS-26-005008B

REVIEWER COMMENTS

Reviewer #2 (Remarks to the Author):

I thank the authors for the efforts they made to take my previous comments into account. Although I fully understand the difficulties of approaching the current system computationally, some of the results provided in the revised manuscript are not yet of sufficient quality for publication, especially in a high-impact journal. The agreement between experimental and calculated shown in Figures S20 and S26 is very poor. As an expert of ECD calculations, I would not trust an assignment of absolute configuration based on the comparison shown in Figure S26. Although this is marginal to the paper's overall argument, it impacts its soundness. On the other hand, the agreement between experimental and calculated g(lum) values is pretty good, and the justification added at p. 12 is unnecessary. As far as I understand, both ECD calculations and CPL calculations were run with TDA at the same level. If this is correct, it would be a rather unique case where the g(lum) is predicted much better than g(abs). I kindly ask the authors to clarify the situation, and to add a table to the Supporting Information with calculated parameters (uncorrected wavelength, oscillator and rotational strength) for the first 3 S_0-S_n and S_1-S_0 the emission.

Answer: We thank the Reviewer for acknowledging our efforts to improve this manuscript. We also recognize that the agreement between the experimental and calculated UV/vis and ECD spectra in the previous version was not satisfactory. To address this issue, we have re-evaluated our computational protocol and recalculated the spectra at the TD-DFT-B3LYP/def2-SVP level with an implicit toluene solvent model. The revised results show a noticeably improved agreement between the experimental and calculated UV/vis absorption and ECD spectra.

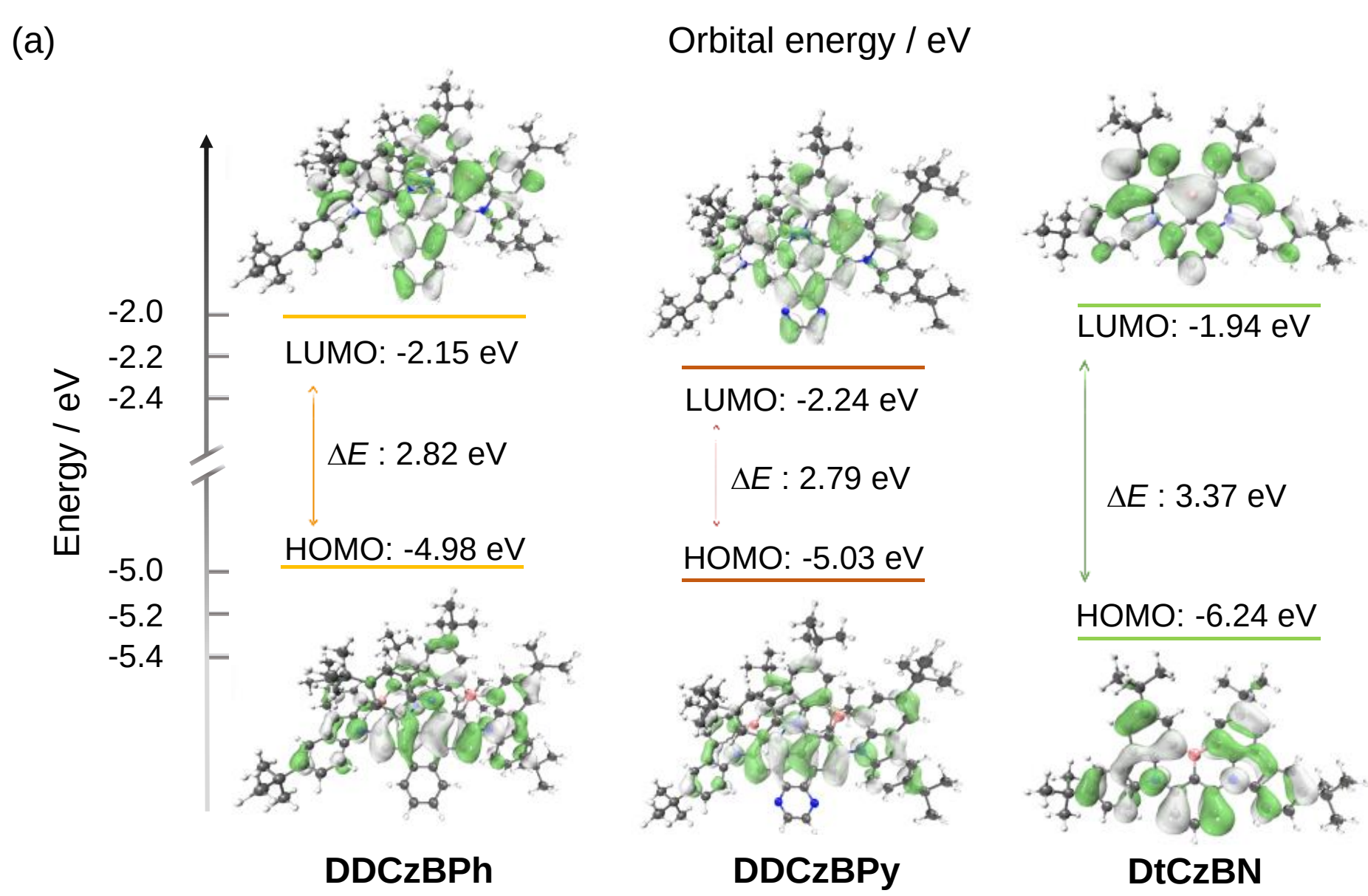

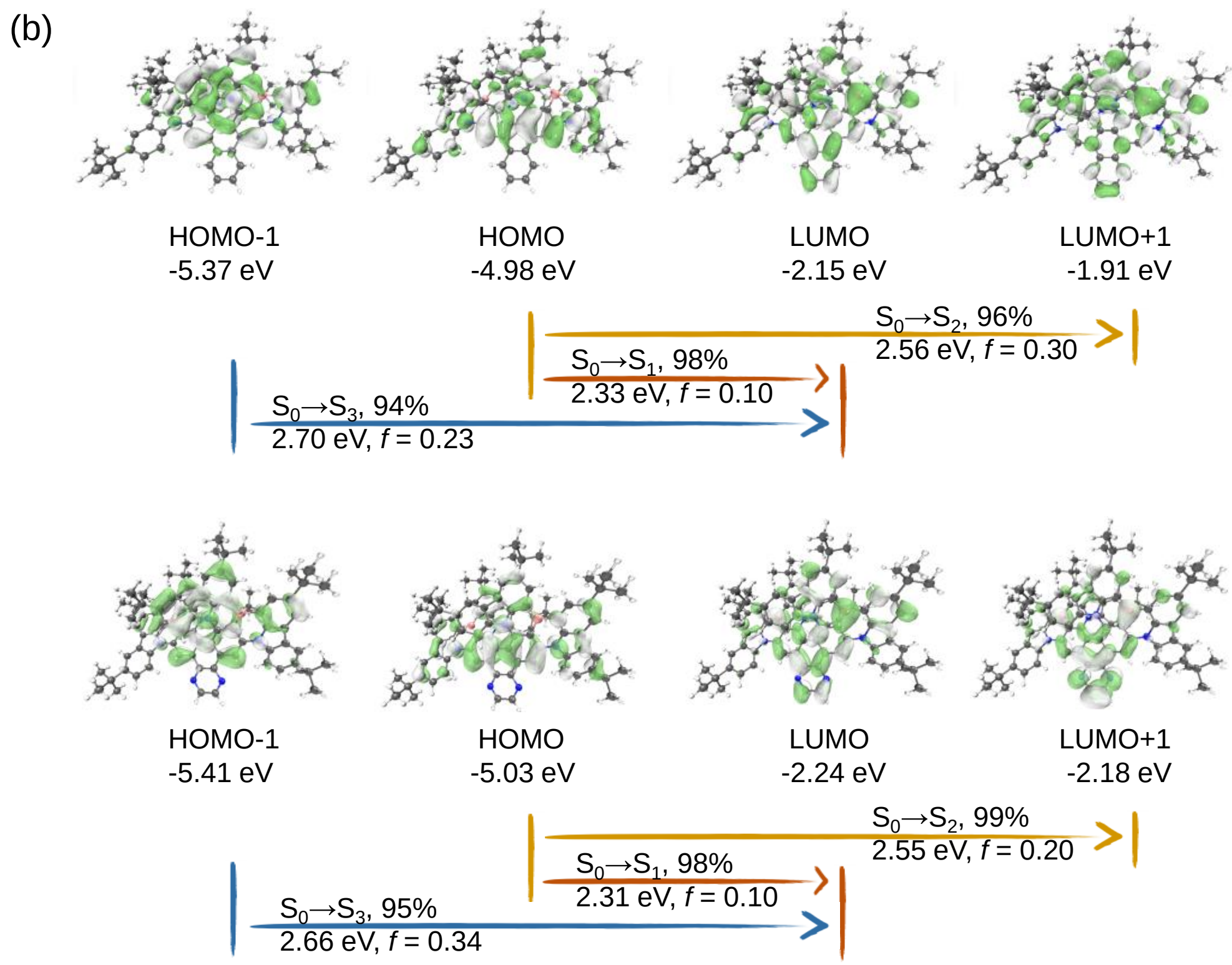

Figure **S18**. Theoretical modeling of **DDCzBPh** and **DDCzBPy** in the toluene solution at the B3LYP/def2-SVP level. (a) the energies and electron density distributions of the HOMO/LUMO (ISO value = 0.02); (b) the major molecular orbitals in the three lowest singlet states, along with the energy levels and contributions. *f* is the oscillator strength.

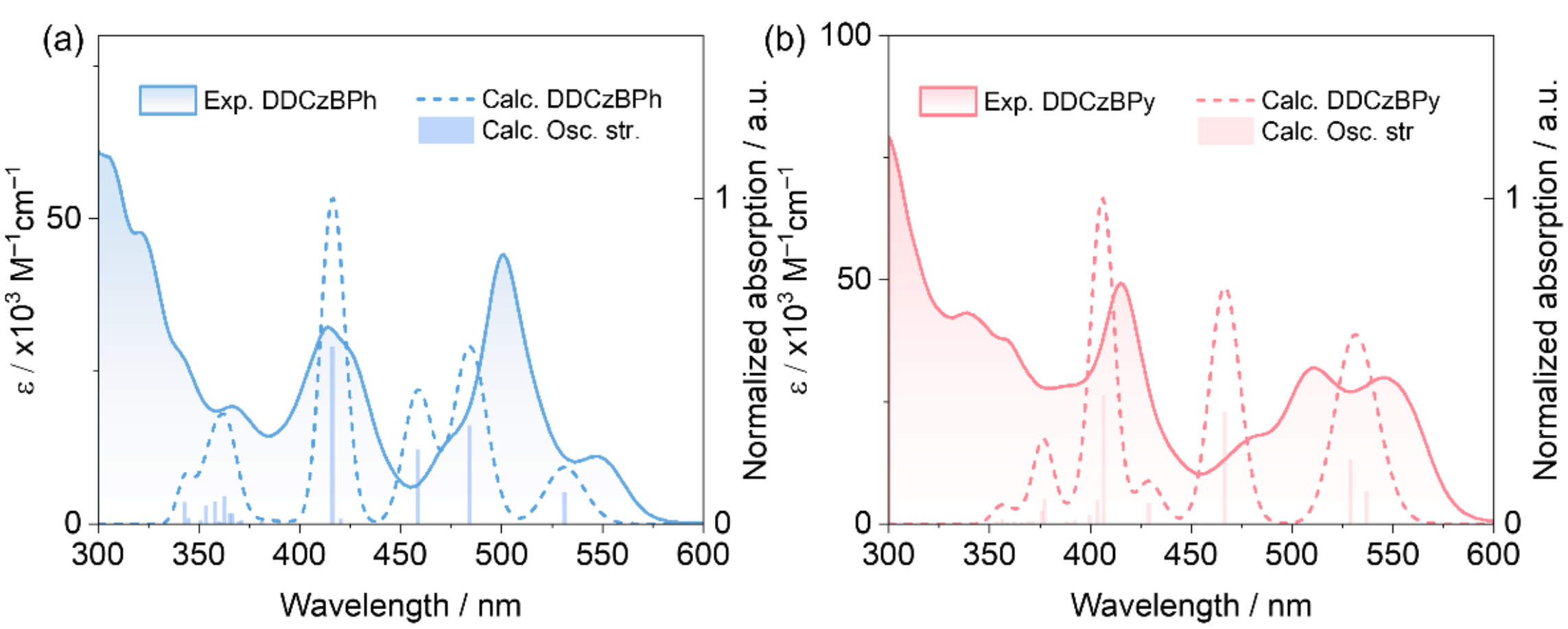

Figure **S20**. Simulated absorption spectra with a Gaussian function having FWHM of 0.05 eV (dashed line) and experimental absorption spectra in toluene (solid line) for (a) **DDCzBPh** and (b) **DDCzBPy**.

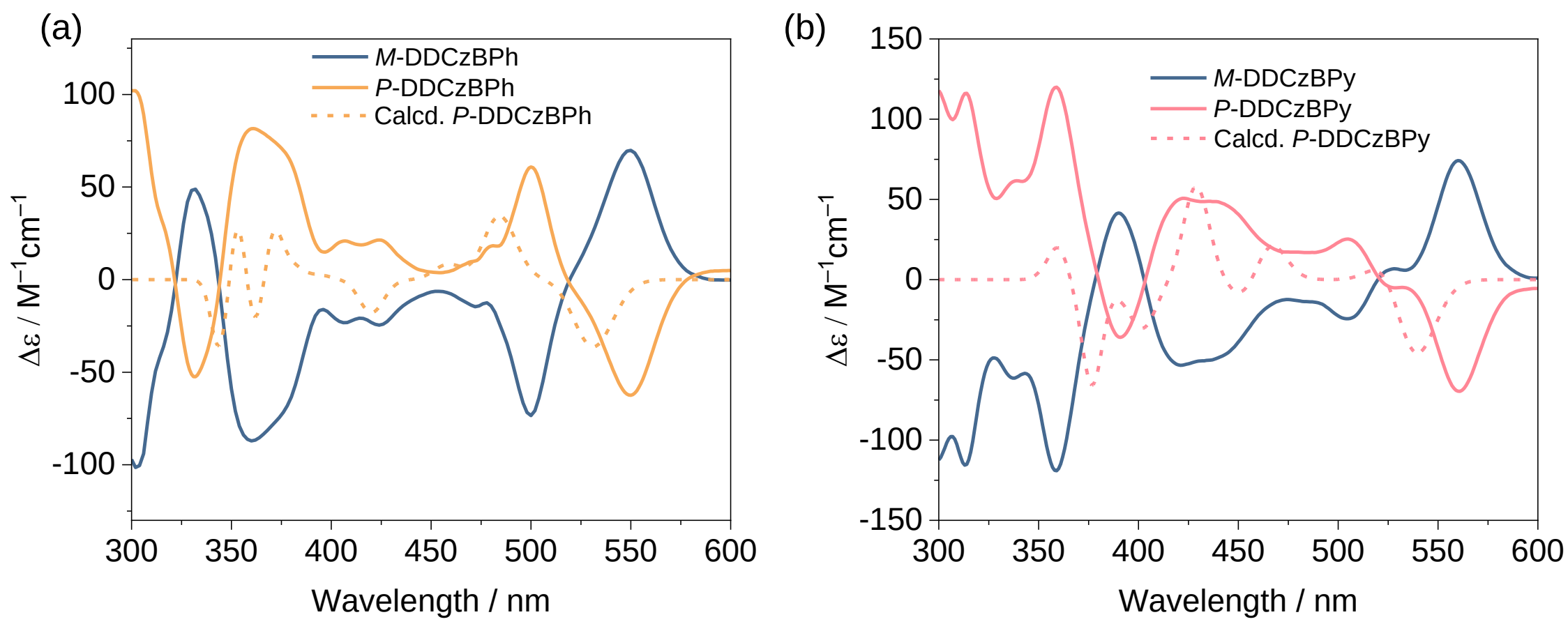

Figure **S26**. Experimental and calculated ECD spectra of (a) ***P-/M*-DDCzBPh** and (b) ***P-/M*-DDCzBPy** measured in toluene. Predicted ECD spectra were calculated at the B3LYP/def2-SVP with an implicit toluene solvent field (IEFPCM).

Regarding the Reviewer's comment on the discussion on page 12, we have simplified the text to avoid overinterpretation.

"The NTO analysis indicates that the S_1 states of both emitters are predominantly SRCT in nature, with only limited intramolecular long-range charge-transfer (LRCT) character between the phenyl/pyridyl unit and the **DtBuCzB** backbone. Furthermore, both emitters exhibit similar, approximately C_2-symmetric transition patterns rather than pronounced symmetry breaking (Figure **5**). For **DDCzBPy**, although the S_1 state is still predominantly SRCT in nature, its larger charge transfer distance (D_{CT} =0.679 Å for **DDCzBPy** and D_{CT}=0.270Å for **DDCzBPh**) may account for the noticeably lower experimental g_{PL} than the calculated value.[30]"

In addition, we have added a new table to the Supporting Information listing the calculated uncorrected wavelengths, oscillator strengths, and rotational strengths for the first three S_0-S_n excitations, as well as for the S_1-S_0 emission. For completeness, the TDA-CAM-B3LYP/def2-TZVP results are also provided for comparison below.

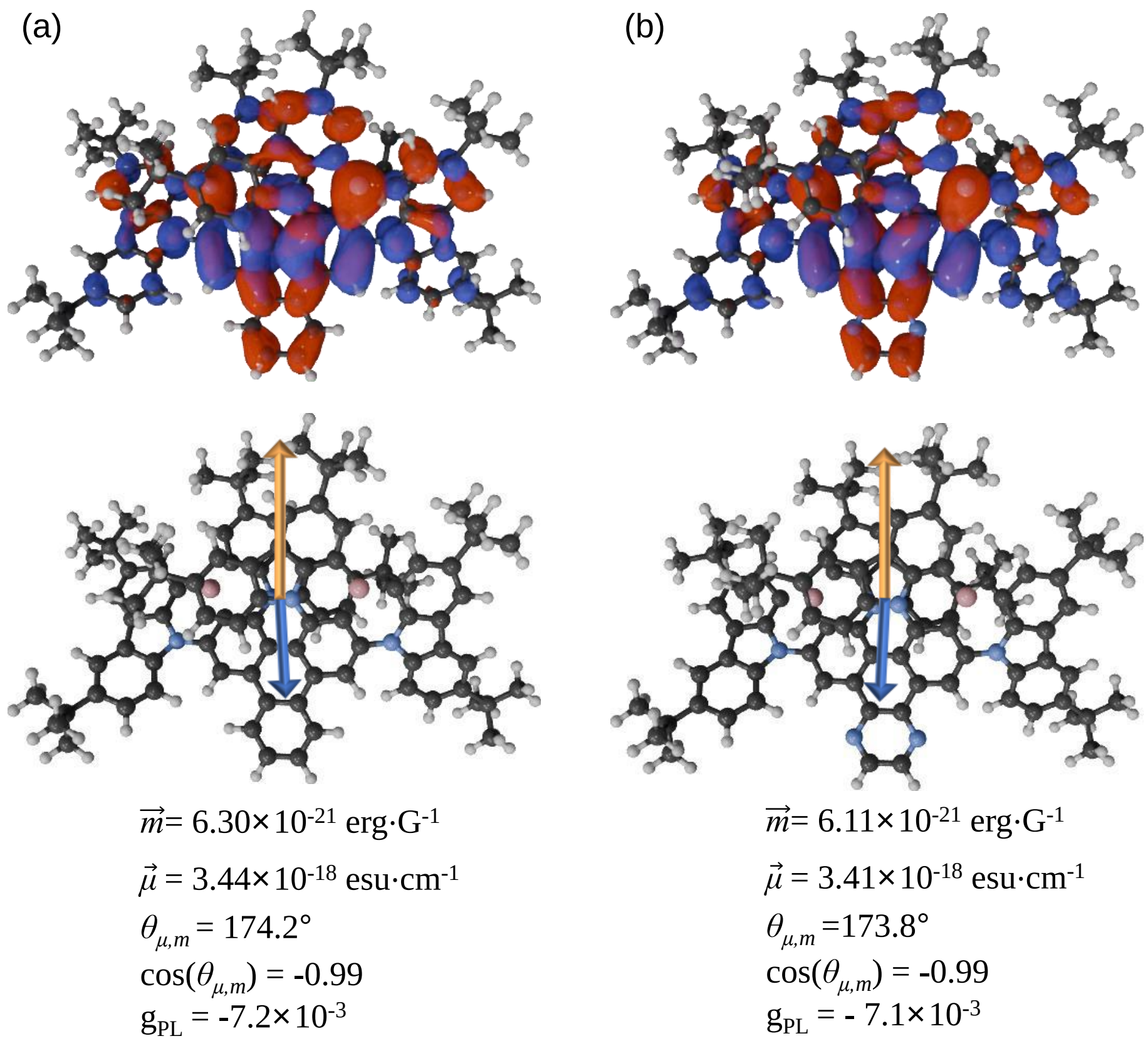

DDCzBPh **DDCzBPy**

Figure **5**. NTOs (particle and hole are represented by red and blue colors, respectively) and theoretical predicted electronic (yellow arrow), magnetic (blue arrow) transition dipole moment and their angles during the S_1 to S_0 transition processes, and predicted g_{PL} values based on the optimized S_1 geometries at the B3LYP/def2-SVP level in the toluene for a) **DDCzBPh** and b) **DDCzBPy**.

Table **S2**. The summary of the calculated parameters for **DDCzBPh** and **DDCzBPy**.

Transitions	Wavelength / nm	f^a	μ/ x 10^{-18} /esu·cm^{-1} [b]	m/ x 10^{-21} erg·G^{-1} [c]	cos($\theta_{\mu,m}$)	$g_{lum/abs}$
			**DDCzBPh**			
S_0-S_1	531	0.0968	3.31	6.88	-1.00	-0.008
S_0-S_2	484	0.3027	5.58	5.77	0.61	0.003
S_0-S_3	458	0.3027	4.72	1.00	0.97	0.001
S_1-S_0	593	0.0936	3.44	6.30	-0.99	-0.007
			**DDCzBPy**			
S_0-S_1	537	0.1006	3.39	6.42	-0.99	-0.008
S_0-S_2	529	0.1966	4.70	2.63	0.75	0.002
S_0-S_3	467	0.3435	5.84	1.40	0.82	0.001
S_1-S_0	597	0.0918	3.41	6.11	-0.99	-0.007

[a] Oscillator strength; [b] Electronic transition dipole moment; [c] Magnetic transition dipole moment; Calculated with TD-DFT- B3LYP/def2-SVP level in toluene.

The calculated results with TDA-DFT-CAM-B3LYP/def2-TZVP in toluene.

The summary of the calculated parameters for **DDCzBPh** and **DDCzBPy**.

Transitions	Wavelength / nm	f^a	μ/ x 10^{-18} /esu·cm^{-1} [b]	m/ x 10^{-21} erg·G^{-1} [c]	cos($\theta_{\mu,m}$)	$g_{lum/abs}$
			**DDCzBPh**			
S_0-S_1	426	0.1989	4.25	8.86	-0.99	-0.008
S_0-S_2	397	0.8206	8.32	5.33	0.98	0.003
S_0-S_3	362	0.2920	4.74	1.72	-0.00	<0.001
S_1-S_0	473	0.1861	4.33	7.38	-0.99	-0.007
			**DDCzBPy**			
S_0-S_1	428	0.1929	4.19	8.28	-1.00	-0.008
S_0-S_2	413	0.7334	8.03	4.07	0.79	0.002
S_0-S_3	371	0.0507	2.00	9.28	0.89	0.016
S_1-S_0	473	0.1711	4.15	7.17	-1.00	-0.007

[a] Oscillator strength; [b] Electronic transition dipole moment; [c] Magnetic transition dipole moment; Calculated with T TDA-DFT-CAM-B3LYP/def2-TZVP level.
